# Comparative genomic analysis of the* COBRA* genes in six Rosaceae species and expression analysis in Chinese white pear (*Pyrus bretschneideri*)

**DOI:** 10.7717/peerj.13723

**Published:** 2022-07-19

**Authors:** Yu Zhao, Xueqiang Su, Xinya Wang, Mengna Wang, Xiaofeng Feng, Muhammad Aamir Manzoor, Yongping Cai

**Affiliations:** 1 School of Life Science, Anhui Agricultural University, Hefei, China; 2 Institute of Sericulture, Anhui Academy of Agricultural Sciences, HeFei, China

**Keywords:** Phylogenetic analysis, COBRA, Rosaceae species, *Pyrus bretschneideri*, Secondary cell wall (SCW)

## Abstract

*COBRA-Like (COBL)* genes encode a glycosylphosphatidylinositol (GPI) anchoring protein unique to plants. In current study, 87 *COBRA* genes were identified in 6 Rosaceae species, including *Pyrus bretschneideri* (16 genes), *Malus domestica* (22 genes), *Fragaria vesca* (13 genes), *Prunus mume* (11 genes), *Rubus occidentalis* (13 genes) and *Prunus avium* (12 genes). We revealed the evolution of the *COBRA* gene in six Rosaceae species by phylogeny, gene structure, conservative sequence, hydrophobicity analysis, gene replication events and sliding window analysis. In addition, based on the analysis of expression patterns in pear fruit combined with bioinformatics, we identified *PbCOBL12* and *PbCOBL13* as potential genes regulating secondary cell wall (SCW) formation during pear stone cell development. This study aimed to understand the evolutionary relationship of the *COBRA* gene in Rosaceae species, clarify the potential function of *COBRA* in pear fruit development, and provide essential theoretical basis and gene resources for improving pear fruit quality through genetical modification mechanism.

## Introduction

Plant secondary wall is mainly composed with cellulose, hemicellulose and lignin. In the development of fruit growth of Dangshan su pear, the continuous accumulation of secondary walls will form stone cells near the core (Su et al., 2019b). The diameter and number of stone cell clusters greatly influence the fruit quality of Dangshan su pear (Zhang et al., 2017). The formation of the secondary wall is a complex dynamic process (Yan et al., 2014). Identifying the primary genes involved in the lignin and cellulose production pathway is crucial for comprehending the secondary wall biosynthesis. In the Dangshan su pear, the critical genes of the lignin biosynthesis pathway are completely characterized, but the cellulose biosynthesis genes are poorly recognized (Su et al., 2019b; Cheng et al., 2018; Cheng et al., 2017; Cheng et al., 2020; Cheng et al., 2019a; Cheng et al., 2019b; Li et al., 2019; Li et al., 2020b). Since then, 36 members of the *CesA* gene family have been isolated from Dangshan su pear, of which four genes may be involved in secondary wall formation (Li et al., 2020a). Except for cellulose synthase, members of the *COBRA* gene family, which encode glycosylphosphatidylinositol (GPI)-anchored proteins, have been identified as new players in the regulation of the orientation of cell expansion in the plant cell wall, these proteins were involved in cell elongation, secondary wall thickening, plant root development and seed coat morphological changes (Roudier et al., 2005; Schindelman et al., 2001).

The *COBRA* gene, which encodes 454 amino acids, plays an important role in plant cell elongation cellulose synthesis and cellulose deposition. The *AtCOBL4* gene was involved in the synthesis of cellulose in the cell wall, although the phenotype of *Atcobl4* was normal, the stem was easy to break (Brown et al., 2005). In the *COBRA* family reported in *Arabidopsis*, *AtCOBL2* was involved in depositing cellulose in seed coat cells (Ben-Tov et al., 2015). In *Arabidopsis thaliana*, when *AtCOBL5* was functionally absent, growth and development were affected, and a large amount of stress-responsive substances were produced, while defense-related gene expression appeared up-regulated (Ko et al., 2006). *AtCOBL9* gene deletion resulted in shorter and less numerous root hairs (Jones, Raymond & Smirnoff, 2006). *COBL* (COBRA like) family members have similar functions in rice. The rice *BC1* (*Brittle culm 1*) gene was up to 60.7% homologous to the *Arabidopsis COB* gene. *bc1* plants had reduced cell wall thickness, reduced cellulose content, and reduced stalk stiffness (Dai et al., 2009; Li et al., 2003). The *bc1* gene affected cellulose assembly by binding microfibrils and ultimately regulated cellulose crystallite size (Liu et al., 2013). A previous study found that the *OsBC1L4* gene was mainly localized in the cell wall and cell membrane, and its mutants had abnormal cell swelling and reduced cellulose content (Dai et al., 2011). The *OsBC1L5* gene was involved in secondary wall synthesis in thick-walled tissues of stem nodes. At the same time, *Osbc1l5* loss of function resulted in severely impaired male gametophyte transport (Dai et al., 2009). *OsBC1L6* regulated *β*-glucan synthesis during endosperm cell wall formation by interacting with cellulose moieties on the plasma membrane during seed maturing (Midorikawa et al., 2019). The *COBRA* family has been reported in maize, and maize *BK2* can ultimately affect stalk strength by influencing cellulose deposition in the secondary wall (Ching et al., 2006). Maize *Roothairless3* (*Rth3*) was homologous to rice *BC1L1*, and *RTH3* was highly expressed in root epidermal cells, root hair cells and lateral root primordia. The mutants of this gene do not have normal root hair development along with reduced field yield (Hochholdinger et al., 2008). Heterologous overexpression of cotton *GhCOBL9A* plants upregulated *CESA* gene expression and cellulose deposition while promoting longitudinal elongation of hypocotyl and root cells during early development (Niu et al., 2018).

Rosaceae plants are widely distributed in China and have significant economic value, Such as pear (*Pyrus bretschneideri*), strawberry (*Fragaria vesca*), black raspberry (*Rubus occidentalis*), sweet cherry (*Prunus avium*), apple (*Malus domestica*), Japanese apricot (*Prunus mume*) belong to Rosaceae family. Previous studies have found that the *COBRA* gene family was identified in Arabidopsis (*Arabidopsis thaliana*), rice (*Oryza sativa*), maize (*Zea mays*) and cotton (*Gossypium raimondii*) with 12, 11, nine, and 19 members, respectively (Borner, Lilley & Stevens, 2003; Dai et al., 2009; Dai et al., 2011; Sindhu et al., 2007; Niu et al., 2015). Most of the *COBRA* genes were involved in plant growth and development by regulating cellulose which eventually affected the formation of secondary walls. To further understand the *COBRA* gene family in Rosaceae, we identified all 87 members in six Rosaceae species and evaluated their phylogenetic relationships, hydrophobicity, gene structures, *cis*-regulatory elements, and tissue expression patterns. Our study will facilitate further functional studies of specific genes in the *COBRA* family.

## Materials and Methods

### Identification of *COBRA* genes in six Rosaceae species

In this work, the *Pyrus bretschneideri* genome was downloaded from GIGADB datasets (http://gigadb.org/dataset/100083), and five Rosaceae genomes (*Fragaria vesca*, *Rubus occidentalis*, *Prunus avium*, *Malus domestica*, *Prunus mume*) were obtained from the following website (https://www.rosaceae.org/). Arabidopsis *COBRA* gene family members amino acid sequences were used as the query sequence for BlastP (Protein BLAST: search protein databases using a protein query (nih.gov)) search (*E* = 0.001) from the local protein database. The SMART online software program (http://smart.embl-heidelberg.de/) was used to screen the genes (Letunic, Doerks & Bork, 2012). The protein sequences lacking a whole COBRA domain and redundant sequences were discarded. We used the ExPASY online website to predict the molecular weights, isoelectric points, and GRAVY values of COBRA protein (http://web.expasy.org/protparam/) (Artimo et al., 2012). Signal peptides were analyzed and predicted by SignalP 4.1 server (https://services.healthtech.dtu.dk/service.php?SignalP-5.0) software.

### Evolutionary analysis of *COBRA* gene family

Sequence alignment of all COBRA proteins was done using the ClustalW tool in MEGA 7.0 software. The phylogenetic tree was constructed with MEGA 7.0 software using the NJ (Neighbor-Joining) (bootstrap = 1,000). The sequences of *A. thaliana*, *O. sativa*, *Z. mays,* and *Populus* were obtained from the article (Roudier et al., 2002; Li et al., 2003; Sindhu et al., 2007; Zhang et al., 2010).

### *COBRA* gene structures and conserved motif prediction

The Gene Structure Display Server (http://gsds.gao-lab.org/) was used to investigate the gene structures (introns/exons) of the *COBRA* gene family members (Guo et al., 2007). The conserved sequences of the *COBRA* gene family were analyzed by MEME online (https://meme-suite.org/meme/) software (Bailey et al., 2015). The parameter settings are as follows: The number of identified motifs were 20 with Parameters for the conserved motif prediction were motif width greater than 6 and less than 200.

### Promoter analysis and *cis*-acting element analysis of *PbCOBL* genes

In this study, we found the 1,500 bp–2,000 bp upstream of the start codon in the pear genome database, which was the promoter sequence of the gene. The online software PlantCare (https://bioinformatics.psb.ugent.be/webtools/plantcare/html/) was used to examine cis-acting elements.

### Chromosomal location, gene duplication, and Ka/Ks ratio analysis

Mapinspect software was used to map the position of *COBRA* genes on chromosomes (Zhu et al., 2015; Su et al., 2019a). The determination of gene replication events of *COBRA* genes in 6 species mainly followed the following principles: First, the similarity of the two genes was greater than 80%. The distance between two genes on the same chromosome was more than 200 kb, which was tandem-duplicated genes. Two genes located on different chromosomes were defined as segmentally duplicated genes. Finally, DnaSP v5.0 software calculated non-synonymous (Ka) and synonymous substitution (Ks) values and performed a sliding window analysis. The parameter was set to window size 150 bp and step size 9 bp (Zhao et al., 2021).

### RNA extraction and qRT-PCR analysis

In this study, Dangshan su pear was used as the material, which growed in Dangshan County, Anhui Province. Samples of current-year flowers, buds, stems, mature leaves and fruits were obtained. The fruits were picked 15 days after pollination (DAP), 23 DAP 39 DAP, 47 DAP, 55 DAP, 63 DAP, 79 DAP, and 102 DAP. 39 DAP fruits were selected for different tissue expression analyses. We used the RNA extraction kit of Tiangen (Beijing, China) to extract RNA from different materials. Reverse transcription was performed using a PrimeScriptTM RT reagent kit with gDNA Eraser (TaKaRa, China). The qRT-PCR primers were designed using Beacon Designer 7 software (Table S1). The pear *Tubulin* gene (No. AB239680.1) was used as an internal reference (Wu et al., 2012). Each 20 µL qRT-PCR system included 10 µL of SYBR Premix Ex Taq TM II, 2 µL cDNA, 0.8 µL of Forward primer and Reverse primer, 6.4 µL of water. This study was conducted according to the procedures in the instruction manual, and three biological repetitions were run for each sample. The relative expression level of genes was calculated by the 2^−ΔΔCt^ method (Livak & Schmittgen, 2001).

## Results

### Identification, characterization analysis of *COBRA* genes

Using the amino acid sequences of *Arabidopsis COBRA* gene family members as probes, we identified 87 COBRA proteins in six Rosaceae species. Including 16 COBRA proteins (PbCOBL1-PbCOBL16) in *Pyrus bretschneideri*, 22 in *Malus domestica* (MdCOBL1-MdCOBL22), 13 in *Fragaria vesca* (FvCOBL1-FvCOBL13), 11 in *Prunus mume* (PmCOBL1-PmCOBL11), 13 in *Rubus occidentalis* (RoCOBL1-RoCOBL13) and 12 in *Prunus avium* (PaCOBL1-PaCOBL12). The detailed information (gene name, gene identifiers, amino acid number, signal peptide, molecular weight, theoretical isoelectric point, Grand average of hydropathicity and subdivided subgroup) of each *COBRA* was presented in Table 1 and Table S2. These results showed that the largest molecular weight among the six Rosaceae species was *MdCOBL12*, which was 131.89 kDa. The smallest was *PaCOBL4*, which was 12.59 kDa. Except for *MdCOBL17* and *PaCOBL3*, the grand average of hydropathicity of other genes were negative, indicating that most members of *COBRA* genes were hydrophilic proteins. In these Rosaceae species, the lowest pI value was 5.04 (*MdCOBL16*), whereas the highest pI value was 9.59 (*MdCOBL1*). Among the six Rosaceae species, about 74% of COBRA proteins contained signal peptides, and 26% of proteins did not contain a signal peptide.

**Table 1:** Basic Information of *COBRA* Gene in *Pyrus bretschneideri*.

**Gene name**	**Gene ID**	**AA**	**KD**	**pI**	**GRAVY**	**Signal peptide**	**Subdivided subgroup**
*PbCOBL1*	Pbr039918.1	435	49.18	8.46	−0.206	Yes	COBRA
*PbCOBL2*	Pbr033684.1	674	75.58	8.93	−0.318	No	COBL7
*PbCOBL3*	Pbr028526.1	335	38.19	6.64	−0.296	No	COBRA
*PbCOBL4*	Pbr026558.1	432	48.97	8.06	−0.339	Yes	COBRA
*PbCOBL5*	Pbr020181.1	633	70.78	9.00	−0.240	No	COBRA
*PbCOBL6*	Pbr020180.1	456	50.99	8.92	−0.105	Yes	COBRA
*PbCOBL7*	Pbr016608.1	457	51.64	9.10	−0.157	Yes	COBRA
*PbCOBL8*	Pbr011992.1	657	71.63	5.70	−0.124	Yes	COBL7
*PbCOBL9*	Pbr010999.1	311	35.17	6.30	−0.367	No	COBRA
*PbCOBL10*	Pbr009004.1	241	27.20	8.78	−0.425	No	COBRA
*PbCOBL11*	Pbr008592.1	610	66.55	5.07	−0.070	No	COBRA
*PbCOBL12*	Pbr007186.1	456	50.89	8.92	−0.084	Yes	COBRA
*PbCOBL13*	Pbr007185.1	445	49.81	8.73	−0.287	Yes	COBRA
*PbCOBL14*	Pbr007184.1	423	46.82	9.00	−0.203	Yes	COBRA
*PbCOBL15*	Pbr004198.1	674	75.60	8.99	−0.337	Yes	COBL7
*PbCOBL16*	Pbr001136.1	692	75.24	5.96	−0.071	Yes	COBL7

### Phylogenetic and hydrophobic analysis of *COBRA* genes

**Figure 1:**
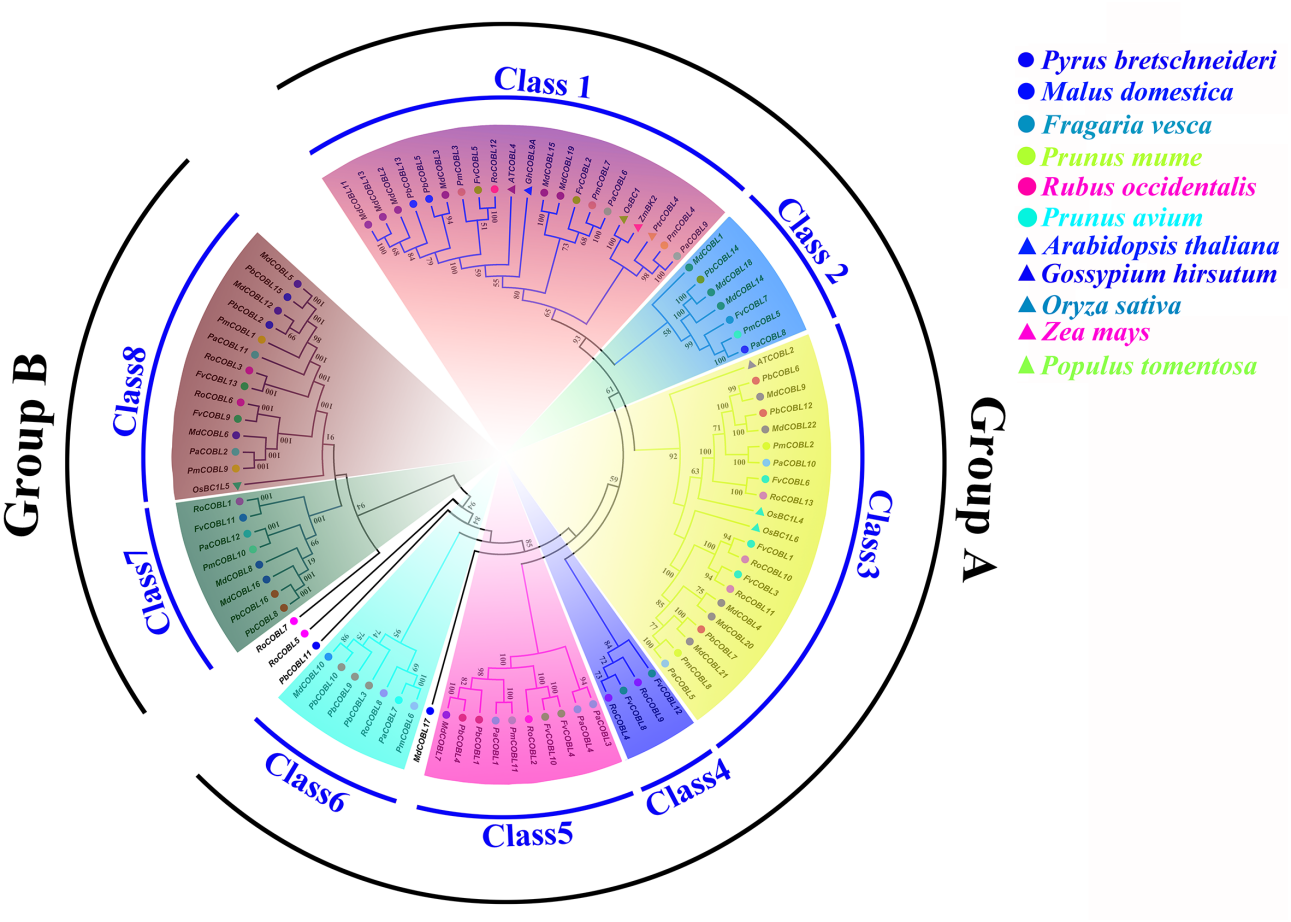
Phylogenetic relationships and subfamily designations in COBRA proteins from *Pyrus bretschneideri*, *Fragaria vesca*, *Prunus mume*, *Rubus occidentalis*, *Prunus avium*, *Malus domestica*, *Arabidopsis thaliana*, *Oryza sativa*, *Zea mays*, *Gossypium hirsutum*, *Populus tomemtosa*.

Phylogenetic analysis showed that the 87 *COBRA* genes were classified into two subclasses (Group A and Group B), similar to the *Arabidopsis* (Fig. 1, Table S5). Group A was structurally similar to *AtCOBRA*, and Group B had a higher similarity to *AtCOBL7*. We divided Group A into Class1–Class6 and Group B into Class7–Class8. *MdCOBL17*, *RoCOBL7*, *RoCOBL5*, and *PbCOBL11* were independent branches in the evolutionary tree, and no genes related to them have been identified. Most *PbCOBL* genes were more tightly grouped with *MdCOBLs*. In Class1, *AtCOBL4* and *GhCOBL9A* were clustered with *MdCOBL11*, *MdCOBL13*, *MdCOBL2*, *MdCOBL3*, *PbCOBL13*, *PbCOBL5*, *PmCOBL3*, *FvCOBL5*, *RoCOBL12*. *OsBC1*, *ZmBK2*, *PtrCOBL4* with *PmCOBL4*, *PaCOBL9* in one branch. *AtCOBL2*, *OsBC1L4*, and *OsBC1L6* appeared in Class3, and *OsBC1L5* was alone in Class8.

**Figure 2:**
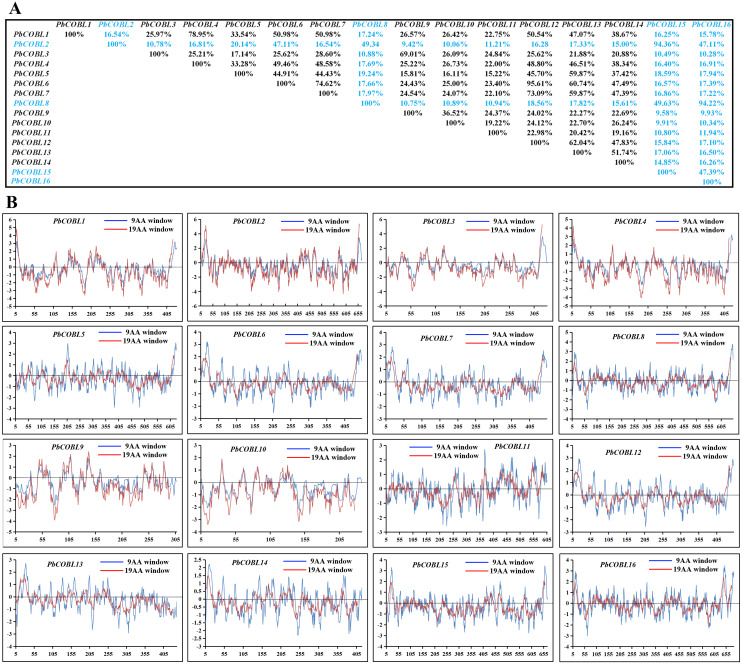
Characteristics of COBRA proteins in *Pyrus bretschneideri*. (A) *COBRA* member similarity comparison. (B) Comparison of hydrophobicity of *COBRA* members.

We compared the similarity of COBRA proteins of six Rosaceae species (Fig. 2, Figs. S1–S5). In *Pyrus bretschneideri*, comparisons among the Group B subgroup genes showed identity in the range of 47.11% to 94.36%. Within the Group A subgroup, the proteins are 15.22% to 95.61% identical. Protein similarity between the two subgroups ranged from 9.58% to 47.11% (Fig. 2). In *Prunus mume*, the similarities among the three members of the Group B subfamily were 53.3% for *PmCOBL1*-*PmCOBL9*, 50.66% for *PmCOBL1*-*PmCOBL10*, and 49.32% for *PmCOBL9*-*PmCOBL10*, respectively. The similarity between Group A and Group B subfamilies ranged from 12.28% to 19.25%, and the similarity between Group A subfamily members ranged from 15.27% to 76.75% (Fig. S1). In *Rubus occidentalis*, the similarity with other members of *RoCOBL7* was low because *RoCOBL7* was a separate branch in the evolutionary tree. The similarities between the three members of the Group B subclade, *RoCOBL1*, *RoCOBL3*, and *RoCOBL6*, were 49.78%, 56.63%, and 50.6%, respectively. The protein similarity between the two members of the two subclades ranged from 5.86% to 16.59%. The similarity of Group A subclade members ranged from 7.63%–85.40% (Fig. S2). In *Fragaria vesca*, Group B, Group A and two subclades individual member similarities ranged from 47.96% to 56.29%, 15.4%–93.21%, and 7.49%–19.45%, respectively (Fig. S3). In *Prunus avium*, the similarity of three members *PaCOBL2*, *PaCOBL11*, and *PaCOBL12* was 23.45%, 41.84%, and 28.14%, respectively. The results of the comparison between subgroups were 8.05%–18.39%. Protein similarity among Group A members ranged from 6.64%–72.44% (Fig. S4). In *Malus domestica*, Group B, Group A and two subclades, individual member similarities ranged from 25.68%–79.05%, 5.98%–94.04%, 5.98–94.04% (Fig. S5).

We studied their hydrophobicity to determine if the 87 proteins were likely to have a GPI anchor similar to COBRA. We performed the hydrophobic analysis of 87 *COBRA* genes from six Rosaceae species. As shown in Fig. 2 and Figs. S1–S5, most amino acids showed a similar trend, with the middle part hydrophilic and the ends hydrophobic. The GPI modification sites and potential w-cleavage sites of 87 proteins were predicted using big-PI. Among the 87 proteins, the program found 34 significant potentials for GPI modification using the default parameters and proposed a possible GPI addition for the remaining three proteins (*FvCOBL1*, *PbCOBL1*, *PbCOBL4*, *PmCOBL5*, *PmCOBL11*, *RoCOBL2*, *PaCOBL1*). All proteins have potential w-cleavage sites.

**Figure 3:**
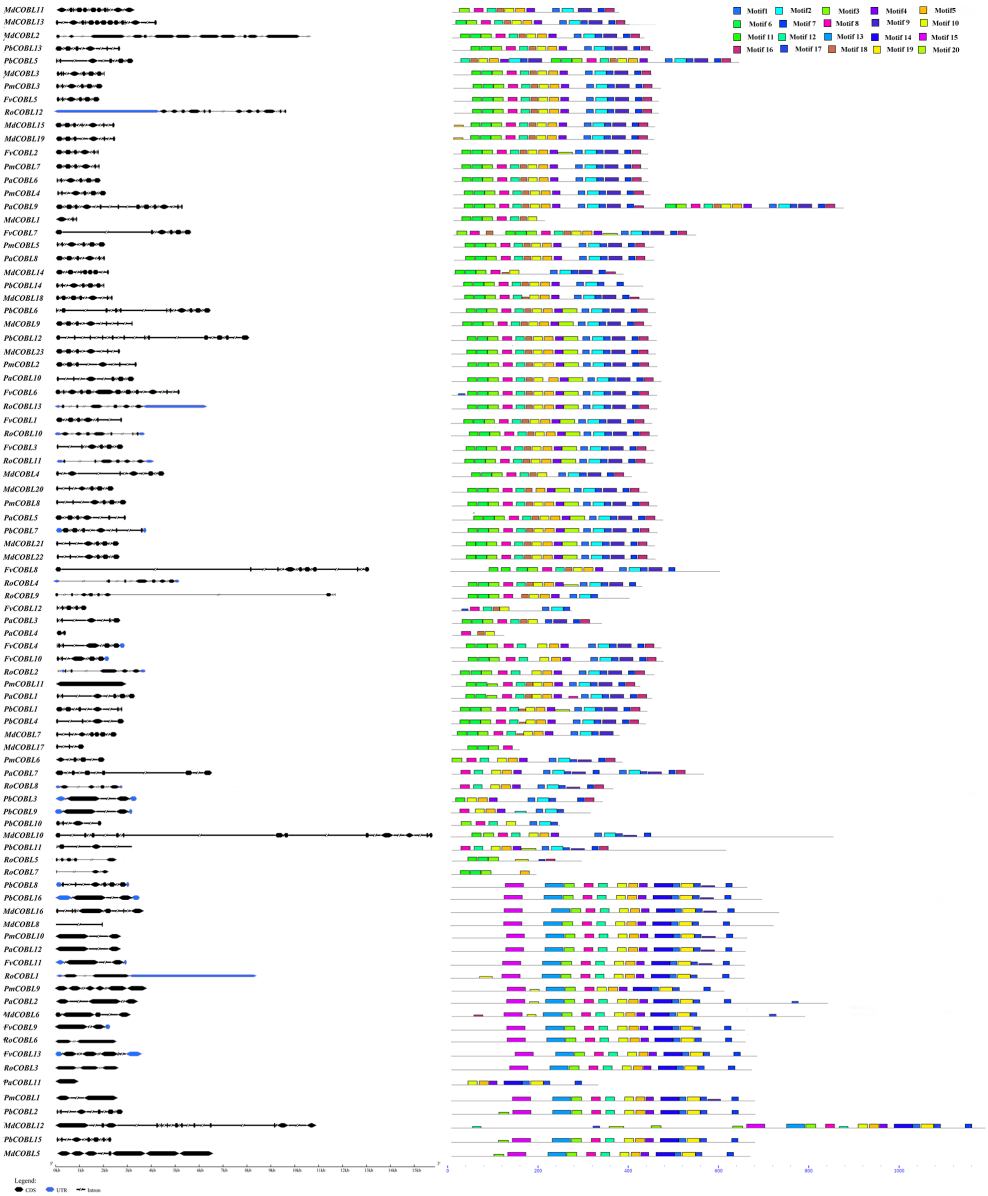
Predicted *Pyrus bretschneideri*, *Fragaria vesca*, *Prunus mume*, *Rubus occidentalis*, *Malus domestica* and *Prunus avium* COBRA protein conserved motifs and exon-intron structures. (A) Gene structures of the *COBRA* genes. (B) Distribution of MEME motifs in *COBRA* genes. (C) The color and corresponding number of each motif box.

### Structural and conserved motif analysis of COBRA proteins

To gain a comprehensive understanding of the diversity of *COBRA* genes in the six Rosaceae families, we performed gene structure and conserved sequence analysis of 87 genes (Fig. 3). Group A had 66 members with the number of exons ranging from one to 12, of which 30 members had six exons, five members contained 12 exons, and one member contained 13 exons. Group B had 21 members, of which eight members contained only two exons, three members contained three exons, two members contained one exon, and one member contained four exons. In addition, *PbCOBL8*, *PmCOBL9*, *PbCOBL2* contained six exons. *MdCOBL16*, *PbCOBL15*, *MdCOBL5* contained seven exons and *MdCOBL12* contained 13 exons. We performed a conserved structure analysis of 87 genes using MEME online software. We found that motif 8, motif 6, and motif 10 were relatively conservative, motif 15 and motif 13, and motif 14, were specific to Group B members (except for *PaCOBL11*), and motif 2 was specific to Group A.

**Figure 4:**
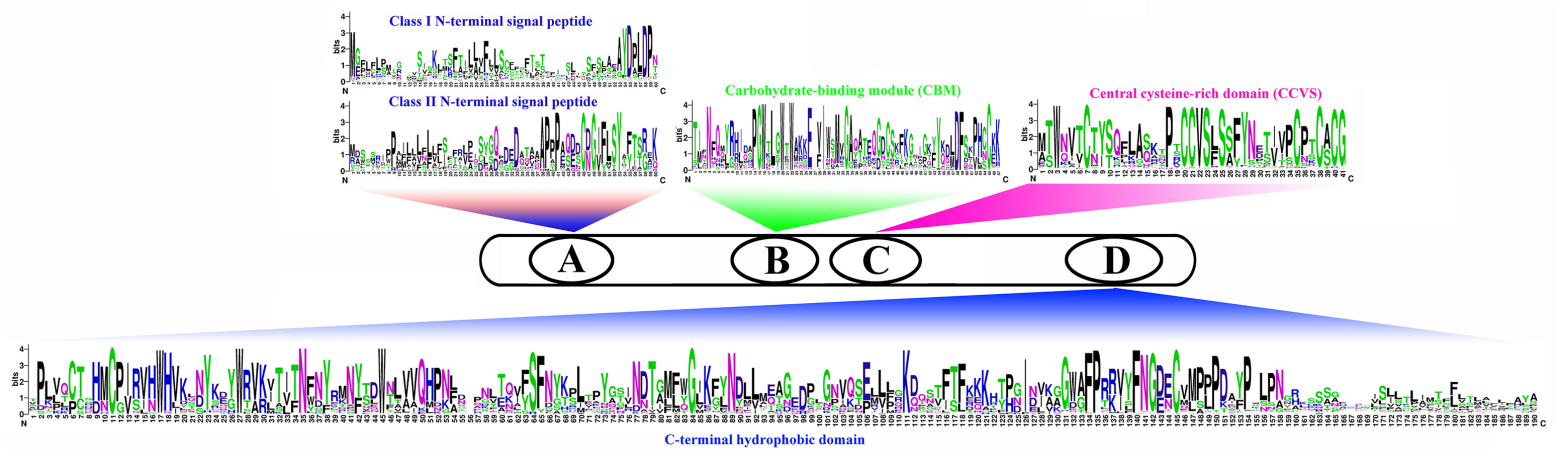
Conserved COBRA domain composition. All 87 *COBRA* genes had a characteristic ID domain. Alignment analysis of the 87 *COBRA* gene sequences using the ClustalW tool in MEGA 7.0 software. These domain diagrams were plotted using the online WebLogo tool.

We compared the sequences of six species (Fig. 4, Figs. S6–S11). Four conserved structural domains were identified in 87 genes, N-terminal signal peptide, Carbohydrate-binding module (CBM), central cysteine-rich domain(CCVS), and C-terminal hydrophobic domain, respectively. Sequence comparison revealed that GroupB members generally had more amino acids than Group A, and the N-terminal signal peptide was different in the two subgroups. Analysis of the conserved regions of the six species *COBRA* members revealed that the central cysteine-rich domain (CCVS) was the relatively conserved region in both subclades, and almost all members contained the CCVS structural domain. The Central cysteine-rich domain contained a consensus N-glycosylation site, this site was mainly associated with post-translational modifications of GPI-anchored proteins and more generally with extracellular proteins. There was also an N-glycosylation site in the C-terminal hydrophobic domain. N-terminal signal peptide and C-terminal hydrophobic domain played an important role in function. However, the sequence comparison results showed that the similarity between N-terminal signal peptide and C-terminal hydrophobic domain in each subgroup was not high, suggesting that there was no solid selective pressure on these areas as long as their hydrophobic nature was considered.

### Chromosomal location and duplication events of *COBRA* family genes in six Rosaceae

**Figure 5:**
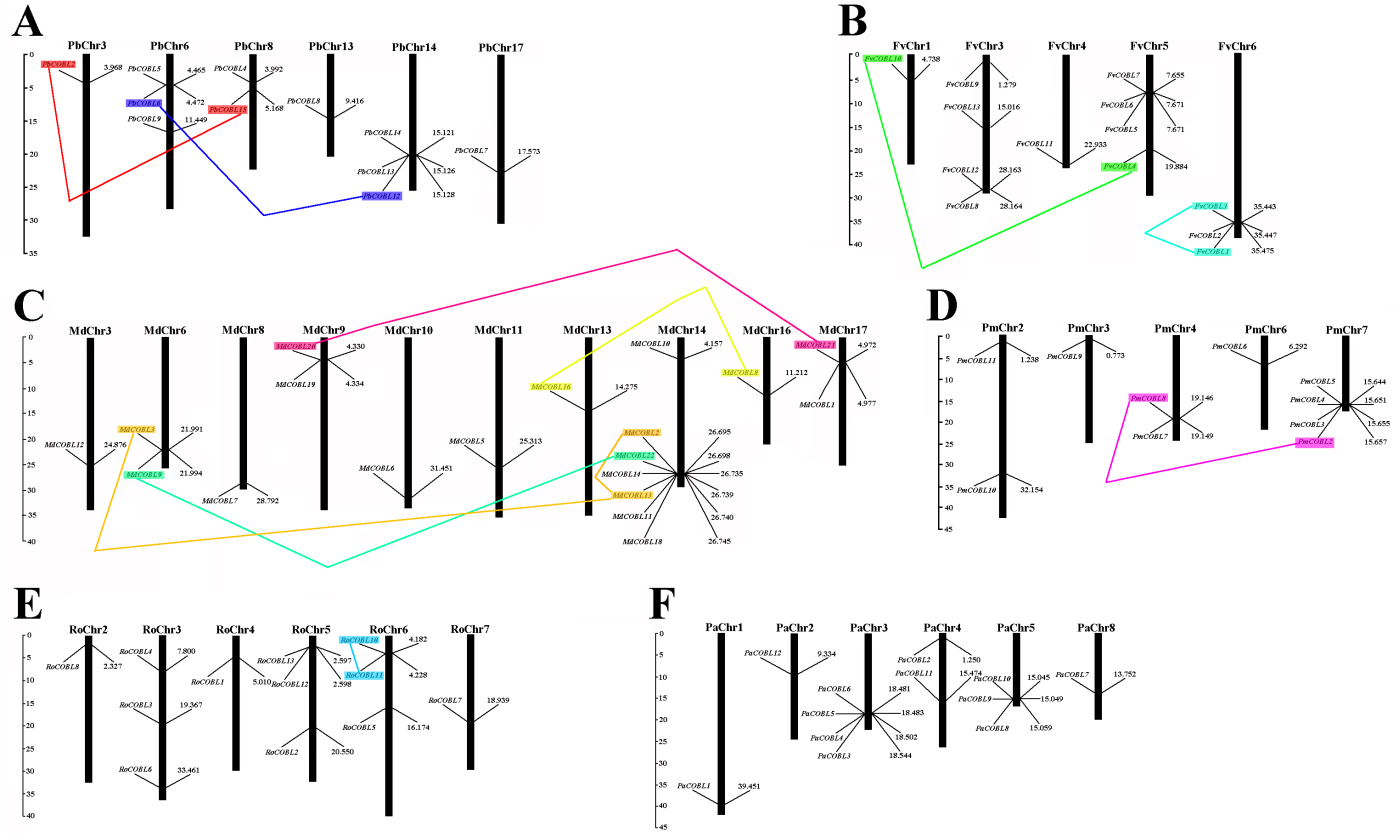
Chromosomal locations of six Rosaceae species. Chromosomal locations of COBRA genes in *Pyrus bretschneideri* (A), *Fragaria vesca* (B), *Malus domestica* (C), *Prunus mume* (D), *Rubus occidentalis* (E), and *Prunus avium* (F). Duplicated gene pairs are connected with colored lines.

Based on the genome-wide data of pear, strawberry, black raspberry, sweet cherry, japanese apricot, and apple, all *COBRA* genes exact chromosomal physical localization was determined, as shown in Fig. 5. In pear, 11 *PbCOBL* genes were distributed on six chromosomes (Chr3, Chr6, Chr8, Chr13, Chr14, Chr17), and five genes were not localized on any chromosome. In strawberry, 13 genes were located on five chromosomes (except Chr2 and Chr7). In Japanese apricot, four genes were distributed on chromosomes 7; two genes on chromosomes 2 and 4; and one gene on chromosomes 3 and 6, respectively. In black raspberry, there were no genes on chromosome 1; three genes on chromosomes 3, 5, and 6, and one gene on chromosomes 2, 4 and 7. In apple, 18 genes were distributed on chromosomes 3, 6, 8, 9, 10, 11, 13, 14, 16 and 17 (except Chr1, 2, 4, 5, 7, 12 and 15). In sweet cherry, 12 genes were distributed on six chromosomes (except Chr6 and 7), of which there were four genes on chromosome 3, only one gene on chromosome 1, 2 and 8, two genes on chromosome 4 and three genes on chromosome 5.

**Figure 6:**
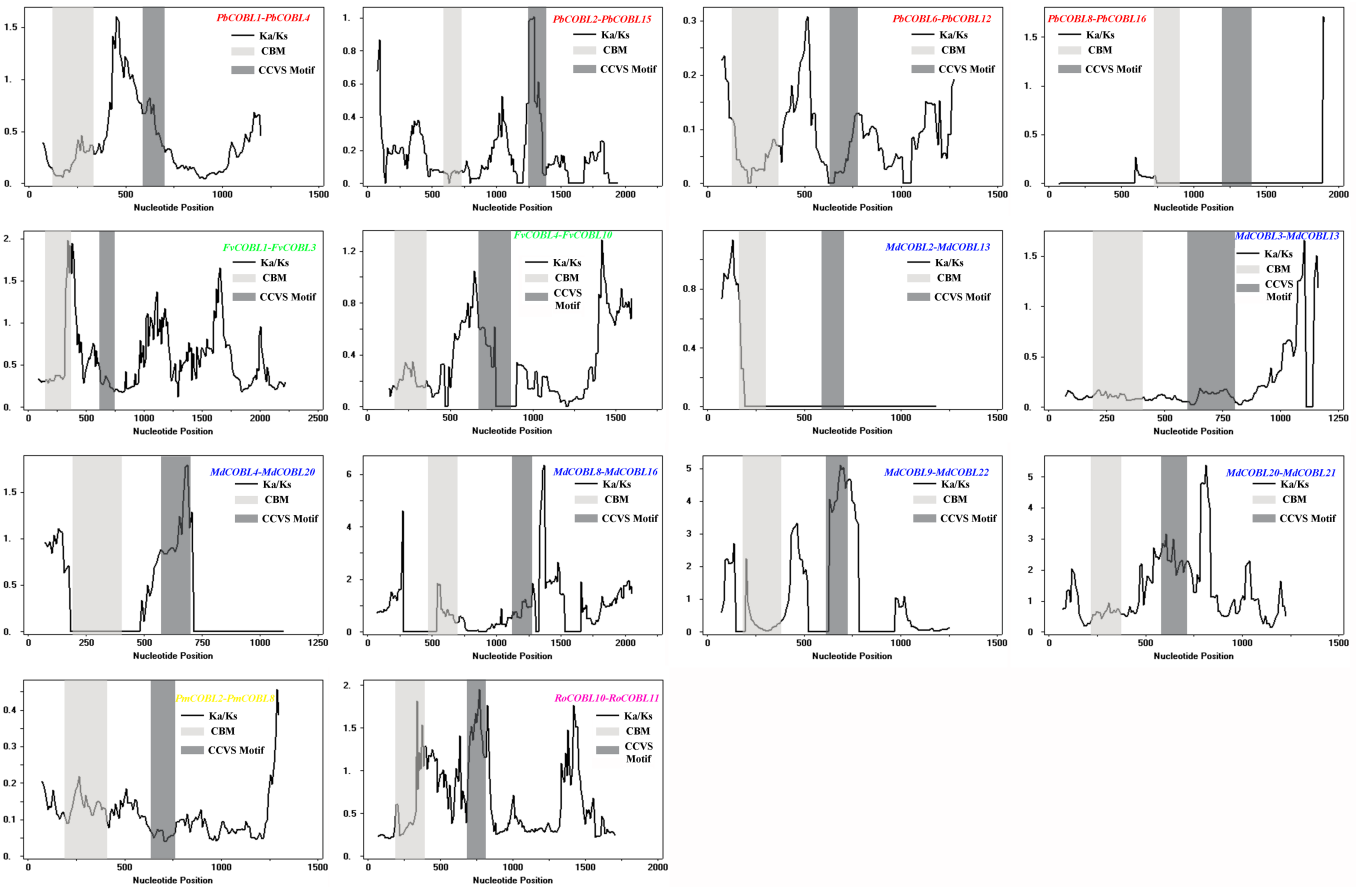
Sliding window plots of duplicated *COBRA* genes.

To understand the role of drivers of gene duplication in the evolutionary process, we calculated Ka, Ks, and Ka/Ks ratios of duplicated gene pairs in six Rosaceae species (Fig. 5, Table S3). Ka/Ks = 1 is the cut-off value that indicates neutral selection, Ka/Ks < 1 represents negative selection and Ka/Ks > 1 represents a positive selection. All 14 duplicated gene pairs were identified in pear, strawberry, apple, Japanese aprico, and black raspberry. There were four duplicated gene pairs in pear, two pairs in strawberry, six pairs in apple, one pair in plum and 1 pair in black raspberry. Among the 14 replicated gene pairs, only *MdCOBL3*-*MdCOBL13* and *RoCOBL10*-*RoCOBL11* Ka/Ks > 1 were 1.272 and 1.141, respectively, and the remaining replicated gene pairs had Ka/Ks < 1. Among the 14 replication gene pairs, 12 gene pairs experienced segmental duplication, and only two pairs of genes (*RoCOBL10*-*RoCOBL11*, *FvCOBL1*-*FvCOBL3*) experienced tandem duplication. To gain a comprehensive understanding of the selection pressure on *COBRA* genes, we performed a sliding window analysis (Fig. 6), which showed that in 14 pairs of gene replication events, most of the Ka/Ks loci were less than 1, and only very few loci had Ka/Ks values greater than 1.

### Analysis of *cis*-acting elements of *COBRA* gene family promoter in *Pyrus bretschneideri*

**Figure 7:**
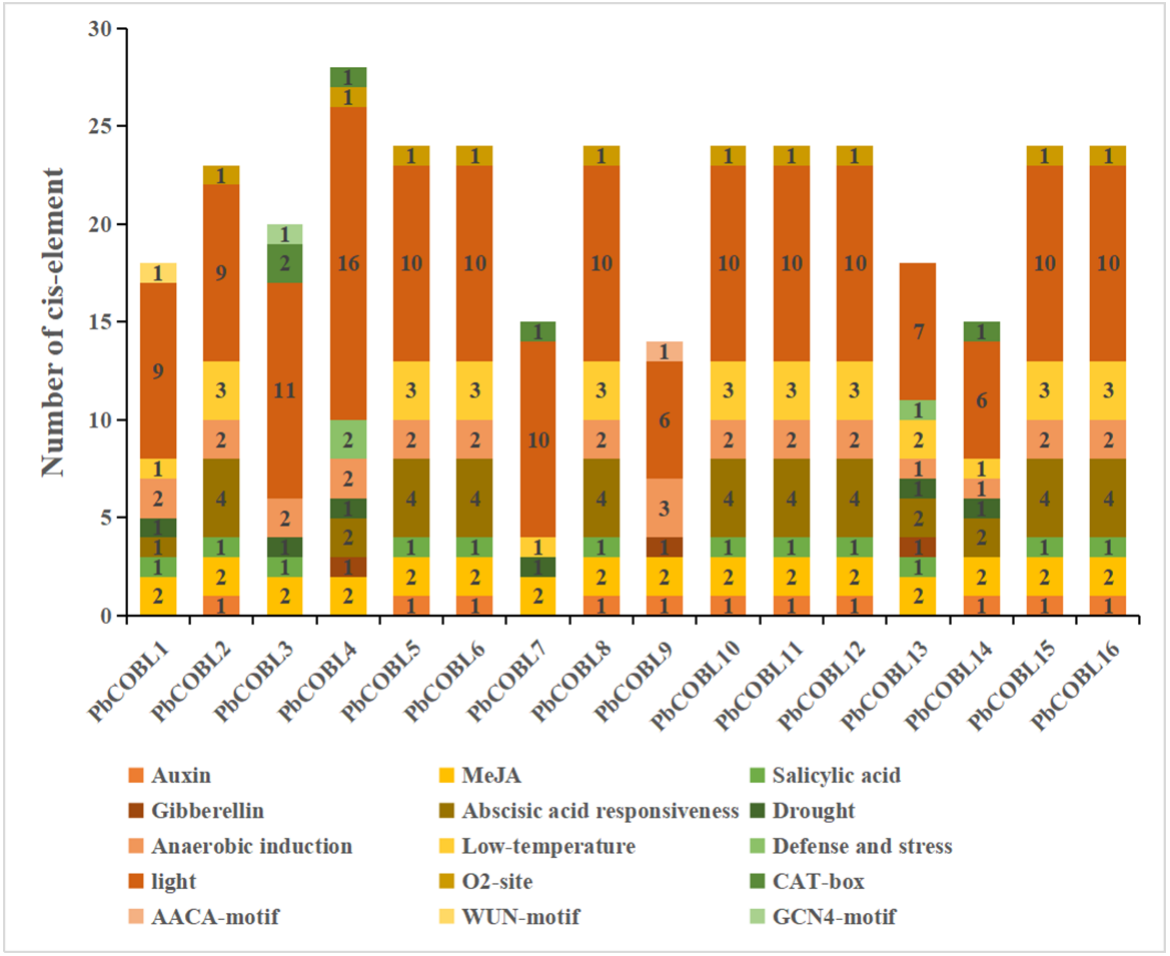
Promoter *cis*-elements of the 16 *PbCOBLs*.

In order to study the specific expression of the *COBRA* gene, We used the plant care database to study *cis*-acting elements of promoters of 16 *COBRA* genes in *Pyrus bretschneideri* (Fig. 7, Table S4). The promoter of *COBRA* genes contained many *cis*-acting elements related to hormones, including responses to Auxin-responsive element (TGA-box, AuxRE), MeJA-responsiveness (CGTCA-motif, TGACG-motif), Salicylic acid responsiveness (TCA-element), Gibberellin-responsiveness, Abscisic acid responsiveness. CGTCA-motif and GACG-motif *cis*-acting elements were identified in all 16 genes. A total of 11 c*is*-acting elements associated with auxin were found in 11 members. Among them, AuxRE was only present in *PbCOBL14*, TGA-box was not contained in *PbCOBL1,3,4,7,13,14*, and all other genes were contained. TCA-element, which are *cis*-acting elements involved in salicylic acid responsiveness, were identified in *PbCOBL1, 2, 3, 5, 6, 8, 10, 11, 12, 13, 15* and *16*. Gibberellin-responsiveness (P-box, GARE-motif, TATC-box) was identified only in *PbCOBL4,9* and *13*. Abscisic acid responsiveness (ABRE) were more prominentin numbers, 43 ABRE *cis*-acting elements were identified in 13 genes, and only *PbCOBL3,7,9* was not identified with ABRE.

It is related to plant growth and development, including participating in light response elements (G-box, GT1 motif, GATA motif, Sp1, Box 4, TCT-motif, AE-box, Lamp element, CHS-cma1a, GA motif, I-box, 3-af1 binding site, ACE, TCCC motif) were identified 154 times in 16 genes. The *cis*-acting regulatory element O2-site involved in the regulation of maize alcohol-soluble protein metabolism was identified in 10 members (*PbCOBL2,4,5,6,8,10,11,12,15* and *16*). Meristem expression (CAT-box) was identified in *PbCOBL3,4,7* and*14*. Endosperm specific negative expression (AACA motif). Wound-responsive element (WUN-motif) and Endosperm expression (GCN4-motif) were only identified in *PbCOBL9*. *cis*-acting elements associated with biotic and abiotic stress responses were also identified in *COBRA* genes. Six *PbCOBL* genes (*PbCOBL1, 3, 4, 7, 13,* and *14*) had MBS, and two members (*PbCOBL3,14*) contained TC-rich repeats. Anaerobic induction (ARE) was identified in 15 genes (Except *PbCOBL7*). A total of 32 identifications in 13 members of the low-temperature responsiveness (LTR). *COBRA* family promoters were mostly engaged in hormone response and light response processes. Plant growth and development are facilitated by the reaction to various hormones and light responses.

### Expression characteristics of Chinese white pear *COBRA* genes

**Figure 8:**
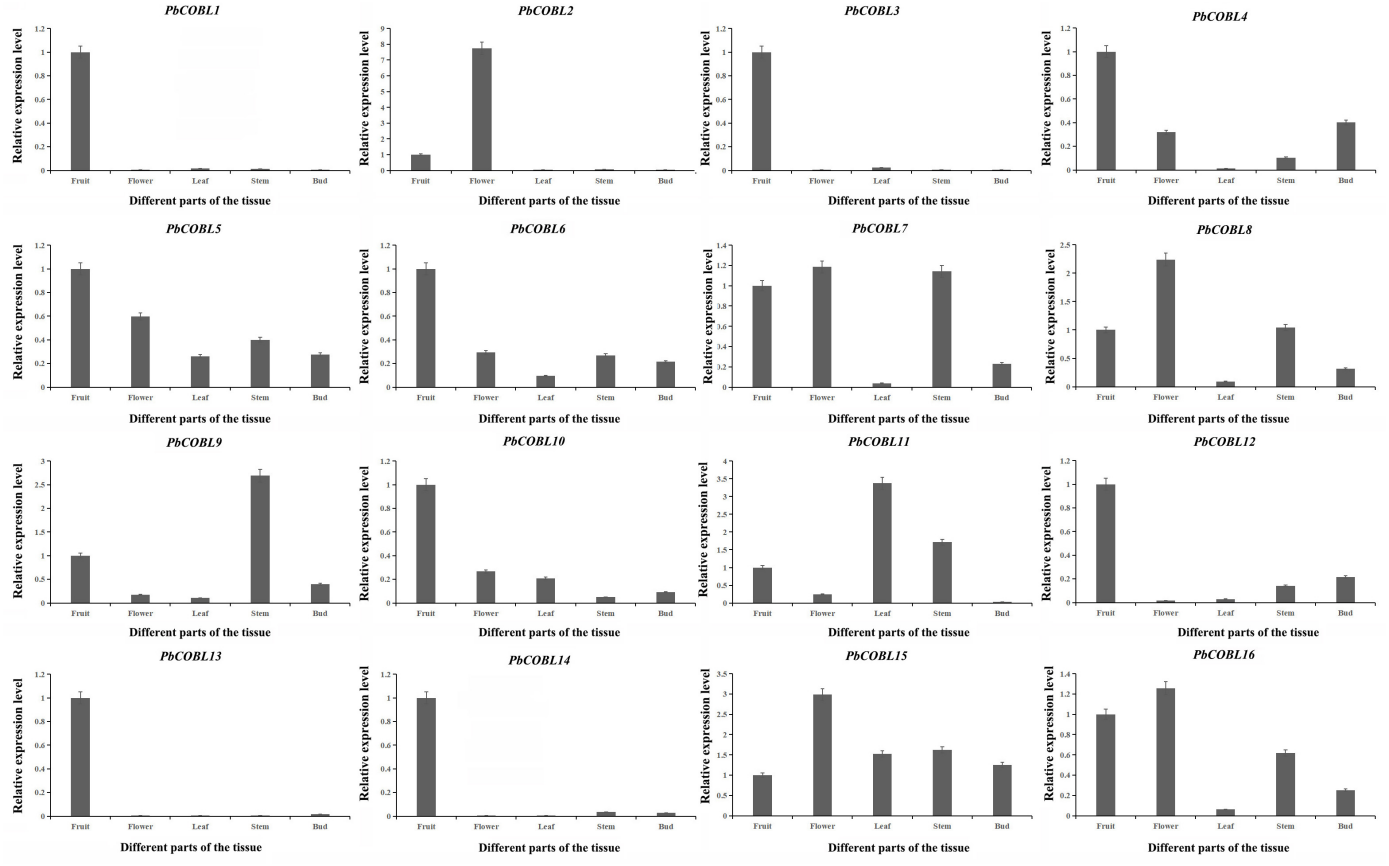
Expression patterns of *COBRA* genes of Chinese white pear in different organs.

To gain a deeper understanding of the *COBRA* gene family, we performed expression pattern analysis in the stems, leaves, fruits, flowers and buds of Dangshan su pear (Fig. 8). The results showed that *PbCOBL1,3,10,12,13,14* were highly expressed only in fruits and almost not in other tissues. *PbCOBL2* was highly expressed in flowers. *PbCOBL5,15* were expressed at relatively high levels in several tissues. *PbCOBL9* was highly expressed in stems. *PbCOBL4,6,7,8,16* were expressed at low levels in leaves and at high levels in other tissues.

**Figure 9:**
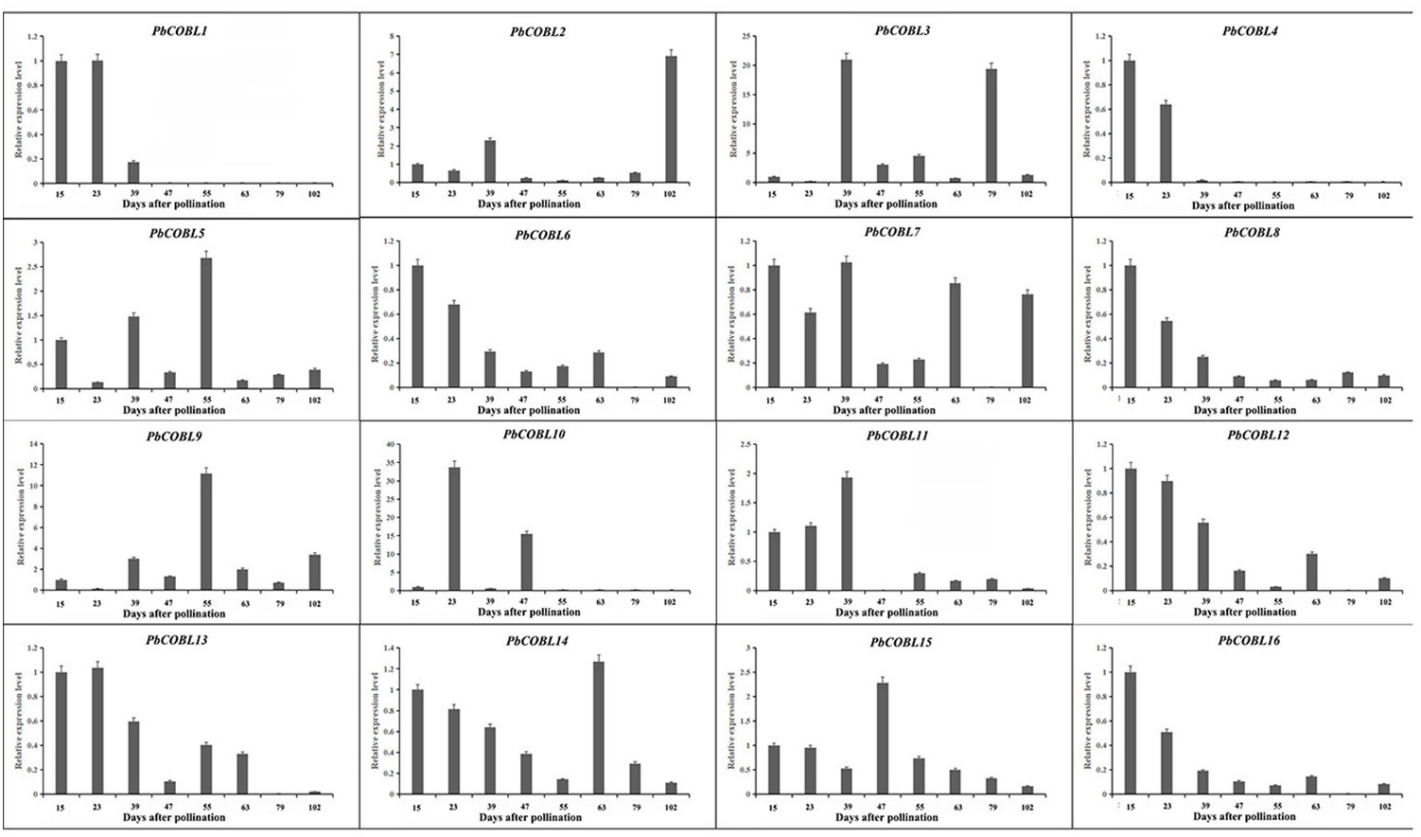
Expression patterns of *COBRA* genes of Chinese white pear in fruit at different developmental stages.

We examined the expression pattern of the *COBRA* gene family in eight periods of fruit development of Dangshan su pear (Fig. 9). As shown in the figure, *PbCOBL1 and PbCOBL4* were substantially expressed at 15 and 23 DAP and non significant expression were found on remaining time interval. *PbCOBL6,8,11,12,16,13* showed similar expression patterns, with higher expression in early fruit development (15 DAP, 23 DAP, 39 DAP) and a decreasing trend in late fruit development. *PbCOBL10* expression showed two peaks at 23 DAP and 47 DAP during eight periods of fruit development, and *PbCOBL2* showed a similar expression pattern with *PbCOBL10*, with two peaks at 39 DAP and 102 DAP. *PbCOBL7* was barely expressed in 79 DAP. *PbCOBL5* and *PbCOBL9* showed a peak at 55 DAP, with lower expression in other periods. *PbCOBL14,15* were expressed at each period of fruit development, with *PbCOBL14* highly expressed at early fruit development (15,23,39 DAP) and 63 DAP, *PbCOBL15* peaking at 47 DAP, and low expression at other periods. *PbCOBL3* was mainly highly expressed in 39 DAP and 79 DAP.

## Discussion

COBRA, a glycosyl-phosphatidyl inositol-anchored protein (GPI), generally had an N-terminal signal peptide, Carbohydrate-binding module (CBM), central cysteine-rich domain (CCVS), and a hydrophobic C-terminal hydrophobic domain. Among them, CBM was a functional structural domain with strong binding to crystalline cellulose. The CCVS region was mainly involved in forming disulfide bonds or metal ion binding with cysteine-rich features (Roudier et al., 2002; Liu et al., 2013; Niu et al., 2018). Previous studies found that the *COBR* family was involved in cell wall biosynthesis-related to plant roots, stems, leaves and fruit ripened (Dai et al., 2009; Brown et al., 2005; Persson et al., 2007).

In this study, a total of 87 *COBRA* genes were identified in six Rosaceae species. The pI values of 87 members ranged from 5.04 (*MdCOBL16*) to 9.59 (*MdCOBL1*). The proteins encoded by the *COBRA* gene include acidic protein and basic protein. Except for *MdCOBLl17* and *PaCOBL3*, the other 85 genes were negative, indicating that most COBRA proteins were hydrophilic proteins (Table S2). Among Rosaceae species, pear had the second-highest number of *COBRA* members after apples, a phenomenon that might be related to whole-genome duplication (WGD). Studies have shown that WGD and chromosome rearrangements were accompanied by chromosome doubling, altered gene sequences, and also extensive gene loss (Tang et al., 2008). The common ancestor of Rosaceae has nine chromosomes. Apple and pear experienced WGD twice in 130 million years ago (Mya) and 30–45 Mya, while the other four species only experienced WGD of 130 million years ago (Mya) (Shulaev et al., 2011; Wu et al., 2013). The long process of biological evolution was accompanied by chromosome doubling, breaking, and rearrangement, resulting in 17 chromosomes for pears and apples, eight for japanese apricot and sweet cherry, and seven for strawberry and black raspberry. During the evolution of the *COBRA* gene family, the number of *COBRA* genes in pears might be less than that of apples due to the loss of members. For phylogenetic analysis, the 87 *COBRA* members were divided into two subclades which was further subdivided Subclade A (Class1–Class6) and subclade B (Class7–Class8). Among them, *MdCOBL17*, *RoCOBL7*, *RoCOBL5*, and *PbCOBL11* were independent branches in the evolutionary tree, and no genes related to them were identified. Interestingly, the *COBRA* genes of pear and apple were present in Class1–Class8, at least one gene, and this phenomenon might be due to the involvement of pear and apple in the second WGD.

Gene function was strongly related to gene structure and conserved sequence, with similar conserved structural domains in the same family and higher similarity in the same subclade, implying that members of the same subclade may have similar functions to each other. For example, the *FvCOBL5* and *PmCOBL3* genes were structurally similar (similar number and length of exons). In terms of gene structure, Group A members had a high number of exons, and analysis of conserved regions revealed that the same subgroup had approximately the same conserved protein regions, with differences between different subgroups. A previous study found that *COBL7* differs from *COBRA* in the N-terminal signal peptide region, and a specific 170 amino acid sequence of the *COBL7* subfamily was found in several species, which overlaps with the *COBRA* subfamily N-terminal signal peptide after 170 amino acids (Roudier et al., 2002). This difference might result in differences in the function of the two subgroups. A similar phenomenon was found in 87 *COBRA* genes of six Rosaceae (Figs. S6–S11). Gene duplication events were often followed by the differentiation of gene functions, such as the creation of new functions and the loss of old ones. Thus, gene duplication events were the driving force of biological evolution, allowing organisms to become more adapted to the diversity of their environment (Chao et al., 2017; Tang et al., 2016). Previous studies found that gene replication events have been found in multiple gene families, such as the *PKS* gene family in cotton and the *MADS*-box gene family in pear (Su et al., 2017; Meng et al., 2019). In current study, 14 pairs of replication gene pairs were identified, including four pairs of pear, six pairs of apple, one pair of strawberry, one pair of Japanese aprico, and one pair of black raspberry. Among them, *FvCOBL1*-*FvCOBL3* and *RoCOBL10*-*RoCOBL11* underwent tandem duplication, and the remaining 12 gene pairs underwent segmental duplication. Apples and pears had significantly more replicate gene pairs than the other four Rosaceae species, probably because pears and apples experienced two WGDs while the other four Rosaceae species experienced one WGD (Wu et al., 2013; Zhang et al., 2012). We calculated the Ka, Ks and Ka/Ks values for 14 replicated gene pairs. We found that the Ka/Ks values of *MdCOBL3*-*MdCOBL13* and *RoCOBL10*-*RoCOBL11* were greater than 1, indicating that these gene pairs underwent rapid evolutionary diversification after a duplication event during evolution. The Ka/Ks of other genes is less than 1, implying that these gene pairs have been experiencing a markedly purifying selection during evolution (Table S3).

The overall analysis of expression profiles in different tissues will contribute to studying the tissue-specific and dynamic expression of *COBRA* genes in pear. The high expression of *PbCOBL15*, *PbCOBL5* in different tissues suggested that *PbCOBL15*, *PbCOBL5* played an important role in the growth and development of Dangshan su pear (Fig. 9). A previous study found that *COBRA* gene family was involved in the synthesis of secondary wall cellulose (Dai et al., 2011; Liu et al., 2013; Brown et al., 2005). In our study, we found that *PbCOBL1,3,12,13,14* were only highly expressed in fruits and hardly expressed in other tissues. It was also found that the *cis*-acting elements of their promoter contained many hormone response elements and light response elements. Interestingly, only the *PbCOBL13* promoter contained the gibberellin responsiveness element, but not in the other three genes, which might be the reason for the functional difference (Fig. 7).

Previous studies found that the development of stone cells in Dangshan su pear fruit increased initially and then decreased, starting from 7 DAP and reaching the peak at 55 DAP (Zhang et al., 2017; Su et al., 2019b). The expression patterns of these 16 *PbCOBLs* at eight developmental stages of fruit showed that the expression pattern of any one gene was not consistent with the trends obtained for the Dangshan su pear fruit stone cell. However, there were two special genes, *PbCOBL12* and *PbCOBL13*, which were highly expressed in the early stage of fruit development (15 DAP, 23 DAP, and 39 DAP). The 15 DAP-39 DAP is a process of massive accumulation of lithocytes accompanied by high expression of *PbCOBL12* and *PbCOBL13* (Fig. 8). According to the phylogenetic results, *PbCOBL13* clustered with *AtCOBL4* as a branch, and *PbCOBL12* was in Class3 with *AtCOBL2* (Fig. 1). Previous studies illustrated that *AtCOBL4* and *AtCOBL2* ultimately affect secondary wall formation by regulating the expression of cellulose synthase (Ben-Tov et al., 2015; Brown et al., 2005). Protein three-dimensional structures prediction showed that *PbCOBL12* was similar to *AtCOBL2* and *PbCOBL13* was similar to *AtCOBL4* (Fig. S12). We speculate that *PbCOBL12*, and *PbCOBL13* are mainly expressed in fruit and have similar functions to *AtCOBL2* and *AtCOBL4* in regulating SCW formation in pear fruit cells by regulating the expression of the genes encoding key cellulose synthesis enzymes.

## Conclusions

In this study, 87 *COBRA* genes were identified in six Rosaceae species. We analyzed the evolutionary relationship between *COBRA* in six species using evolutionary analysis, hydrophobicity analysis, gene structure, and conservative sequence analysis, *cis*-acting element analysis, gene duplication and slide window analysis, spatiospatiotemporal expression pattern analysis, and screened *PbCOBL12* and *PbCOBL13* as key genes regulating secondary wall during Dangshan su pear fruit development.

## Supplemental Information

10.7717/peerj.13723/supp-1 Figure S1 Characteristics of cobra family proteins in *Prunus mume*. A: *COBRA* member similarity comparison. B: Comparison of hydrophobicity of *COBRA* members

10.7717/peerj.13723/supp-2 Figure S2 Characteristics of cobra family proteins in *Rubus occidentalis*. A: *COBRA* member similarity comparison. B: Comparison of hydrophobicity of *COBRA* members

10.7717/peerj.13723/supp-3 Figure S3 Characteristics of cobra family proteins in *Fragaria vesca.* A: *COBRA* member similarity comparison. B: Comparison of hydrophobicity of *COBRA* members

10.7717/peerj.13723/supp-4 Figure S4 Characteristics of cobra family proteins in *Prunus avium*. A: *COBRA* member similarity comparison. B: Comparison of hydrophobicity of *COBRA* members

10.7717/peerj.13723/supp-5 Figure S5 Characteristics of cobra family proteins in *Malus domestica* A: *COBRA* member similarity comparison. B: Comparison of hydrophobicity of *COBRA* members

10.7717/peerj.13723/supp-6 Figure S6 *Fragaria vesca* COBRA protein sequence alignment

10.7717/peerj.13723/supp-7 Figure S7 *Pyrus bretschneideri* COBRA protein sequence alignment

10.7717/peerj.13723/supp-8 Figure S8 *Prunus mume* COBRA protein sequence alignment

10.7717/peerj.13723/supp-9 Figure S9 *Prunus avium* COBRA protein sequence alignment

10.7717/peerj.13723/supp-10 Figure S10 *Malus domestica* COBRA protein sequence alignment

10.7717/peerj.13723/supp-11 Figure S11 *Rubus occidentalis* COBRA protein sequence alignment

10.7717/peerj.13723/supp-12 Figure S12 Predicted three-dimensional structures of *Pb COBRL1 3*, *PbCOBL12*, *AtCOBL 2*, *AtCOBL4*

10.7717/peerj.13723/supp-13 Supplemental Information 13 Primers used in qRT-PCR

10.7717/peerj.13723/supp-14 Supplemental Information 14 Basic information of *COBRA* genes in five Rosaceae species The *COBRA* genes of *Fragaria vesca*, *Prunus mume*, *Rubus occidentalis*, *Malus domestica* and* Prunus avium* identified in this study are listed.

10.7717/peerj.13723/supp-15 Supplemental Information 15 Ka/Ks analysis of the duplicated *COBRA* paralogues

10.7717/peerj.13723/supp-16 Table S4 Detailed information of the 20 motifs in the 87 COBRA proteins

10.7717/peerj.13723/supp-17 Table S5 Gene sequence list

10.7717/peerj.13723/supp-18 Data S1 Raw data of qRT-PCR

## Additional Information and Declarations

### Data Availability

The following information was supplied regarding data availability: The raw measurements are available in the Supplemental Files.
