# Supplementary figures and images for: Comparative genomic analysis of the COBRA genes in six Rosaceae species and expression analysis in Chinese white pear (Pyrus bretschneideri)
Source: PeerJ. 2022 Jul 19;10:e13723. doi: 10.7717/peerj.13723 (PMC9306554; doi:10.7717/peerj.13723)

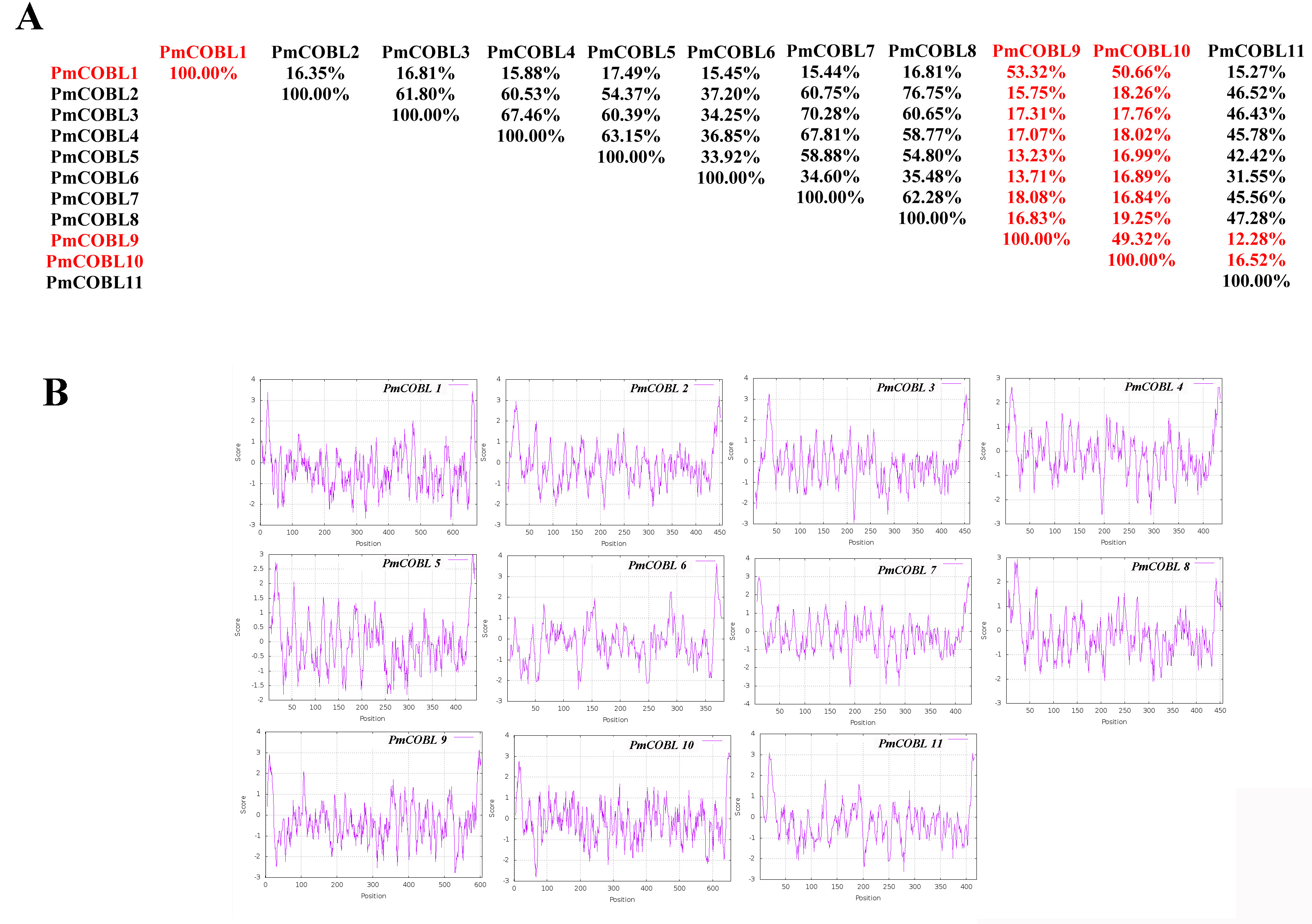

Supplement: Figure S1 [file peerj-10-13723-s001.jpg]

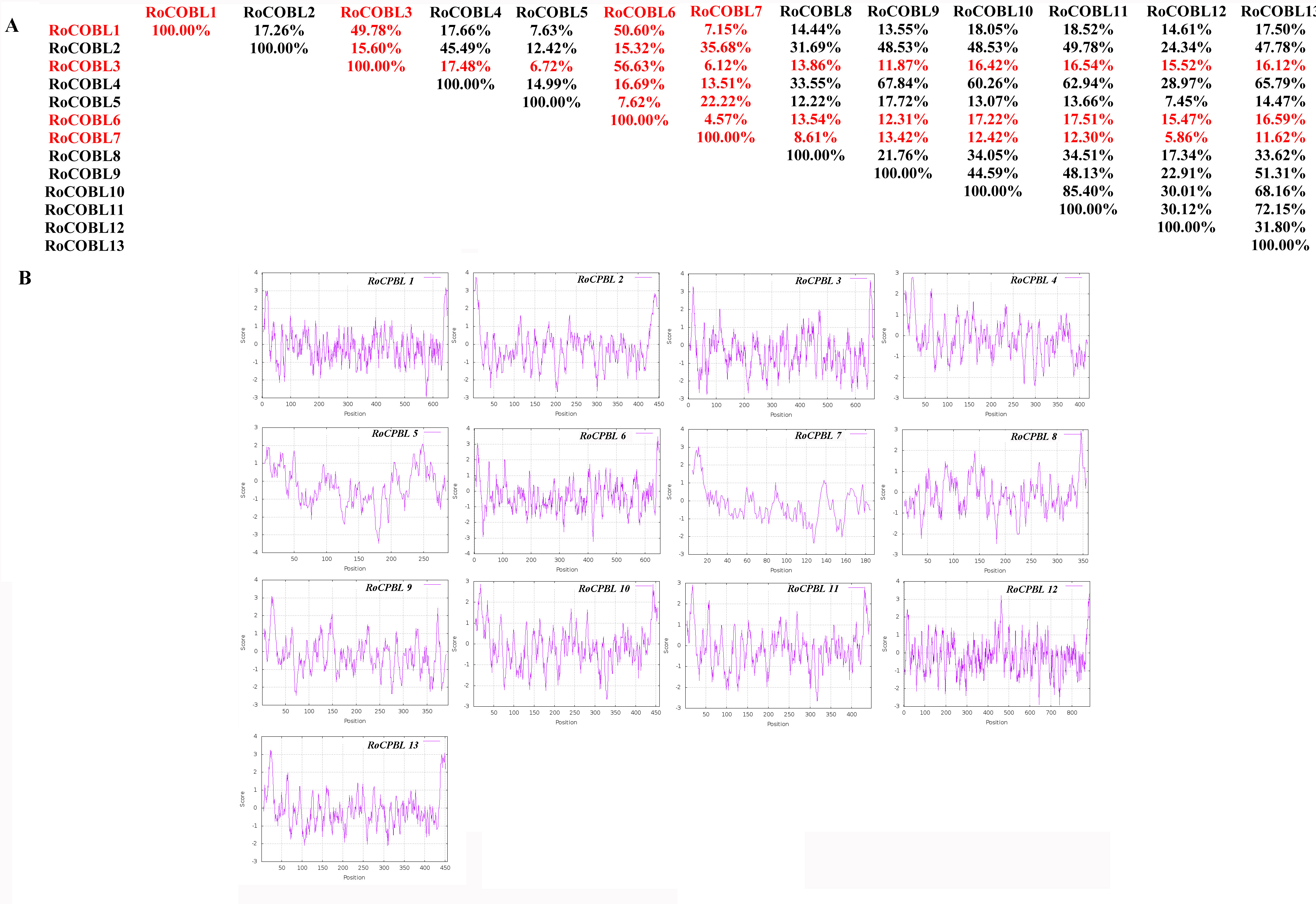

Supplement: Figure S2 [file peerj-10-13723-s002.jpg]

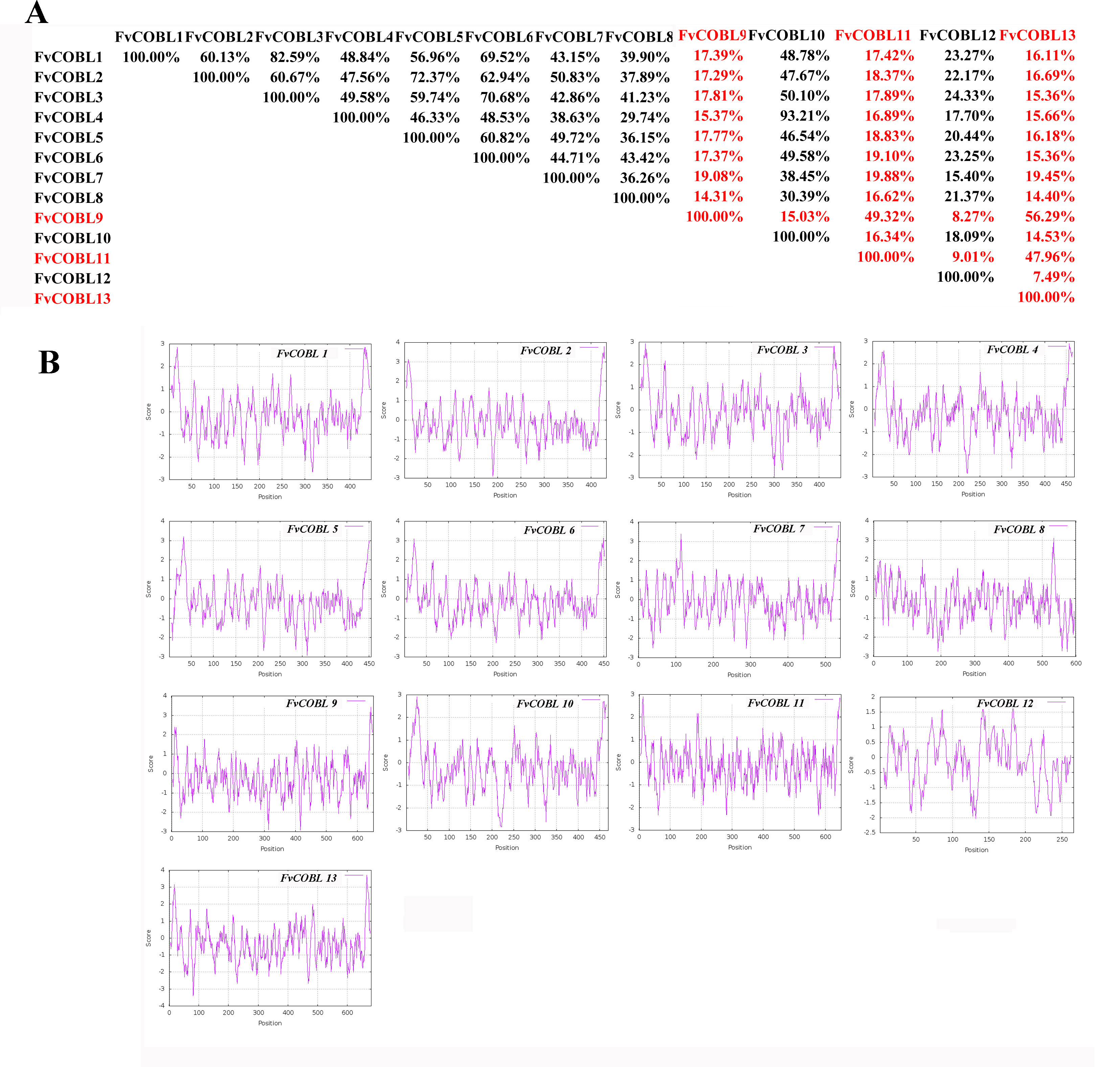

Supplement: Figure S3 [file peerj-10-13723-s003.jpg]

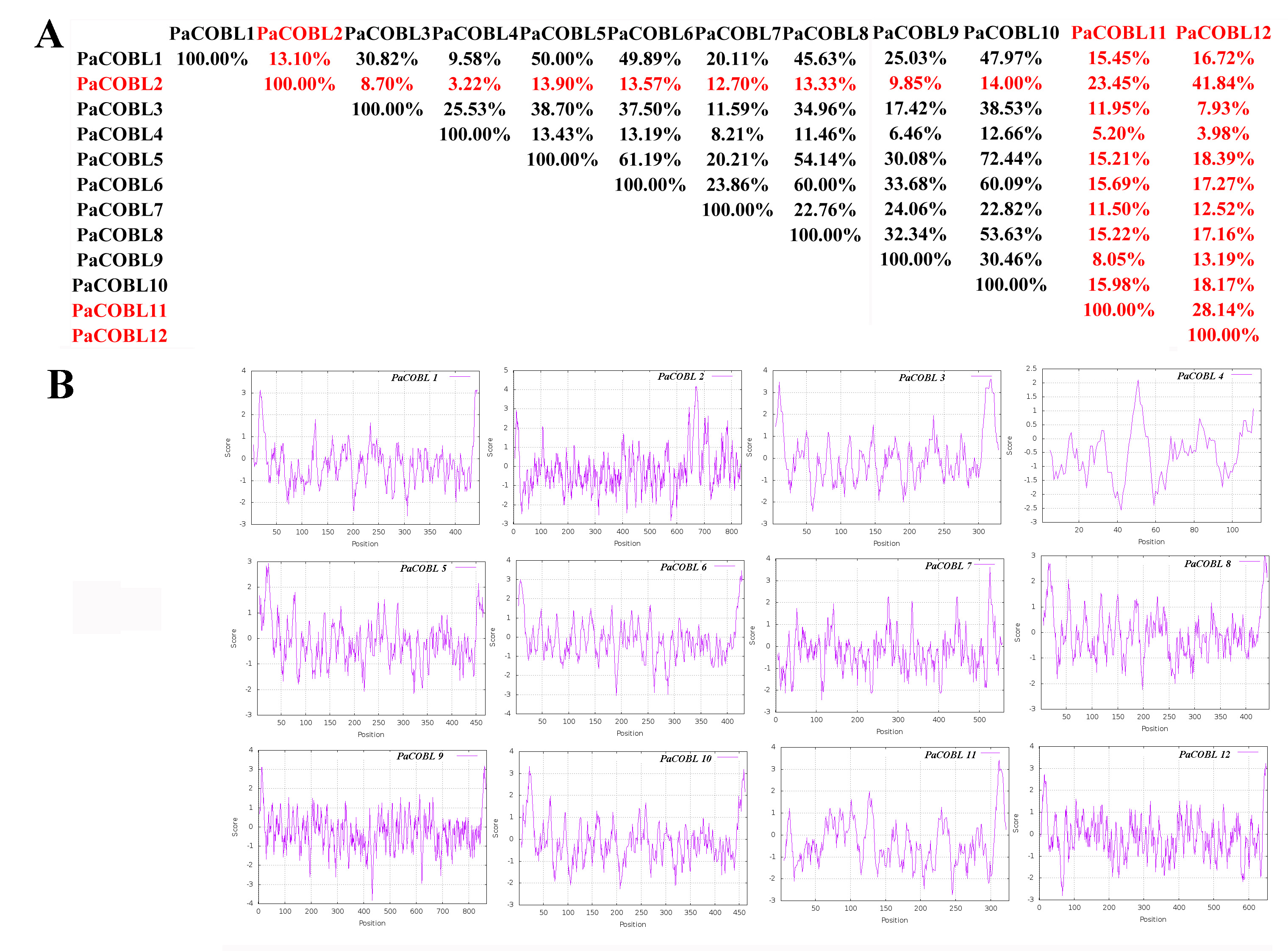

Supplement: Figure S4 [file peerj-10-13723-s004.jpg]

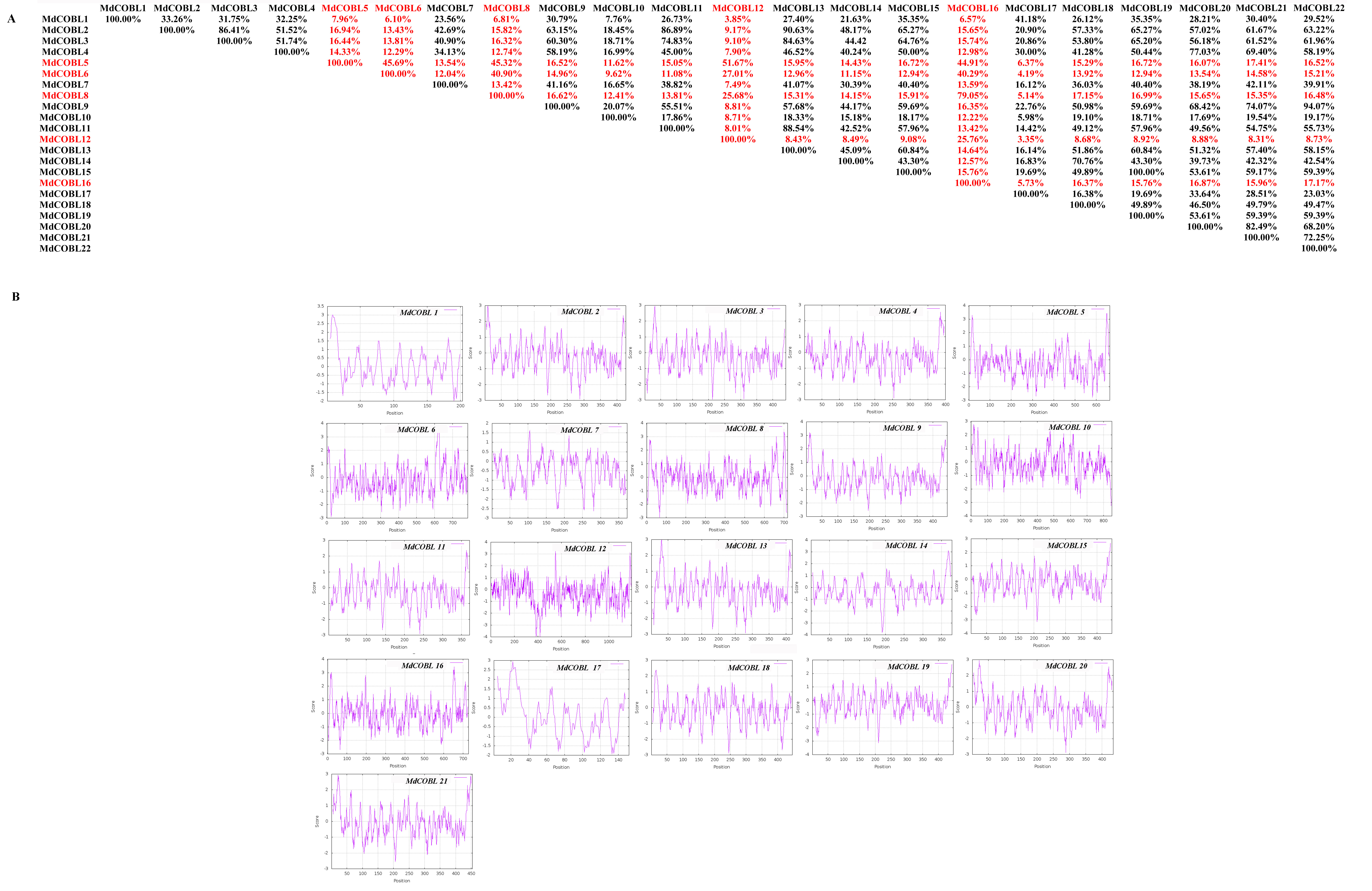

Supplement: Figure S5 [file peerj-10-13723-s005.jpg]

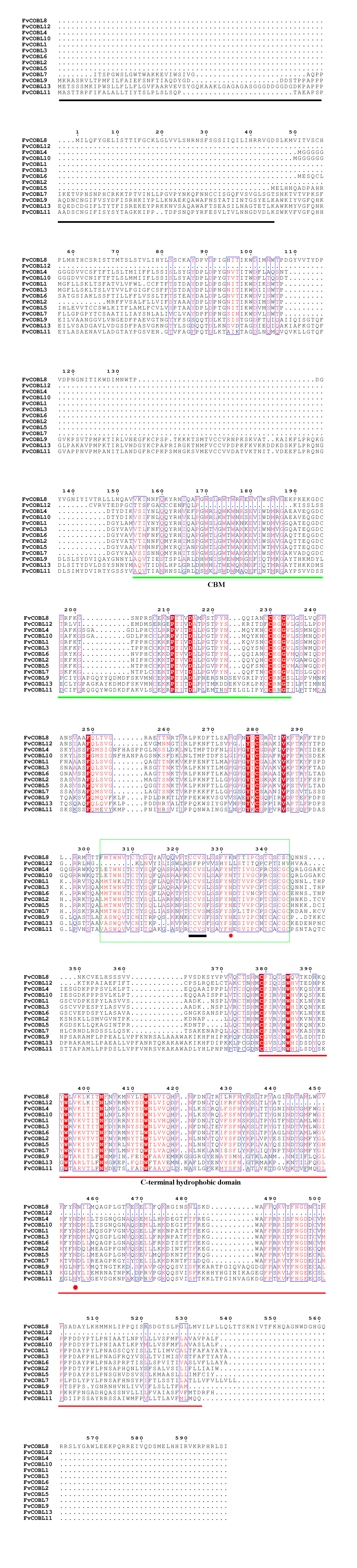

Supplement: Figure S6 [file peerj-10-13723-s006.jpg]

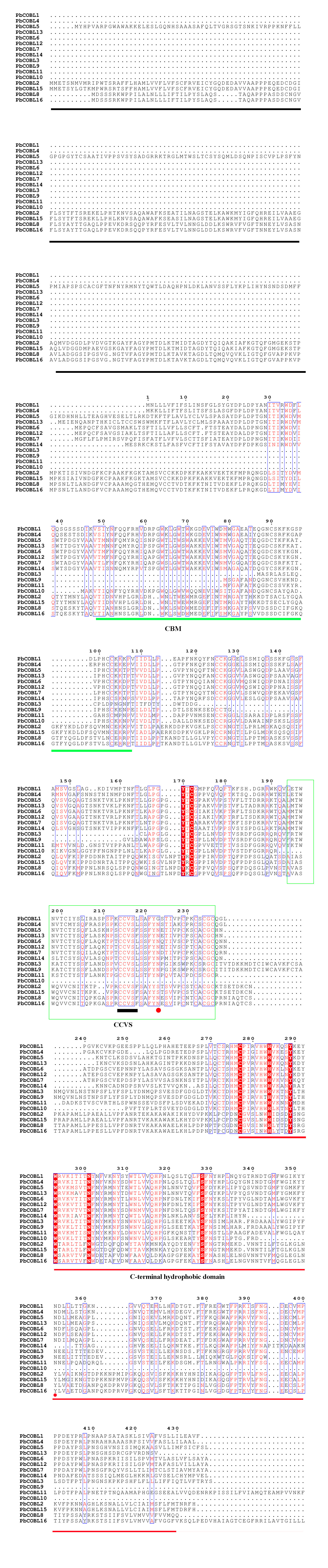

Supplement: Figure S7 [file peerj-10-13723-s007.jpg]

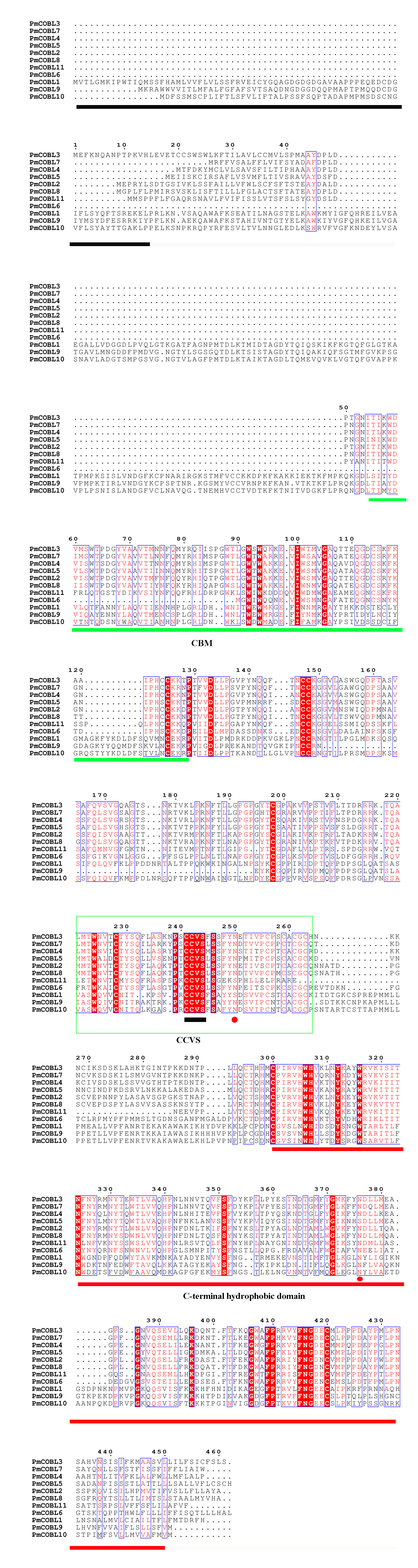

Supplement: Figure S8 [file peerj-10-13723-s008.jpg]

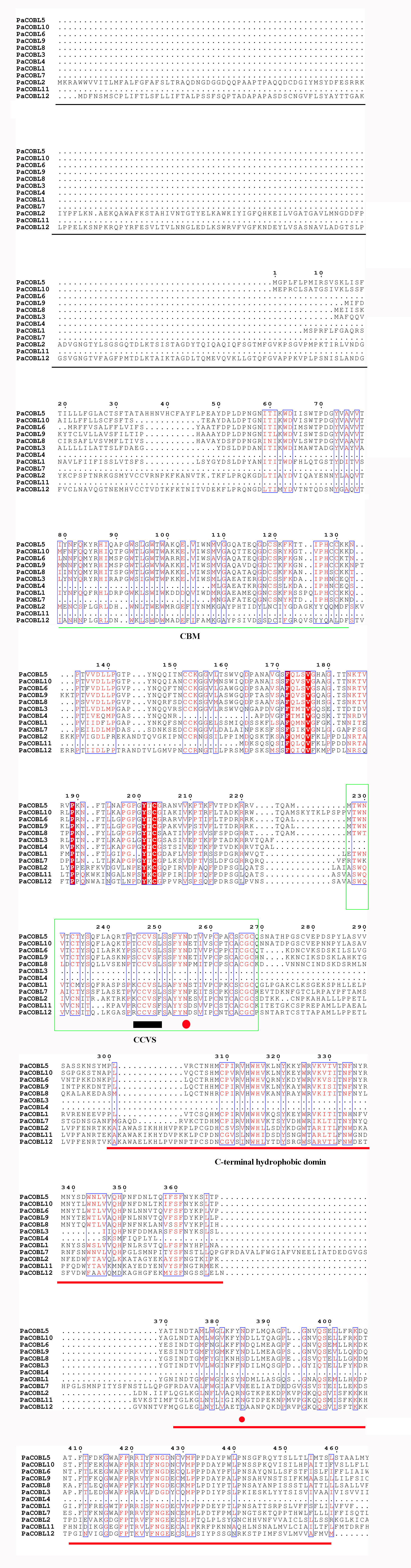

Supplement: Figure S9 [file peerj-10-13723-s009.jpg]

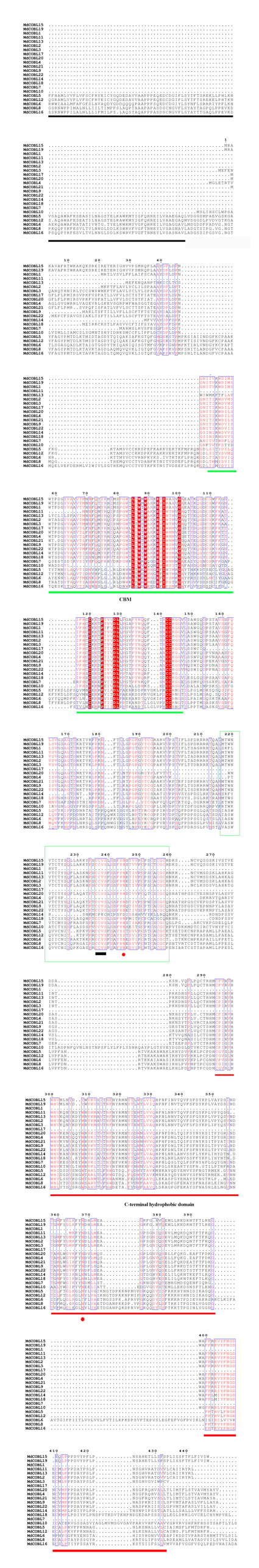

Supplement: Figure S10 [file peerj-10-13723-s010.jpg]

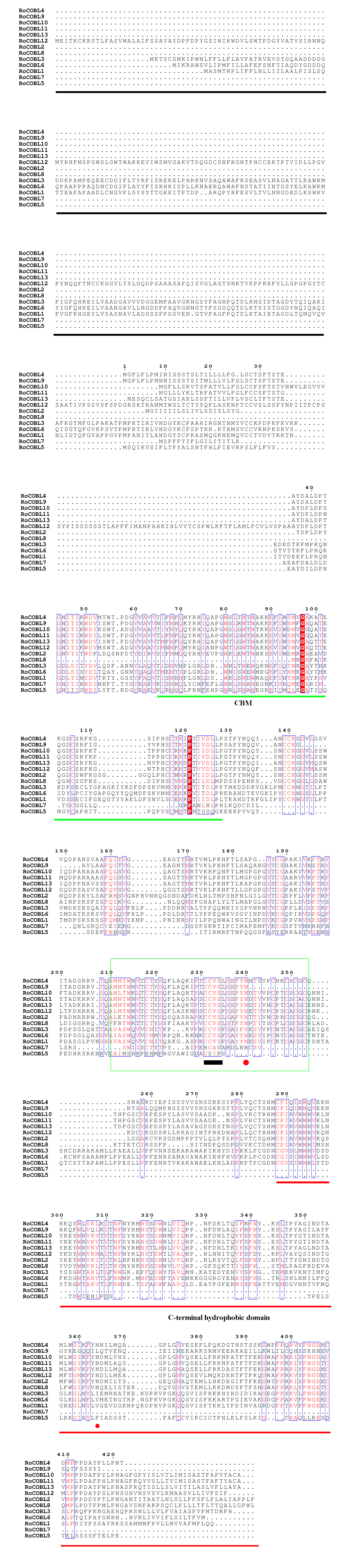

Supplement: Figure S11 [file peerj-10-13723-s011.jpg]

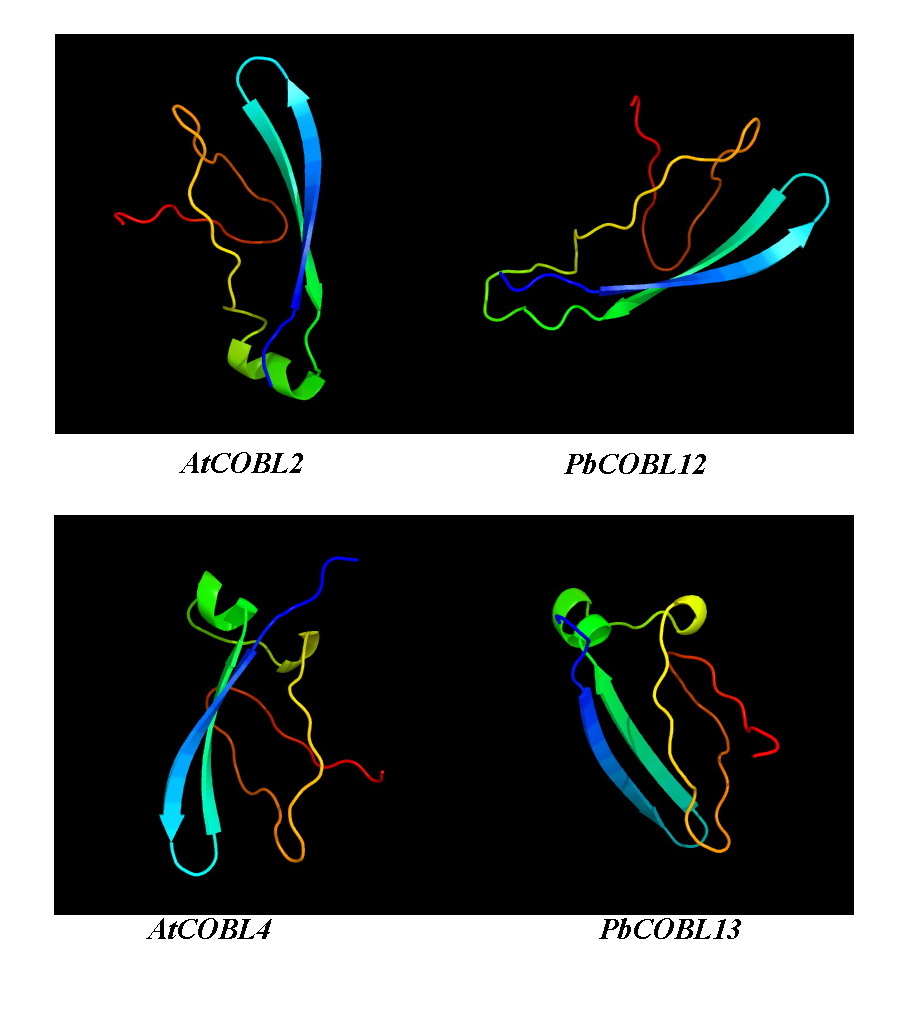

Supplement: Figure S12 [file peerj-10-13723-s012.jpg]
