# Supplementary material for: Comparative genomic analysis of the COBRA genes in six Rosaceae species and expression analysis in Chinese white pear (Pyrus bretschneideri)
Source: PeerJ. 2022 Jul 19;10:e13723. doi: 10.7717/peerj.13723 (PMC9306554; doi:10.7717/peerj.13723)
Supplement: Supplemental Information 13 [file peerj-10-13723-s013.docx]

**Supplementary Table S1. Primers used in qRT-PCR.**

| **Gene name** | **Primer sequences 5’** | **Primer sequences 3’** |
| --- | --- | --- |
| ***PbCOBL1*** | **TGAGACTGAAGAACCATC** | **CTTGTAACTCTGCTTGAC** |
| ***PbCOBL2*** | **CGCAATCTTAGCCTGAATCTGGGT** | **TCATGAGAATACCCTGGACGTCCC** |
| ***PbCOBL3*** | **TGCTTTTGTTTCCTCGCTCAGACC** | **AGCCGCCTAGCGTCGTTAGAACAA** |
| ***PbCOBL4*** | **TGGAATGTGACTTGTATGTA** | **ATGGTGCTATTGTAGAATGA** |
| ***PbCOBL5*** | **GTAAAACGATGGCAGAGGGACAC** | **TCGCGAGGCCTGGGTGGGCATGGG** |
| ***PbCOBL6*** | **GCCTATGATGCTCTTGAT** | **CGCTTGTATATGCCGATA** |
| ***PbCOBL7*** | **AGCAGCAACATAGCCATCAGCAGT** | **ACAATTCATCAGTTTCGCCACCTT** |
| ***PbCOBL8*** | **TTACAATCGCCAACCATA** | **TACAGTCAGAAGAATCAACA** |
| ***PbCOBL9*** | **TGCAGGAGTGTGGTATATCATTCT** | **CCAGTCTGTTGTGTTTCACTCTCT** |
| ***PbCOBL10*** | **TCATTCTGTTGCCAAACCCATCCA** | **GCCTTGTTGGACAACAAATCGGAG** |
| ***PbCOBL11*** | **TGTTCACTGGTGGAGCGTCTGGCA** | **GCAATGCCACTTCCAGACACTTTC** |
| ***PbCOBL12*** | **ATATGTGTCCAATCCGAGTA** | **GCGTGTAGTTCATTCTGTAA** |
| ***PbCOBL13*** | **ACACTGGTATGTTCTATGG** | **GGTATTCTGGTCCTTCTG** |
| ***PbCOBL14*** | **GCTACCTCTTCATCAATACAA** | **TCACAACTCTACAGGCATA** |
| ***PbCOBL15*** | **CATTTTCGTGCCCAGATAACTTGT** | **GCTCTTCTCGTGCCATTCGCTAAC** |
| ***PbCOBL16*** | **CCTTGGTCGTCTTGATAA** | **CAACAACAGATGGATAAGC** |
| ***Tubulin*** | **AGAACAAGAACTCGTCCTAC** | **GAACTGCTCGCTCACTCTCC** |
