# Supplementary material for: Comparative genomic analysis of the COBRA genes in six Rosaceae species and expression analysis in Chinese white pear (Pyrus bretschneideri)
Source: PeerJ. 2022 Jul 19;10:e13723. doi: 10.7717/peerj.13723 (PMC9306554; doi:10.7717/peerj.13723)
Supplement: Supplemental Information 14 — The COBRA genes of Fragaria vesca, Prunus mume, Rubus occidentalis, Malus domestica and Prunus avium identified in this study are listed. [file peerj-10-13723-s014.docx]

**Supplemental Table S2. The *COBRA* genes of apple, strawberry, plum, raspberry and cherry identified in this study are listed.**

| **Gene name** | **Gene ID** | **AA** | **KD** | **pI** | **GRAVY** | **Signal peptide** | **Subdivided subgroup** |
| --- | --- | --- | --- | --- | --- | --- | --- |
| ***FvCOBL1*** | **FvH4_6g46530.1** | **447** | **50.42** | **8.90** | **-0.175** | **Yes** | **COBRA** |
| ***FvCOBL2*** | **FvH4_6g46470.1** | **433** | **48.91** | **8.95** | **-0.134** | **Yes** | **COBRA** |
| ***FvCOBL3*** | **FvH4_6g46460.1** | **448** | **50.19** | **8.95** | **-0.111** | **Yes** | **COBRA** |
| ***FvCOBL4*** | **FvH4_5g28720.1** | **469** | **52.49** | **7.79** | **-0.206** | **Yes** | **COBRA** |
| ***FvCOBL5*** | **FvH4_5g13560.1** | **455** | **50.96** | **8.86** | **-0.196** | **Yes** | **COBRA** |
| ***FvCOBL6*** | **FvH4_5g13550.1** | **456** | **50.70** | **8.83** | **-0.105** | **Yes** | **COBRA** |
| ***FvCOBL7*** | **FvH4_5g13540.1** | **539** | **59.57** | **9.06** | **-0.029** | **No** | **COBRA** |
| ***FvCOBL8*** | **FvH4_3g32820.1** | **599** | **68.02** | **9.22** | **-0.209** | **No** | **COBRA** |
| ***FvCOBL9*** | **FvH4_3g02530.1** | **652** | **72.41** | **9.05** | **-0.270** | **Yes** | **COBL7** |
| ***FvCOBL10*** | **FvH4_1g08930.1** | **470** | **52.55** | **8.19** | **-0.212** | **Yes** | **COBRA** |
| ***FvCOBL11*** | **FvH4_4g19240.1** | **654** | **71.32** | **5.55** | **-0.107** | **Yes** | **COBL7** |
| ***FvCOBL12*** | **FvH4_3g32810.1** | **266** | **29.86** | **8.80** | **-0.088** | **No** | **COBRA** |
| ***FvCOBL13*** | **FvH4_3g21960.1** | **681** | **75.62** | **8.68** | **-0.400** | **Yes** | **COBL7** |
| ***PmCOBL1*** | **pm__Pm029494** | **675** | **75.48** | **8.96** | **-0.340** | **Yes** | **COBL7** |
| ***PmCOBL2*** | **pm__Pm025033** | **456** | **51.22** | **8.88** | **-0.114** | **Yes** | **COBRA** |
| ***PmCOBL3*** | **pm__Pm025032** | **461** | **51.76** | **8.88** | **-0.148** | **Yes** | **COBRA** |
| ***PmCOBL4*** | **pm__Pm025031** | **438** | **49.15** | **8.93** | **-0.136** | **Yes** | **COBRA** |
| ***PmCOBL5*** | **pm__Pm025030** | **445** | **49.24** | **8.60** | **-0.044** | **Yes** | **COBRA** |
| ***PmCOBL6*** | **pm__Pm021030** | **383** | **42.59** | **6.14** | **-0.155** | **No** | **COBRA** |
| ***PmCOBL7*** | **pm__Pm015385** | **432** | **49.16** | **9.06** | **-0.164** | **Yes** | **COBRA** |
| ***PmCOBL8*** | **pm__Pm015384** | **456** | **51.27** | **8.99** | **-0.164** | **Yes** | **COBRA** |
| ***PmCOBL9*** | **pm__Pm009756** | **606** | **68.02** | **9.03** | **-0.330** | **Yes** | **COBL7** |
| ***PmCOBL10*** | **pm__Pm008284** | **655** | **71.82** | **5.95** | **-0.120** | **Yes** | **COBL7** |
| ***PmCOBL11*** | **pm__Pm003928** | **420** | **47.99** | **8.19** | **-0.230** | **Yes** | **COBRA** |
| ***MdCOBL1*** | **MDP0000922681** | **203** | **22.25** | **9.59** | **-0.013** | **Yes** | **COBRA** |
| ***MdCOBL2*** | **MDP0000895592** | **427** | **47.77** | **9.06** | **-0.210** | **Yes** | **COBRA** |
| ***MdCOBL3*** | **MDP0000883782** | **441** | **49.72** | **8.81** | **-0.259** | **Yes** | **COBRA** |
| ***MdCOBL4*** | **MDP0000453207** | **400** | **45.16** | **9.21** | **-0.295** | **No** | **COBRA** |
| ***MdCOBL5*** | **MDP0000319310** | **664** | **74.44** | **9,00** | **-0.333** | **Yes** | **COBL7** |
| ***MdCOBL6*** | **MDP0000308519** | **785** | **87.52** | **9.29** | **-0.202** | **Yes** | **COBL7** |
| ***MdCOBL7*** | **MDP0000304256** | **373** | **42.48** | **7.44** | **-0.509** | **No** | **COBRA** |
| ***MdCOBL8*** | **MDP0000290819** | **718** | **78.52** | **7.34** | **-0.072** | **Yes** | **COBL7** |
| ***MdCOBL9*** | **MDP0000288732** | **445** | **49.90** | **9.00** | **-0.117** | **Yes** | **COBRA** |
| ***MdCOBL10*** | **MDP0000279069** | **850** | **94.86** | **7.66** | **-0.175** | **Yes** | **COBRA** |
| ***MdCOBL11*** | **MDP0000274376** | **371** | **41.57** | **9.26** | **-0.325** | **No** | **COBRA** |
| ***MdCOBL12*** | **MDP0000236092** | **1193** | **131.89** | **7.61** | **-0.343** | **No** | **COBL7** |
| ***MdCOBL13*** | **MDP0000235408** | **419** | **47.1** | **9.11** | **-0.238** | **No** | **COBRA** |
| ***MdCOBL14*** | **MDP0000235407** | **378** | **42.8** | **9.36** | **-0.301** | **No** | **COBRA** |
| ***MdCOBL15*** | **MDP0000225330** | **447** | **50.7** | **9.21** | **-0.240** | **No** | **COBRA** |
| ***MdCOBL16*** | **MDP0000224741** | **729** | **79.49** | **5.04** | **-0.029** | **Yes** | **COBL7** |
| ***MdCOBL17*** | **MDP0000209592** | **152** | **17.1** | **6.68** | **0.104** | **Yes** | **COBRA** |
| ***MdCOBL18*** | **MDP0000170638** | **446** | **49.48** | **8.39** | **-0.202** | **Yes** | **COBRA** |
| ***MdCOBL19*** | **MDP0000170361** | **447** | **50.69** | **9.21** | **-0.240** | **No** | **COBRA** |
| ***MdCOBL20*** | **MDP0000170360** | **435** | **48.98** | **8.57** | **-0.074** | **Yes** | **COBRA** |
| ***MdCOBL21*** | **MDP0000131777** | **453** | **51.06** | **8.97** | **-0.150** | **Yes** | **COBRA** |
| ***MdCOBL22*** | **MDP0000094767** | **454** | **50.68** | **8.92** | **-0.081** | **Yes** | **COBRA** |
| ***RoCOBL1*** | **Bras_G23788** | **653** | **71.54** | **5.31** | **-0.147** | **Yes** | **COBL7** |
| ***RoCOBL2*** | **Bras_G23210** | **451** | **50.98** | **7.1** | **-0.186** | **Yes** | **COBRA** |
| ***RoCOBL3*** | **Bras_G19790** | **668** | **75.03** | **8.75** | **-0.405** | **Yes** | **COBL7** |
| ***RoCOBL4*** | **Bras_G16393** | **422** | **47.05** | **8.65** | **-0.134** | **Yes** | **COBRA** |
| ***RoCOBL5*** | **Bras_G11090** | **288** | **32.98** | **6.15** | **-0.283** | **No** | **COBRA** |
| ***RoCOBL6*** | **Bras_G10559** | **654** | **73.10** | **9.02** | **-0.290** | **Yes** | **COBL7** |
| ***RoCOBL7*** | **Bras_G09540** | **189** | **21.75** | **9.03** | **-0.185** | **Yes** | **COBL7** |
| ***RoCOBL8*** | **Bras_G08009** | **360** | **40.20** | **6.63** | **-0.123** | **No** | **COBRA** |
| ***RoCOBL9*** | **Bras_G07502** | **395** | **44.92** | **8.81** | **-0.182** | **Yes** | **COBRA** |
| ***RoCOBL10*** | **Bras_G04907** | **459** | **51.26** | **8.78** | **-0.107** | **Yes** | **COBRA** |
| ***RoCOBL11*** | **Bras_G04899** | **447** | **49.89** | **8.73** | **-0.119** | **Yes** | **COBRA** |
| ***RoCOBL12*** | **Bras_G03362** | **886** | **98.73** | **8.98** | **-0.166** | **Yes** | **COBRA** |
| ***RoCOBL13*** | **Bras_G03361** | **456** | **50.70** | **8.74** | **-0.092** | **Yes** | **COBRA** |
| ***PaCOBL1*** | **Pav_sc0001196** | **447** | **50.82** | **8.49** | **-0.229** | **Yes** | **COBRA** |
| ***PaCOBL2*** | **Pav_sc0000910** | **837** | **93.00** | **9.13** | **-0.173** | **Yes** | **COBL7** |
| ***PaCOBL3*** | **Pav_sc0000661** | **333** | **37.36** | **5.13** | **0.014** | **Yes** | **COBRA** |
| ***PaCOBL4*** | **Pav_sc0000661** | **115** | **12.59** | **9.51** | **-0.455** | **No** | **COBRA** |
| ***PaCOBL5*** | **Pav_sc0000661** | **469** | **52.74** | **9.02** | **-0.118** | **Yes** | **COBRA** |
| ***PaCOBL6*** | **Pav_sc0000661** | **432** | **49.15** | **9.07** | **-0.145** | **Yes** | **COBRA** |
| ***PaCOBL7*** | **Pav_sc0000568** | **560** | **62.57** | **5.88** | **-0.249** | **No** | **COBRA** |
| ***PaCOBL8*** | **Pav_sc0000358** | **445** | **49.27** | **8.52** | **-0.047** | **Yes** | **COBRA** |
| ***PaCOBL9*** | **Pav_sc0000358** | **867** | **97.63** | **8.95** | **-0.258** | **Yes** | **COBRA** |
| ***PaCOBL10*** | **Pav_sc0000358** | **446** | **52.04** | **9.01** | **-0.102** | **Yes** | **COBRA** |
| ***PaCOBL11*** | **Pav_sc0000218** | **326** | **36.54** | **8.93** | **-0.376** | **No** | **COBL7** |
| ***PaCOBL12*** | **Pav_sc0000174** | **654** | **71.52** | **6.05** | **-0.129** | **Yes** | **COBL7** |
