# Supplementary material for: Comparative genomic analysis of the COBRA genes in six Rosaceae species and expression analysis in Chinese white pear (Pyrus bretschneideri)
Source: PeerJ. 2022 Jul 19;10:e13723. doi: 10.7717/peerj.13723 (PMC9306554; doi:10.7717/peerj.13723)
Supplement: Supplemental Information 15 [file peerj-10-13723-s015.docx]

| **Duplicated gene pairs** | **Ka** | **Ks** | **Ka/Ks** | **Purifying selection** | **Duplicated type** |
| --- | --- | --- | --- | --- | --- |
| ***PbCOBL1*-*PbCOBL4*** | **0.1172** | **0.3467** | **0.338** | **Yes** | **Segmental** |
| ***PbCOBL2*-*PbCOBL15*** | **0.0267** | **0.1676** | **0.159** | **Yes** | **Segmental** |
| ***PbCOBL6*-*PbCOBL12*** | **0.0196** | **0.2178** | **0.090** | **Yes** | **Segmental** |
| ***PbCOBL8*-*PbCOBL16*** | **0.0047** | **0.0216** | **0.218** | **Yes** | **Segmental** |
| ***FvCOBL1*-*FvCOBL3*** | **0.2688** | **0.5585** | **0.481** | **Yes** | **Tandem** |
| ***FvCOBL4*-*FvCOBL10*** | **0.0977** | **0.2017** | **0.484** | **Yes** | **Segmental** |
| ***MdCOBL2*-*MdCOBL13*** | **0.0459** | **0.0656** | **0.699** | **Yes** | **Segmental** |
| ***MdCOBL3*-*MdCOBL13*** | **0.0846** | **0.0665** | **1.272** | **No** | **Segmental** |
| ***MdCOBL4*-*MdCOBL20*** | **0.0833** | **0.1056** | **0.788** | **Yes** | **Segmental** |
| ***MdCOBL8*-*MdCOBL16*** | **0.1301** | **0.1340** | **0.971** | **Yes** | **Segmental** |
| ***MdCOBL9*-*MdCOBL22*** | **0.0580** | **0.0914** | **0.635** | **Yes** | **Segmental** |
| ***MdCOBL20*-*MdCOBL21*** | **0.0819** | **0.0828** | **0.989** | **Yes** | **Segmental** |
| ***PmCOBL2*-*PmCOBL8*** | **0.1475** | **1.0977** | **0.134** | **Yes** | **Segmental** |
| ***RoCOBL10*-*RoCOBL11*** | **0.1872** | **0.1640** | **1.141** | **No** | **Tandem** |

**Supplementary Table S3. Ka/Ks analysis of the duplicated *COBRA* paralogues.**
