# Supplementary material for: Comparative genomic analysis of the COBRA genes in six Rosaceae species and expression analysis in Chinese white pear (Pyrus bretschneideri)
Source: PeerJ. 2022 Jul 19;10:e13723. doi: 10.7717/peerj.13723 (PMC9306554; doi:10.7717/peerj.13723)
Supplement: Table S4 [file peerj-10-13723-s016.docx]

**Supplementary Table S4 . Complete information on *Cis*-acting elements of the 16 *PbCOBL* genes.**

| **Site Name** | **Matrix score** | **Sequence** | **Function** |
| --- | --- | --- | --- |
| **TGA-box** | **10** | **TGACGTAA** | **part of an auxin-responsive element** |
| **CGTCA-motif** | **16** | **CGTCA** | **cis-acting regulatory element involved in the MeJA-responsiveness** |
| **TGACG-motif** | **16** | **TGACG** | **cis-acting regulatory element involved in the MeJA-responsiveness** |
| **P-box** | **1** | **CCTTTTG** | **gibberellin-responsive element** |
| **TCA-element** | **12** | **CCATCTTTTT** | **cis-acting element involved in salicylic acid responsiveness** |
| **G-box** | **46** | **TACGTG** | **cis-acting regulatory element involved in light responsiveness** |
| **MBS** | **6** | **CAACTG** | **MYB binding site involved in drought-inducibility** |
| **AuxRE** | **2** | **TGTCTCAATAAG** | **part of an auxin-responsive element** |
| **TATC-box** | **1** | **TATCCCA** | **cis-acting element involved in gibberellin-responsiveness** |
| **GARE-motif** | **1** | **TCTGTTG** | **gibberellin-responsive element** |
| **ABRE** | **43** | **CACGTG** | **cis-acting element involved in the abscisic acid responsiveness** |
| **ARE** | **29** | **AAACCA** | **cis-acting regulatory element essential for the anaerobic induction** |
| **LTR** | **32** | **CCGAAA** | **cis-acting element involved in low-temperature responsiveness** |
| **TC-rich repeats** | **3** | **GTTTTCTTAC** | **cis-acting element involved in defense and stress responsiveness** |
| **GT1-motif** | **38** | **GGTTAA** | **light responsive element** |
| **GATA-motif** | **13** | **AAGATAAGATT** | **part of a light responsive element** |
| **Sp1** | **10** | **GGGCGG** | **light responsive element** |
| **Box 4** | **26** | **ATTAAT** | **part of a conserved DNA module involved in light responsiveness** |
| **TCT-motif** | **5** | **TCTTAC** | **part of a light responsive element** |
| **AE-box** | **1** | **AGAAACTT** | **part of a module for light response** |
| **LAMP-element** | **1** | **CTTTATCA** | **part of a light responsive element** |
| **chs-CMA1a** | **4** | **TTACTTAA** | **part of a light responsive element** |
| **GA-motif** | **1** | **ATAGATAA** | **part of a light responsive element** |
| **I-box** | **4** | **GATAAGGGT** | **part of a light responsive element** |
| **3-AF1 binding site** | **2** | **TAAGAGAGGAA** | **light responsive element** |
| **ACE** | **2** | **CTAACGTATT** | **cis-acting element involved in light responsiveness** |
| **TCCC-motif** | **1** | **TCTCCCT** | **part of a light responsive element** |
| **O2-site** | **10** | **GTTGACGTGA** | **cis-acting regulatory element involved in zein metabolism regulation** |
| **CAT-box** | **5** | **GCCACT** | **cis-acting regulatory element related to meristem expression** |
| **AACA_motif** | **1** | **TAACAAACTCCA** | **involved in endosperm-specific negative expression** |
| **WUN-motif** | **1** | **AAATTACT** | **wound-responsive element** |
| **GCN4_motif** | **1** | **TGAGTCA** | **cis-regulatory element involved in endosperm expression** |
