# Supplementary material for: Comparative genomic analysis of the COBRA genes in six Rosaceae species and expression analysis in Chinese white pear (Pyrus bretschneideri)
Source: PeerJ. 2022 Jul 19;10:e13723. doi: 10.7717/peerj.13723 (PMC9306554; doi:10.7717/peerj.13723)
Supplement: Table S5 [file peerj-10-13723-s017.docx]

>PbCOBL1

MNLLLVFIFSLINSFGLSYGYDPLDPYANITVRWDFLQQSGSSSDIKVSIYNFQQFRHVDRPGWKLGWTWKGDEVIWDMWGAEATEQGNCSKFKGSPDLPHCCKKKPVIIDLLPEAPFNKQYFNCCKGGELSSMIQDSSKFGSSFAMSVGSLAGKDIVMPTNFTLGLPGYTCGMPFQVQPTKFSHDGRRWKQVLETWNVTCIYSLIRASPSPKCCVSLSAFYGSTIVPCPKCSCGCQGLPGVKCVKPGEESPPLLQLPHAHETEEPSPLVTCSRHMCPIRVHWHVKQSYKEYWRVKITITNYNFVKNYSYWTLVVQHPNLQSLTQLFSFNYHPLNQYGTRNDTGMFWGIKYYNDLLLTTGKNGVVQTEMLLHKDTGTFTFREGWTFPRKISFNGDECVMPPPDEYPRLPNAAPSATASKLSIVAFVSLLILEAVF

>PbCOBL2

MMETSNMVMRIPWTSRAFFLHAMLVVFLVFSCFRVEICYGQDEDAVVAAPPPEQEDCDGIFLSYTFTSREKELPHTKNVSAQAWAFKSEATILNAGSTELKAWKMYIGFQHREILVAAEGAQMVDGGDLPVDVGTKGAYFAGYPMTDLKTMIDTAGDYTQIQAKIAFKGTQFGMGEKSTPMPKTISIVNDGFKCPAAKFKGKTAMSVCCKKDPKFKAKKVEKTKFMPRQNGDLSITYDVMQTYTMNYLAQVTIDNVHPLGRLDHWNLTWEWMRGEFINTMRGAYTHKKDTSACLYGQAGKFYKDLDFSQVMNCEKKPVITDLPAERKDDPKVGKLPSCCRNGTILPRLMDKAQSQSIFQLQVFKIPPDDNRTAITPPQQWKISGVLNPTYRCGPPIRVDPTQFPDPSGLQATSDAIASWQVVCNITKTPVPRCCVSFSAYYSTSVVPCSTCACGCKTSETDKCNPKAPAMLLPAEALLVPFANRTEKAKAWAKIKHYDVPKKLPCPDNCGVSLNWHIDSDYSNGWTARLTLFNWGTDQFQDWYTAVKMNKAYQDYENVYSFNGTRMEKDVNNTILFTGLKGLNYLVAIKNGTDPKKNPMIPGKQQSVISFKKKHYHNIDIKAGQGFPTRVLFNGEECALPKVFPKNNAGHLKSNALLVLCIAIMSFLFMTNRFH

>PbCOBL3

MASRLASLEQCPLDPNGNFTITFDTYDWTDDGCLAPGLGYTCGPLLNVDPTVSRDFGGRRQRQVFRTWKATCTYSSFLANDSPVCCVSLSSFYNPGIKSWPKCSRGCITVTDKKMDTCIWCAVKFCSANMQVNLNSTNPSFLYFSPLYDNMQPSVESDFGDGLDTVKCTDHMCPIRVHWHVKTNYLTHWRVKLTITNYNYMKNYSNWNVLGQHPGLSEETKAYSFNSIMLHARFRDAAALYWGIPYFNEELITTTEDEVGSVSTEILLEKNSDSFTFKNGRGFPRRVYFNGENCEMPLSDTFPTLPNGNSKPKPSHFLFLLLIFFIQTLVFTRYS

>PbCOBL4

MKKLLIFTFSLITSFSLASGFDPLDPYANITVTWDFLQQSESTSDIKVSIYNFQQFRHVDRPGWKLGWIWKGDEVIWNMWGAEAIYQGNCSRFKGAPERPHCCEKEPVIIDLLPGTPFNKQFSNCCKGGELSSMIQDSSKFLSAFRMNVGAFSNNSTNINMPDNFTLGLPGYTCGPPVQVQPTKTSQDGRRWTETLETWNVTCMYSQFRASPSPKCCVSLSSFYNSTIAKCPRCSCGCQGLPGAKCVKPGDELQLPGDRETEDPSPLVTCTRHMCPIGIHWHVKESYKEYWRVKITITNYNFVKNYSDWTLVVQHPNLQSLTQLFSFNYHPLGQYGNINDTGMFWGIKYYNDMLLSTGKNGNVQTEMVLHKDEGVFTFREGWTFPRRISFNGDECVMPSPDEYPRLPNRAHRAAASRPSIVVFASLLILAAL

>PbCOBL5

MYHPVARPGWAWAKKELESLGQNHSAAASAFQLTVGRSGTSNKIVRPPKNFFLLGPGPGYTCSAATIVPPSVSYSADGRRKTRGLMTWSLTCSYSQMLDSQNPISCVPLPSFYNPMIAPSPSCACGFTNFNYRMNYTQWTLDAQHPNLDKLANVSSFLYKPLIHYNSNDSDMFFGIKDHNHLLTEAGHVESELTLRKDTKFTFLAVLYCLVLSPASAYDPLDPTGNITIKWDVMSWTPDGYVAAVTMNNFQMYRQISNPGWTLTWSWAKKEVIWTMVGAQTTEQGDCTKFKATIPHCCKKTPTVVDLLPGVPYNQQFTNCCKGGVLASWGQDPLAAVSGFQVSVGQAGTSNKTVKLPKNFTLLGPGPGYTCGPAKVVPSTVFLTTDRRRKTQALMTWNVTCTYSQFLASKHPSCCVSFSSFYNETIVPCKSCACGCHNKNTCLKSDSVLAHKTGINTPKKDNSPLLQCTHHMCPIRVHWHVKQNYKDYWRVKMSVTNFNYRMNYTEWTLVAQHPNLNNVTQVFSFDYKPLIPYQSVNDTGMFYGMKFYNDLLMEAGPLGNIQSEVLMRKDQNTFTFKQGWAFPRRVYFNGDECMLPPPDAYPSLPNSGHVNSISIMQKAASVLLIMFSICFSL

>PbCOBL6

MEPQCFSAVGSMAKLTSFTILLVFLLSCFTFTSTEAYDALDPNGNITIKWDVISWTPDGYVAVVTMFNFQQYRHIQAPGWTLGWTWAKKEVIWSMVGAQATEQGDCSKYKGNVPHCCKKDPTIVDLLPGTPYNQQIANCCKGGVMQSWIQDPDNAISSFQISVGAAGTTNKTVRLPKNFTLKAPGPGYSCGIAKIVKPTRFLTSDKRRWTQAMMTWNVTCTYSQFLAQKTPTCCVSLSSFYNQTIVSCPTCACGCQNNATDPGSCVEPNNPYLASAVSGSGKSANTPLVQCTSHMCPIRVHWHVKLNYKEYWRVKVTITNFNYRMNYTLWNLVVQHPNFDNLTKIFSFNYKSLTPYVGLNDTAMLWGVKFYNDFLSQAGPLGNVQSELLFRKDTSTFTFEKGWAFPRRIYFNGDNCVMPPPDSYPWLPNASPKRIISILSPVMTVLASLVFLSAYA

>PbCOBL7

MGFLFLPMIRSVPQFISFATFLVFVLSCTTSFIATEAYDPLDPNGNITIKWDIISWTADGYVAAVTLFNFQKYRHIQAPGWSLGWTWAKKEVIWNMVGGQATEQGDCSRFKTTLPHCCKKTPTIVDLLPGTPYNQQYTNCCKGGVLSSWVQDPEKAVGSFQISVGQAGTTNKTVRLPKNFTLNTPGPGYTCARAKVVKPTKFISPDKRRVTQAMMTWNVTCTYSQILAQKTPTCCVSLSSFYNDTIVPCPKCSCGCQRNATHPGSCVEPDSPYLASVVSASNKNSYTPLVRCTKHMCPIRVHWHVKVNYKQYWRVKVTVTNFNYRMNYSDWNMVVQHPNFDNLTQSFSYNYKSITPYATINDTGMLWGIKFYNDILMQAGPLGNVQSELLFRKDEATFTFDKGWAFPRRVYFNGDNCVMPPPDAYPWLPNSGFRQYVSLLTLIMTCLSTIAVMYAYA

>PbCOBL8

MDSSSRKWPPILALNLLLIFTILPYSLAQSTAQAPPPASDSCNGVFLSYAYTTGAQLPPEVKDRSQQPYRFESVLTVLNNGLDDLKSWRVFVGFTNNEYLVSASNAVLADGGSIPGSVGNGTVFAGYPMTDLKTAVKTAGDLTQMQVQVKLIGTQFGVAPPKVPMPSNLTLANDGFVCPAAAMQGTHEMQVCCTVDTKFKTNITVDEKFLPRQKGDLTIMYEVISTQESKYTAQVTIANHNSLGRLDNWKLSWDWMEDEFIFSMKGAYPSVVDSSDCIFGKQGTFYQGLDFSTVLNCERRPTIIDLPPTKANDTLLGLVPYCCRNGTILPPTMDASKSVSSFQIQVFKMPPNLNRSQLSPPQNWGINGTLNPDYKCGPPVQVSPSQFPDQSGLPVNSTAVASWQVVCNITQPKGASPRCCVSFSAFYNESVIPCNTCACGCPRNIAQTCSTTAPAMLLPPESLLVPFDNRTVKAKAWAELKHLPDPNPTPCGDNCGVSINWHLYTDYSRGWSARVTVFNWDETAFVDWFAAVQLDKAGPGFEKAYSFNASHLELNGVNNTVFMQGLEGLNYLVAETDGANPQKDPRVPGKQQSVLSFTKKTTPGINLVGGDGFPTKVYFNGEECSLPTIYPSSAYRKSTSIIFSVLVMVVVFVVMQQ

>PbCOBL9

MSGAFANDQGNCSVHKNDIPHSCKENPEILDLTPDTLSENKSEDCCTGGVLDAWAPSLGYTCGPLLNVDPTVSRDFGGRRQRQVFRTWKATCTYSSFLANDSPVCCVSLSSFYNPGIKSWPKCSRGCITITDKKMDTCIWCAVKFCTANMQVNLNSTNPSFLYFSPLYDNMQPSVESDFGDGLDTVKCTDHMCPIRVHTHVKTNYLTHWRVKLTITNYNYMKNFSNWNVLGQHPGLSEETKAYSFNSIMLHARFRDAAALYWGIPYFNEELITTTEDEVGSVSTEILLEKNSRLLHIQKWTGLPQKSIFQW

>PbCOBL10

MAKVTIQNFYQYRHVDKPGWQLGWVWQQNEVIWSITGAFANDQGNCSAYQADIPHSCKKNPVIVDLTPDALLDNKSEDCCRGGVLDAWAINPSKSLSSFKIKVGNLGGYPFNGNPPLNLTLKAPGLGYTCGPLLNVDPTVSTDFGGRRQRPVFTYPLRTSVEYDGDGLDTVKCTDHMCPIRVHWHLKTNYLTHWRVKLTITNYNYMKNFSNWNVLVQHPGLSKEARTYSFNSTILPTGFRD

>PbCOBL11

MFGAFATKQGDCSSVKYRGAHSCMPNPVIVDLMPDAPPVNMSENCCRGGLISARAIDPLRSFSSFEMTVVNLDGNSTVYPPANLTLWAPGPGYTCGPVVETTPTVSSDFGGRRQVQVYKTWKSTCTYSTFLANKTPGCCVSLSTFYNPQVTACPDCSCGCRDADKSTVSCVRTHLSPWNSSEVDSFPLSDVEKADIVQCTDHMCPVRVHWHVKNNYMDHWRVKMTISNYNYKKNYLDWNVLVQHPGFKQNSTTFSFNSTLLPAAGFADEVALFWGLQAYNNELPQADQRQLGSVSTEILFEKDSGMFTLRNGWALPRRIYFSGEECAMPLPDTFPALPNETPTNQAAMAPHGEGSEEALVVQDENRKPISSILFVIAMQTEAMPVVNKFQLKEDLDPVFPKGVPWVRYHGIYKDLHINLVWPGKDLTLGVDSVGTISASLVTYAAIQALQPDLIINAGTAGGFKVKGACISDVFLVSDVAFHDRRIPIPVFDLYGVGSRQSFSTPNLLNELNLKVGKLSTGDSLDMSPQDEASIVANDATVKDMEGAAVAYVADLLKVPAIFIKAVTDIVDGDKPTAEEFLQNLAAVTAALDQAVAQVVDFISGKCLSEL

>PbCOBL12

MEPQCFSAVGSIAKLTSFTILLAFLLSCFTFTSTEAYDALDPTGNITIKWDVISWTPDGYVAVVTMFNFQQYRHIQAPGWTLGWTWAKKEVIWSMVGAQTTEQGDCSKYKGNVPHCCKKDPTVVDLLPGTPYNQQIANCCKGGVMQSWIQDPPNAISSFQVSVGAAGTTNKTVRLPKNFTLRAPGPGYSCGIAKIVKPTRFLTSDKRRWTQAMMTWNVTCTYSQFLAQKTPTCCVSLSSFYNETIVSCPTCACGCQNNATEPGSCVEPKNPYLASAVAGSGKSANTPLVQCTSHMCPIRVHWHVKLNYKDYWRVKVTITNFNYRMNYTLWNLVVQHPNFDNLTKIFSFNYKSLTPYVGLNDTAMLWGVKFYNDFLSEAGPLGNVQSELLFRKDTSTFTFEKGWAFPRRIYFNGDNCVMPPPDAYPWLPNASPKRIISILGPVMTAFASLVILLAYA

>PbCOBL13

MEIENQANPTHKICLTCCSWSWMKFTFLAVLYCLMLSPAAAYDPLDPTGNITIRWDVMSWTTDGYVAAVTMNNFQMYRQIPSPGWTLGWSWAKKEVIWTMVGAQTTEQGDCSKFKATIPHCCKKTPTVVDLLPGVPYNQQFANCCKGGVVASWGQDPSAAVAGFQVSVGQAGTSNKTVKLPKNFTLLGPGPGYTCGPAKVVPSTVFLTADRRRKTQALMTWNVTCTYSQFLASKNPSCCVSFSSFYNETIVPCPSCACGCHNIKNCVKSDSNLAHKAGINTPKKDNSPLLQCTHHMCPVRVHWHVKQNYKDYWRVKMAVTNFNYRMNYTDWTLVVQHPNLNNVTQVFSFGYKPLVPYQSVNDTGMFYGMKFYNDLLMEAGPSGNIQSEVLMQKDQNTFTFRQGWAFPRKVYFNGDECMLPPPDEYPSLPNSGHSDRCGPVRDNSV

>PbCOBL14

MESRKCKSTLFASFVCFTIFSYAVAYDPFDPKGSINIRWDVVSWTSDGYVAAVTISNNQMYRPVTSPGWTLGWTWAKKEVIWSMAGAQATDQGYCSKFKGNIPHSCSKNPSVVDLPPTAPYNQRFSDCCKGGVLESLGQDPSAAVSAFQLSVGHSGTSNKTVIPPKNFFLLGPGPGYTCSAATIVPPSVSYSPDGLRKTRAMMTWSLTCSYSQVLASQNPTCCVSLSSFYNPMITPCPSCACGCQNKNNCADNDPSRVVSLKTVVQKNASILQCTDHMCPIRVHWHVKANYRAYWRVRIAVTNFKYRMNYTQWTLVAQHPNLNKLANVSSFLYKPLIHYNTTGAGGHVESELILQKNTKEFTLKQGWAFPLMIYFNAPITKDAAKNPNDAFEDATSSSIQLMEGLHKKRLGVSELCHYMPVEL

>PbCOBL15

MMETSYLGTKMPWRSRTSFFHAMLVVFLVFSCFRVEICYGQDEDAVVAAPPPEQEDCDGIFLSYTFTSREKLLPHLKNVSAQAWAFKSEASILNAGSTELKAWKMYIGFQHREILVAAEGAQLVDGGDMPAEVGSKGAYFAGYPMTDLKTMIDTAGDYTQIQAKIAFKGTQFGMGEKSTPMPKSIAIVNDGFKCPAAKFKGKTAMSVCCKKDPKFKAKKVEKTKFMPRQNGDLSITYDILQTYTMNYLAQVTIDNVHPLGRLDHWNLTWEWMRGEFINTMRGAYTHKKDTSECLYGQAGKFYKDLDFSQVMNCEKRPVIADLPAERKDDPKVGKLPRCCRNGTILPGLMDKSQSQSIFQLQVYKIPPDDNRTAITPPQQWKISGVLNPTYRCGPPIRVDPTQFPDPSGLQATSDAIASWQVVCNITKPKVPRCCVSFSAYYSTSVVPCSTCACGCKTSETDKCNPRAPAMLLPAEALLVPFANRTVKAKAWAKLKHYDVPKKLPCPDNCGVSLNWHIDSDYSNGWTARLTLFNWGTDQFQDWYTAVKMNKAYQDYENVYSFNGTRMEKDVNNTILFTGLKGLNYLMGIKNGTDPKKNPMVPGKQQSVISFKKKHYHNIDIKAGQGFPTRVLFNGEECALPKVFPKNNAGHLKSNALLVLCIAIMSFLFMTDRFH

>PbCOBL16

MDSSSRKWPPILALNLLLIFTILPYSLAQSTAQAPPPASDSCNGVFLSYAYTTGAQLPPEVKDRSQQPYRFESVLTVLNNGLDDLKSWRVFVGFTNNEYLVSASNAVLADGGSIPGSVGNGTVFAGYPMTDLKTAVKTAGDLTQMQVQVKLIGTQFGVAPPKVPMPSNLTLANDGFVCPAAAMQGTHEMQVCCTVDTKFKTNITVDEKFLPRQKGDLTIMYDVISTQESKYTAQVTIANHNSLGRLDNWKLSWDWMEDEFIFSMKGAYPSVVDSSDCIFGKQGTFYQGLDFSTVLSCERRPTIIDLPPTKANDTLLGLVPYCCRNGTILPPTMDASKSVSSFQIQVFKMPPNLNRSQLSPPQNWGINGTLNPDYKCGPPVQVSPSQFPDQSGLPVNSTAVASWQVVCNITQPKGASPRCCVSFSAFYNESVIPCNTCACGCPRNIAQTCSTTAPAMLLPPESLLVPFDNRTVKAKAWAELKHLPDPNPTPCGDNCGVSINWHLYTDYSRGWSARVTVFNWDETAFVDWFAAVQLDKAGPGFEKAYSFNASHLELNGVNNTVFMQGLEGLNYLVAETDGANPQKDPRVPGKQQSVLSFTKKTTPGINLVGGDGFPTKVYFNGEECSLPTIYPSSAYRKSTSIIFSVLVMVVAFVGGFVKSQLPEDVHAIAGTCEGFRRILAVTGILLLFICKC

>FvCOBL1

MGFLLSKLTSFATVLVFWLCCFTFTSTDAYDPLDPSGNITIKWDIKSWTPDGYLAMVTIYNYQQYRHIQAPGWSLGWAWKNKEVIWDMVGGQATEQGDCSRFKTFPPHCCKKKPTIVDLLPGTPYNKQVSNCCKGGVLSSWMQDPFNAAASFQLSVGQAGTTNKKVKPPENFTLMAPGPGYTCGALKVVKPTKYFSPDKRRVTQAMMTWKLTCTYSQFLAQKTPACCVSLSSFYNDTVVPCPTCSCGCQNNLTHPGSCVDPKSPYLASVVSAADKNSPLVRCTHHMCPIRVHWHIKLNYREYWRVKVTITNFDYRRNYSDWNLVMQHPNFDNLTQVFSFNSKSLTPYGTFNDTAMLWGIKFYNDMLMQSGPVGNVQSELLFHKNPATFTFEKGWAFPRRISFNGDTCVMPPPDAYPYLPNAGSCQYISLLTLIMVCALTFAFAYAYA

>FvCOBL2

MRFFVSALFLLVIFSYAAAYDPLDPNGNITIRWDIMSWTPDGYVATVTINNFQMYRHIMSPGWTLGWTWAKKEVIWSAVGAQTTEQGDCSKFKGNIPHCCKKTPTVVDLLPGVPYNQQYQNCCKGGVVGAWGQDPSASVSAFQLSVGMAGTSNKTVKLPKNFTLLGPGPGYTCGKAKIVPSTIFFSPDHRRKTQALMTWNVTCTYSQLLARKNPSCCVSLSSFYNETIVPCPTCSCGCHNKDTCVKSNSKLLSMVGVNTPKKDNAPLLQCTHHMCPVRIHWHVKVNYKEYWRVKVTVTNFNYRMNYTLWTLVVQHPNLNNVTQVYSFDYKPLVPYESINDTGMFYGLKYFNDQLMEAGPFGNVQSEILLKKDINTFTFKEGWAFPRKVYFNGDECQMPPPDTYPFLPNSAPHQNLYSFSALVSILIFLLIAIW

>FvCOBL3

MGFLLSKLTSLVTVVLFGIGFCSFFTSTDAYDPLDPNGNITIKWDIISWTPDGYVAVVTLFNFQQYRHIQAPGWTVGWTWAKKEVIWNMVGGQATEQGDCSKFKPTPPHCCKKTPTIVDLLPGTPYNQQYSNCCKGGVLSSWMQDPSNAAASFQLSVGRSGTTNKTVRLPKNYTLMGPGPGYTCGVAKVVKPTKFFTADKRRVSQAMMTWNVTCTYSQFLAQRAPPCCVSLSSFYNDTVVPCPTCSCGCQNNITHPGSCVVPESPYLASVVSAADKNSPLVRCTSHMCPIRVHWHIKQNYKEYWRVKITITNFDYRRNYSDWNLVVQHPNFDNLTEVFSFNAKSLTPYGTINDTVMLWGIKFFNDMLVQSGPLGNVQSELLFRKNPATFTFDKGWAFPRRIYFNGDTCVMPPPDAFPHLPNAGFRQYVSLLTVIMISVSTFAFTYAYA

>FvCOBL4

MGGGGGGGGDVVCSFTFTLSLTMIIFFLSSISLSYGYDPLDPYGNVTITWDFLAQSNTDTYDIKVSIYNLQQYRHVEFPGWRLQWKWNKSEVIWDMRGAEAVEQGDCSAFKGSGAGDLPHCCLRRPVITDLLPGTPYNMQYKNCCKGGVLSSMNQDPSKYLSSFGMSIGNFHASPPGLNSSLDKLNLTMPTDFNLGIPGYTCGDAFLVPPTRYIDEKGQGRRWKQTLETWNITCTYSQFQASRAPKCCVSLSAFYNTTIVGCPRCSCGCQRLGGAKCIESGDKPPPSVLKLPTEQQAAIPPPLVTCSQHMCPIRVHWHVKQSYKEYWRVKITITNLNFLKNYTDWNLVVQHPNLKSIRQLFSFNYHPLTTYGNINDTGMFWGIKFYNDMILTSGNQGNAQSEMLLHKDEGTFTFREGWAFPRRISFNGDDCVMPPPDDYPTLPNIAATLNPYLLLVSFMFLAVAVPALF

>FvCOBL5

MELHNQADPAHRIHLEVVTCCSWLKITFLAMLFCVLVSPTAAYDPLDPTGNITIKWDVMSWTPDGYVAAVTMNNFQMYRQIANPGWTLGWTWAKKEVIWTMVGAQTTEQGDCSKFKGNTPHCCKKTPTVVDLLPGVPFNQQFSNCCKGGVMASWGQDPSASVSAFQVSVGQGGTSNKTVKLPKNFTLLGPGPGYTCGPAKVVPSTVFLTADRRRKTQALMTWNVTCTYSQLLASKNPSCCVSFSSFYNETVVPCPSCSCGCHNKKDCIKGDSKLLQKAGINTPRKDNTPLLQCTHHMCPIRVHWHVKQNYKDYWRVKISVTNFNYRLNYTEWTLVAQHPNLNNITQVFSFDYKPLVAYQSINDTGMFYGMKFYNDLLMEAGPLGNVQSEVLMQKDKNTFTFKQGWAFPRKVYFNGDECRLPPPDAYPSLPNSGHVDSVSILKMAASLLLIMFCIY

>FvCOBL6

MESQCLSATGSIAKLSSFTILLFFLVSSLTFTSTEAYDALDPLGNVTIKWDVISWTPDGYVAVVTMFNFQQYRHIQAPGWTLGWTWAKKEVIWSMVGAQTTEQGDCSRYKGNVPHCCKKDPTVVDLLPGTPYNQQIANCCKGGVMQSWIQDPGNAVSSFQVSVGAAGTTNKTVRLPKNFTLKAPGPGYSCGRAAIVKPTRYLTADKRRWTQAMMTWNITCTYSQFLAQKTPTCCVSLSSFYNDTIVNCPTCACGCENNSTNPGSCVEPDSPYLASAVAGNGKSANSPLVQCTSHMCPVRVHWHVKLNYKEYWRVKVTVTNFNYRMNYTLWNLVVQHPNFDNLTKIFSFNYKSLTPYAGLNDTAMLWGVKFYNDLLMQAGPLGNAQSELLFRKDTSTFTFEKGWAFPRRVYFNGDNCVMPPPDAYPWLPNASPRPTISLLSPVITIVASLVFLLAYA

>FvCOBL7

ITSPGWSLGWTWAKKEVIWSIVGAQPPIKETVPNSNPHCRKKTPTVINLLPGVPYNKQFNNCCISGQFVSVGLSGTSNKTVTVPKSFFLLGPGPYTCSAATILIAYSNLALIVCLVAYDPFDPNGSINIKWDVLSWTPDGYVAAVSISNNQMYRHITSPGWSLGWTWAKKEVIWSMVGAKVADQGDCSKFKGNTPLCCEKTPTVIDLLPGVPYNQQFTNCCKGGVLTSLGQDPSSAVSAFQMSVGLAGTSNKTVRPPKKFFLLGPGSGYTCSAANIVPSSVTLSSDHRSKTRAMMTWSLTCTYSQLIASKNPTCCVSMSSFYSPLITPCPSCACGCKDANKCVHLCRNDLRDSSLLQSKTSAKENAPQLLQCTEHKCPIRVHWHVKASYREYWRVRITITNFNFLMNYTQWTLVAQHPNLNKLANISSFLYKPLIQYNPTNDTGMFYGIKTVNDLIREAGPKGYIYTELILKKDKNTFTLDQGWAFPLRVYFNGDECLMPLPDLYPYLPNSAFHNSYPIFTLSSTILATLLVFVLLVLL

>FvCOBL8

MILQFYGELISTTIFGCKLGLVVLSNRNSFSGSIIQILIHRRVGDSLKMVITVSCHPLMHTHCSRISTTMTSLSTVLIHYLSSCKAYDPVDPIGHITIKWDIMNWTPDGYVVTYDPVDPNGNITIKWDIMNWTPDGYVGNIYIVTRLLLNQAVVKINNFQKYRNIQAPGWSLRWTWRKEEVIWSMVGEKPKEKGDCSRFKGSNPRSCEKNPTIVDAMPSTPYNQQIANCCKGGVLGSLVQDPANSVAAFQLTVGRAETTNRTVRLPKDFTLSAPGPNYSCGRATIVKPTRFFTPDLRRMTTTFMTWNVTCTCSQYAVQQVPTCCVSLSSFYKNTTIPCSTCSCSCQNNSNKCVELHSSSVVPVSDKSYVPVVQCTSHMCPIQISWQVTKDHKQYWLVKLKITNFNYKMNYLDWNLVIQHPNFDNLTRILRFNYKSLTPYAGINDTAMLWGVKFYNNILMQAGPLGTVESELIFQKHGINSNLSKDWAFPQRVYFNGDNCIMPSADAYLKHMMHLIPPQISRSDGTSLPGLLMVILPLLQLTTSKNIVTFKHQAGNWDGHGQRRSLYGAWLEEKPQRREIVQDSMELHHIRVKRPHRLSI

>FvCOBL9

MKRASRVLTPMFILFAIEFSNFTIAQDYGDDDSTPPAPPPAQDNCNGIFVSYDFISRHKIYPLLKNAEKQAWAFNSTATIINTGSYELKAWKIYVGFQHKEILVAANGGVLVNGEDFPAEVGTNGTYFSGSQQTDLKTSISTGGDFTQIQAIIQISGTQFGVKPSVTPMPKTIRLVNEGFKCPSPTKKKTSMTVCCVRNPKSKVATKAIKFLPRQKGDLSLSYDVIQAYGNNYLAQVTIESTSPLARLDHWNISWEWMRGEFIQTMKGAYTHKMDYLPCIYGAPGQYYQDMDFSKVMNCEKNPTIADLPRERSNDSEVGRIPYCCRNGSLLSPVMNKTQAKSVFQMQVFKLPPDLDRKTLYPPEKWKVSGVVSAEYKCGQPIRVDPTEFPDPSGLQASTLAIASWQVICNITRPQSKKNKCCVSFSSYYNESVIPCNTCACGCPDTKKCNPSARAMFLPPEALLVPFKNRSALAAAWAKIKHFHIPKPQPCGDNCGVSINWHVLSDYTDGWTARITLFNWMPINFEDWFAAVEMKKGGGRGYENAYSMNGTKLANMNNIIFLQGLKGLNFLVMQTNGTKKDSPAVPGKQQSVISFKKARTPGIQVAQGDGFPAKVYFNGEECALPTSFPSYGNRNHVNLIVVIFLSLLTFAM

>FvCOBL10

MGGGGGGGGGDVVCNIFTFTLSLMMIIFLSSISLSYAYDPLDPYGTVTITWDFLTQSDTDTYDIKVSIFNLQQYRHVELPGWRLKWTWNKSEVIWDMRGAEAVEQGDCSAFKGSGAGDLPHCCLKRPVITDLLPGTPYNMQYKNCCKGGVLSSMNQDPSKYLSSFGMSIGNFHANPAGSNKSFDKLNLTMPTDFSLGIPGYTCGDAFRVPPTRYSDEKGQGRRWKQTLETWNITCTYSQFQASRAPKCCVSLSAFYNTTIVGCPRCSCGCQRLGGAKCIESGDKPPPSVLKLPTEQQAAISPPLVTCSQHMCPIRVHWHVKQSYKEYWRVKITITNLNFLKNYSDWNLVVQHPNLKSVRQLFSFNYHPLTTYGNINDTGMFWGIKFYNDMILTSGNQGNAQSEMLLHKDEGIFTFREGWAFPRRISFNGDDCVMPPPDDYPTLPNIAATLKPYMLLVSFMFLAVASLALF

>FvCOBL11

MASTTRPFIFALALLTIYTSLPLSLSQPTAEAPSPAADSCNGIFISYSYTAGKKIPPTDPSNQPYRFESVLTVLNNGDVDLKSWKVFVGFQHHEYLASAENAVLADGTAYPGSVENGTVFAGFPQTDLKTAIKTAGDLNQMQVQVKLLGTQFGVAPPNVPMPANITLANDGFRCPKPSMHGKSVMEVCCVVDATVKTNITVDEEFLPRQNGDLSIMYDVIRTYGSSYVAQVTIANHNSLGRLDSWKLSWDWMAQEFINTMRGAYPSVVDSSDCIFGEQGQYWGDKDFAKVLSCEKRPTITDLPLEMTNNTELGLIPYCCRNGTILPTTMDASKSKSSFQMEVYKMPPNINRSVIIPPQNWAINGSLNPDYKCGPPVRVSPSQFPDASGLPVNSTAVASWQVVCNITQAKGASPRCCVSYSAYFNESVIPCKTCACGCPSNTAQTCSTTAPAMLLPPDSLLVPFVNRSVKAKAWADLYHLPNPNPTPCGDNCGVSINWHLLSDYSKGWTARVTLFNWDETSFADWFAAVQLDNASLGFEKMYSFNATLVETDGVNKTVFMQGLEGLNYLVGEVDGKNPAKDPRVPGKQQSVISFTKKLTPGINVAGKDGFPTKVFFNGEECSLPDIIPSSAYRRSSAIWMFPVLLTLAVFMLMQQ

>FvCOBL12

CVRVTEDPGCTTSFGACCCENFQLPKISSLSSTRLVIEMDMSERGEPTIVDATPSTPYSKRITDFCRGGVLGSLLPDPANSIAAFQLSVGEVGMNNGTIRLPKNFTLSVPGYSCGHAKIVKPTKFYTPDGRRLHGKLNVYILISWLRSFPPVVSHYLLSTITQPCPTIHVHVAAKTKPAIAEFIFTCPSLRQELCTAKCTNHMCPIQISWHVTEDHPKYWLVKLKITNLNYRMNYSDWNLVIQDPNFDNLTQIFRFNYKSLTLYAT

>FvCOBL13

METSSSMKIPWSLLFLLFLGVFAARVEVSYGQKAAKLGAGAGASGGGDDGGDGDKPAPPPEQEDCDGIFLTYTFISREKEYPRKKNVSAQAWAFTSEASILNAGTETLKAWKMYVGFQNREILVSADGAVLVDGSDFPASVGKNGTYLSGDQQTDLKNSVDTAGDIEQIQAKIAFKGTQFGLPAKAVPMPKTIRLVNDGYKCPAPRIRGKTNMFVCCVPDPKFKVKKDDKDSKFLPRQNGDLSITYDVLDSYSNNYMAQVTIDNLHPLGRLDHWNLTWEWQKNEFIQTMRGAYTHKKDMSECLYGPAGKAYKDMDFSKVMNCEKKPTITDLPPTMKDDEKVGKLPKCCRNGTILPTIMNETQSQAQFQLQVFKLPPDDNRTALTPPQKWSIYGPVNPNYVCGPPIRVAPTLFPDPSGLQSTTAAVASWQVVCNITKPKVPRCCVSFSGYFSESIVPCNTCACGCKEDENPNCDPRAKAMLLPAEALLVPFANRTQKAKAWAKIKHFDIPKKLPCGDNCGVSLNWHIDSDYQSGWTARLTLFNWGKEPFQDWFTAVEMNKAFLDYENVYSFNGTRMAKPKNTILFQGLKGLNYLIEMRNATNPKDPRVPGKQQSVISFKKHHYHGINIKAGEGFPSRVLFNGEECSLPKRFPNGADHQASSNVLLILFVAIASFVFMTDRFH

>PmCOBL1

MVTLGMKIPWTIQMSSFHAMLVVFLVLSSFRVEICYGQAGDGDGDGAVAAPPPEQEDCDGIFLSYQFTSREKELPRLKNVSAQAWAFKSEATILNAGSTELKAWKMYIGFQHREILVEAEGALLVDGGDLPVQLGTKGATFAGNPMTDLKTMIDTAGDYTQIQSKIKFKGTQFGLGTKATPMPKSISLVNDGFKCPNARIRGKSTMFVCCKKDPKFKAKKIEKTKFMPKQKGDLSITYDVLQTFANNYLAQVTIENNHPLGRLDHWNITWEWMKGEFINNMRGAYTHKKDSTECLYGMAGKFYKDLDFSQVMNCEKRPVITDLPMDRKDDPKVGKLPNCCRNGTILPGLMDKSQSQSIFQLQVFKLPPDDNRTALTPPQKWKINGALNPSYKCGPPIRIDPTQFPDPSGLQATSASVASWQVVCNITKPSVPRCCVSFSAYYSDSVVPCSTCACGCKITDTGKCSPREPMMLLPMEALLVPFANRTEKAKAWAKIKHYDVPKKLPCPDNCGVSLNWHIDSDYSNGWTARLTLFNWGNDPFQDWYTAVKMNKAYADYENVYSFNGTRMEKEVNSTIMFTGLKGLNYLIGIKNGSDPNKNPMVPGKQQSVISFKKKHFHNIDIKAGEGFPTRVLFNGEECAIPKRFPRNNAQHLNSNALMVLCIAILTFLFMTDRFH

>PmCOBL2

MEPRYLSDTGSIVKLSSFAILLVFWLSCFSFTSTEAYDALDPTGNITIKWDVISWTPDGYVAVVTMFNFQQYRHIQTPGWTLGWTWAKKEVIWSMVGAQTTEQGDCSRYKGNVPHCCKKDPTVVDLLPGTPYNQQIANCCKGGVMNSWIQDPMNAISSFQISVGAAGTTNKTVRMPKNFTLKAPGPGYSCGIAKIVKPTRFLTADKRRWTQAMMTWNVTCTYSQFLAQKTPTCCVSLSSFYNETIVSCPTCACGCQNNATDPGSCVEPNNPYLASAVSGPGKSTNAPLVQCTSHMCPVRVHWHVKLNYKEYWRVKVTITNFNYRMNYTLWNLVVQHPNFDNLTKIFSFNYKSLTPYAGLNDTAMLWGVKFYNDLLTQAGPLGNVQSELLFRKDASTFTFEKGWAFPRRIYFNGDNCVMPPPDAYPWLPNSSPKQVISILHPMVTIFVSLLFLLAYA

>PmCOBL3

MEFKNQANPTPKVHLEVETCCSWSWLKFTILAVLCCMVLSPMAAYDPLDPTGNITIKWDVMSWTPDGYVAAVTMNNFQMYRQIISPGWTLGWSWQKKEVIWTMVGAQTTEQGDCSKFKAAIPHCCKKTPTVVDLLPGVPYNQQFTNCCKGGVLASWGQDPTASVSAFQVSVGQAGTSNKTVKLPKNFTLLGPGPGYTCGPAKVVPSTVFLTTDRRRKTQALMTWNVTCTYSQFLASKNPSCCVSFSSFYNETIVPCPSCACGCHNKKNCIKSDSKLAHKTGINTPKKDNTPLLQCTHHMCPIRVHWHVKLNYKAYWRVKISITNFNYRMNYTEWTLVAQHPNLNNVTQVFSFDYKPLLPYESINDTGMFYGMKFYNDLLMEAGPSGNVQSEVLLQKDQNTFTFKQGWAFPRRVYFNGDECMLPPPDAYPMLPNSAHVNSISIFKMAASVLLILFSICFSLS

>PmCOBL4

MTFDKYMCLVLSAVSFILTIPHAAAYDPLDPNGNITLKWDVISWTSDGYVAVVTTNNFQMYRHIMSPGWTLGWVWAKKEVIWSMVGAQAVDQGDCSKFKGNIPHCCKKNPTVVDLLPGVPYNQQIANCCKAGVVASWGQDPSAAVSSFQLSVGRSGTSNKIVRLPKNFTLLGPGPGYTCSQAKIVRSTVFNSPDGRRKTQALMTWNVICTYSQLLASRYPTCCVSMSSFYNSTITPCPTCACGCRDKKKCIVSDSKLSSVVGTHTPTKDNTPLLQCTQHMCPIRVHWHVKTNYKQYWRVKITITNFNYQLNYTQWTLVVEHPNLNHITEVFSFVYKPLTPYQSKNDTGLFYGIKFYNDLLKEAGPEGNVQSELILEKNANTFTFKEGWGFPRKVYFNGDECMMPQPDEFPGLPNAAHTNLITVPKLALFWLLMFLALP

>PmCOBL5

MEIISKCIRSAFLVSVMFLTIVSRAVAYDSFDPNGRINIKWDVLSWTPDGYVAAVTIINNQMYRHITSPGWTLGWTWAKKEVIWSMVGAQATAQGDCSKFKANIPHSCKKTPTVVDLLPGVPMNRRFSDCCKSGVMASWGQDPSAAVSAFQLSVGHSGTSNKTVTPPKNFYLLGPGPGYTCSAATIVPPSVSFSLDGRRTTQAMMTWALDCTYSQLLVSENPTCCVSLSSFYNPMITPCPSCACGCKDVNNNCINDPKDSRVLNKKALAKEDASMLQCTDHRCPIRVHWHVKANYRAYWRVKITITNFNYLMNYTQWTLVAQHPNFNKLANVSSFVYKPVIQYGSINDTGMFYGIKNHSDLLMEAGPEGYVQTELLIGKDMKALTLDQGWAFPFKLYFNGDECKMPLPDIYPTLPNSADANPISSSTLATTLLLSALLVFLCSCH

>PmCOBL6

MGWIWQQNEVIWSMNGAFATEQGNCSNYKTDIPHSCKKDPEILDLMPDASSDNKSEDCCRSGVLDALAINPSKSFSSFGIKVGNLGGGPFSGLPPLNLTLNAPGPGYTCGPLKSVDPTVSLDFGGRRHRQVFRTWKAICTYSSFLASGTPVCCVSLSSFYNPEITSCPKCSCGCREVTDKNFGTCLRPMYPFPMMSLTGDNSGANFMGALDPVKCTDHMCPIRVHWHVKTSYVDHWRIKLTITNYNYQRNFSNWNVLVQHPGLSMNPITYSFNSTLLQPGFRDAVALFWGIAFVNEELIATDEDGVGSVSTEILLEKDSESFTFKNGWAFPRRVYFNGENCEMSLPDTFPMLPNGTSKTQPPTHWLFLLLIFFISQTLLLHAL

>PmCOBL7

MRFFVSALFFLVIFSYADAFDPLDPNGNITIKWDIMSWTPDGYVAVVTLNNFQMYRHIMSPGWTLGWTWARREVIWSAVGAQATEQGDCSRFKGNIPHCCKRNPTFVDLLPSVPYNQQFTNCCKGGVLGAWGQDPSAAVSAFQLSVGSAGTSNRTVKLPRNFTLLGPGPGHTCGRARVVPPTIFLTPDRRRKTQALMTWNVTCTYSQILARKYPSCCVSLSSFYNDTVVPCPPCTCGCQTKDNCVKSDSKILSMVGVNTPKKDNKPLLQCTRHMCPVRVHWHVQRNYKDYWRVKVSITNFNYRMNYTLWTLVVQHPNLNNVTQVFSFDYKPLVPYESINDTGMFYGLKFFNDQLMEAGPFGNVQSEMLLRKDKNTFTLKEGWAFPHKVYFNGDECQLPPPDAYPFLPNSAYQNLLSFSTFISSFIFFLIAIW

>PmCOBL8

MGPLFLPMIRSVSKLISFTILLLFGLACTSFTATEAYDPLDPNGNITIKWDIISWTPDGYVAVVTIYNFQKYRHIQAPGWSLQWTWAKKEVIWNMVGGQATEQGDCSKFKTTIPHCCKKNPTVVDLLPGTPYNQQITNCCKGGVLTSWVQDPMNAVGSFQLSVGQAGTTNKTVRAPKNFTLNAPGPGYTCGRANIVKPTKFVTPDKRRVTQAMMTWNVTCTYSQFLAQKTPTCCVSLSSFYNDTVVPCPMCSCGCQSNATHPGSCVEPDSPYLASVVSASSKNSYTPLVRCTNHMCPIRVHWHVKLNYKEYWRVKVTVTNFNYRMNYSDWNLVVQHPNFDNLTQSFSYNYKSITPYATINDTAMLWGLKFYNDLLMQAGPLGNVQSELLFRKDQATFTFDKGWAFPRRIYFNGDNCVMPPPDAYPWLPNSGFRQYTSLLTLIMTSLSTAALMYVHA

>PmCOBL9

MKRAWWVVITLMFALFGFAFSVTSAQDNGDGGDQQPMAPTPMQQDCDGIYMSYDFESRRKIYPFLKNAEKQAWAFKSTAHIVNTGTYELKAWKIYVGFQHKEILVGATGAVLMNGDDFPMDVGNGTYLSGSGQTDLKTSISTAGDYTQIQAKIQFSGTMFGVKPSGVPMPKTIRLVNDGYKCPSPTNRKGSMYVCCVRNPKFKANVTKTKFLPRQKGDLTIAYDVIQAYENNYLAQVTMENCSPLGRLDHWNLTWEWMRGEFIYNMKGAYPRTIDYLNCIYGDAGKYYQQMDFSKVLNCEKKPVIGDLPREKANDTQVGKIPNCCRNEYKCGQPIRVDPMQFPDPSGLQATSLAIASWQIVCNITRAKTRKPKCCVSFSAYYNESVIPCNTCACGCSDTKKCNPKAPMLLLPPETLLVPFENRTKKAIAWASIKHHHVPKPLPCGDHCSVSVNWHLLSDYKDGWTARITLFNWDKTNFEDWFTAVQLKKATAGYEKAYSFNGTKIPKLDNIIFLQGLKGLNFLVAQKNGTKPEKDPKVPGKQQSVISFKKKHTPDIEVAKGDGFPTRVFFNGEECSLPTQLPLSHGNCLHVNFVVAIFLSLLSFVM

>PmCOBL10

MDFSSMSCPLIFTLSFVLIFTALPSSFSQPTADAPMPMSDSCNGVFLSYAYTTGAKLPPELKSNPKRQPYRFESVLTVLNNGLEDLKSWRVFVGFKNDEYLVSASNAVLADGTSMPGSVGNGTVLAGFPMTDLKTAIKTAGDLTQMEVQVKLVGTQFGVAPPKVPLPSNISLANDGFVCLNAVQGTNEMHVCCTVDTKFKTNITVDGKFLPRQNGDLTIMYDVTNTQDSNYWAQVTIANHNPLGRLDNWKLSWDWMADEFIFAMKGAYPSIVDSSDCIFGRQSTYYKDLDFSTVLNCEKRPTIIDLPPTKANDTLLGLVPNCCRNGTILPRSMDPSKSMSSFQIQVFKMPPDLNRSQFTPPQNWAINGTLNPDYECGPPVRVSPSQFPDRSGLPVNSSAVASWQVVCNITQLKGASPRCCVSFSAFYNDSVIPCNTCACGCPSNTARTCSTTAPMMLLPPETLLVPFENRTVKAKAWAELKHLPVPNPIPCSDNCGVSINWHLYTDYSRGWSARVTLFNWDETSFVDWFAAVQMDKAGPGFEKMYSFNGSTLELNGVNNTVFMQGLEGLNYLVAETDAANPQKDPRVPGKQQSVISFTKKKTPGINVIGGDGFPTKVYFNGEECSLPKIYPSSGNRKSTPIMFSVLLMVVAFMVM

>PmCOBL11

MMSPPFLFGAQRSNAVLFVIFISSLVTSFSLSYGYDSLDPYANITITWDFRLQTGSTYDIKVSIYNFQQFRHLDRPGWKLSWIWKDDDQVIWDMWGAEAMEQGNCSKFRSSPQLPHCCKKQPVIIDFLPGAPYNKQFSNCCKGGELSSMIQDSSKFLSAFQMNVGFGKTNNITEVMPTNFTLGIPGYTCGDAFLVLPTRSSPDGRRWVQTLETWNVTCMYSQFRASPSPKCCVSLSGEKSPHLLELPRARENEEVPPLVTCSQHMCPIRVHWHVKQSYKEYWRVKITITNLNFVKNYSSWSLVVQHPNLRSVTQLFSFNYHPLNAYGNINDTGMFWGIKSYNDMLLASGQSGNAQSEMLLHKDPGIFTFREGWTFPRRISFNGDECVMPPPDEYPTLPNSATTSRPSLVFFSFLILAFVF

>MdCOBL1

MRTLVSVLFFLAIFSCAAAYDPLDPNGNITVKWDIMSWTPDGYVAAVTVNNFQMYRHIMSPGWTLGWTWARREVIWAAVGAEATEQGDCSRFKGNVPHCCKKTPTFVDLLPGVPYNQQFTNCCKGGVLSAWGQDPSTAVSSFQLSVGSAGTTNRTVRLPRNFTLLGPGTGYTCGKARIVPPTIFLTSAGRRRTQALSKFLRVT

>MdCOBL2

MKFTFLAVLYCLILSPAAAYDPLDPTGNITIRWDVMSWTADGYVAAVTMNNFQMYRQIPSPGWTLGWSWAKKEVIWTMVGAQTTEQGDCSKFKATIPHCCKKTPTVVDLLPGVPYNQQFSNCCKGGVLASWGQDPSAAVSGFQVSVGQAGTSNKTVKLPKNFTLLGPGPGYTCGPAKVVPSTVFLTADRRRKTQALMTWNVTCTYSQFLASKNPSCCVSFSSFYNETIVPCPSCACGCHNKKNCVMSDSKLAHKAGINTPKKDNSPLLQCTHHMCPIRVHWHVKQNYKDYWRVKMAVTNFNYRMNYTEWTLVVQHPNLNNVTQVFSFGYKPLVPYQSVNDTGMFYGMKFYNDLLMEAGPSGNIQSEVLMQKDQNTFTFKQGWAFPRKVYFNGDECMLPPPDAYPSLPNSGHVRATDVVLCAIIRYRL

>MdCOBL3

MEFENQANQTHNIRLTCCSWSWMKFTFLAVLYCLVLSPASAYDPLDPTGNITIKWDVMSWTPDGYVAAVTMNNFQMYRQISNPGWTLTWSWAKKEVIWTMVGAQTTEQGDCSKFKAAIPHCCKKTPTVVDLLPGVPYNQQFTNCCKGGVLASWGQDPLAAVSGFQVSVGQAGTSNKTVKLPKNFTLLGPGPGYTCGPAKVVPSTVFLTTDRRRKTQALMTWNVTCTYSQFLASKHPSCCVSFSSFYNETIVPCPSCACGCHNKNNCLKSDSVLAHKTGINTPKKDNSPLLQCTHHMCPIRVHWHVKQNYKDYWRVKMSVTNFNYRMNYTEWTLVAQHPNLNNVTQVFSFDYKPLIPYQSVNDTGMFYGMKFYNDLLMEAGPLGNIQSEVLMRKDQNTFTFKQGWAFPRRVYFNGDECMLPPPDAYPSLPNSGHVGVIWPCV

>MdCOBL4

MGLETRTVAGLGVGWRRLVAGEVRLGKEVSGRPWFWASGGTEGAYDPLDPNGNITIKWDILSWTPDGYVASVTLFNFQKYRHIQAPGWLLGWTWAKKEIIWNMVGGETTDQGDCSKFKTTIPHCCDKTPTIVDLLPGTPYNQQYANCCKGGVLSSWVQDPVNAAASFQISVGQAGTTNKTVRLPKNFTLNTPGPGYTCGPANVVKPTQFISPDKRRVTQARIVSASNKHSYTPLVCTNHMCPIRVHWHVKENYQQYWRVKITVTNFNYRMNYSDWNLVVQHPNFDNLTQIFSYNYKSITPYGRINDTAMLWGVKFYNDVLMQSGPLGNVQSELLFQKGEAFTFDKGWAFPRRVYFNGDNCVMPPPDAYPRLPNSGFRQHVSLLTLIMTFLSTVAVMYAYV

>MdCOBL5

MPWRSRTSFFHAMLVVFLVFSCFRVEICYGQDEDAVVAAPPPEQEDCDGIFLSYTFTSREKLLPHLKNVSAQAWAFKSEASILNAGSTELKAWKMYIGFQHREILVAAEGAQLVDGGDMPAEVGSKGAYFAGYPMTDLKTMIDTAGDYTQIQAKIAFKGTQFGMGEKSTPMPKSIAIINDGFKCPAAKFKGKTAMSVCCKKDPKFKAKKVEKTKFMPRQNGDLSITYDILQTYTMNYLAQVTIDNVHPLGRLDHWNLTWEWMRGEFINTMRGAYTHKKDTSECLYGQAGKFYKDLDFSQVMNCEKRPVIADLPAERKDDPKVGKLPRCCRNGTILPGLMDKSQSQSIFQLQVYKIPPDDNRTAITPPQQWKISGVLNPTYRCGPPIRVDPTQFPDPSGLQATSDAIASWQVVCNITKPKVPRCCVSFSAYYSTSVVPCSTCACGCKTSETDKCNPRAPAMLLPAEALLVPFANRTVKAKAWAKLKHYDVPKKLPCPDNCGVSLNWHIDSDYSNGWTARLTLFNWGTDQFQDWYTAVKMNKAYQDYENVYSFNGTRMEKDVNNTILFTGLKGLNYLVGIKNGTDPKKNPMVPGKQQSVISFKKKHYHNIDIKAGQGFPTRVLFNGEECALPKVFPKNNAGHLKSNALLVLCIAIMSFLFMTDRFH

>MdCOBL6

MRWWIAALMFAFGFSLAVAQDYGDDQQQQPAAPPPSQEDCDGIYLSYNFLSRRRIYPFLKNAEKQAWAFNATATIVNTGTYELKWKMYVGFQHKEILVGAGGAVLMDGDDFPADVGNGTTLSGAGQADLKTAIGTGGDYTKIQANIQISGTMFGVKPPGNPMPKTIRLVNDGYKCPAPTNRKTTMYVCCVRNPKYKSIVTKTKFLPRQKGDLTIAYDVIQAYENNYLAQVTMENKSPLGRLDHWNLTWEWMRGEFIYSMKGAYPRSIDYLNCIYGAAGQYYQQMDFKVLNCKKNPVIGDLPREKANDTQVGKIPNCCRNGSILPAIMDQSKTKAAFQMQVFKVPPDLNRTALYPPEKFKVEGVLNPEYKCRQPIRVDPAQFPDPSGIQSTSLAIASWQIICNITRAKTRKPKCCVSFSAYYNESVIPCRTCACGCTDTKKCNPKAPAILLPPETLLVPFENRTKKAIAWASIKHHHVPKPLPCGNNCPVSVNWHVLSDYKDGWTARMTLFNWANMNFEDWFAAVQLKKASPGYENAYSFNGTKLPMLEDIIFLQGLRGLTFLVAETNGSSPKAPRVPGKQQSVISFKKKRTPDIEVAKRSRKCLKIFAAVTGIFFIITLVVLVVLFVTILKPKDPSVFTEPVSLEGFEFVGFPVIKLNISIRILVTVKNPNYGGFKYENSTAHISYHGNVVAEAPIKDDTVPARSTHNMTTVLSILADKLVTDSHFLGELVVGVLNFTSETTLHGKVNLWKIFKMKATSYTDCNISITIGTQSDSVCKSRVKLNK

>MdCOBL7

MARMSLRVSFESGFDPLDPYANITVTWDFLQQSESTSDIKVSIYNFQQFRHVDRPGWKLGWRWQGDEVIWNMWGAEAIYQGNCSRFKGAPERPHCCEKEPVIIDLLPGTPFNKQFSNCCKGGELSSMVQDSSKFLSAFRMNVGAFSNNSTNINMPDNFTLGLPGYTCGAPVQVQPTKTSQDGRRWTETLETWNVTCMYSQFRASPSPKCCVSLSSFYNSTIAKCPRCSCGCQGLPGAKCVKPGDELQLPGERETEEPSPLVTCTRHMCPIGIHWHVKESYKEYWRVKITITNYNFVKNYSDWTLVVQHPNLQSLTQLFSFNYHPLGQYGNINDTGMFWGIKYYNDMLLSTGKNGNVQTEMRGMDSQENFIQRG

>MdCOBL8

MDSGSRNWPPIMALNLLIILTIMPFSLAQPTAAAPAPASDSCNGVFLSYAYTAGPQLPPEVKDPKQQPYKFESVLTVLNNGLDDLKSWRVFVGFTNNEYLVSASNAVLADGTSIPGVGNGTVFAGYPMTDLKTAVKTAGDLAQMQVQVKLIGTQFGVAPPKVPMPSNITLANDGFVCPAAAMQGTHEMQVCCTVDANFKTNITVDDEFLPRQKGDLTMMYDVISTRATDYTAQVTIANHNSLGRLDNWKLSWDWMEDEFIFSTKGAYPSVVDSSDCIFGKQGTFYENLDFSTVLNCERRPTIIDLPPTKANDTLLGLVPYCCRNGTILPPTMDASKSVSSFQMQVFKMPPNLNRSQLSPPQNWGIKGTLNPDYKCGPPVRVSPSQFPDRSGLPVNISAVASWQVVCNITQPRGAIPSCCVSFSAFYNESVVPCNTCACGCPSNTVRTCSTTAPAMLLPPESLLVPFDNRTVKAKSWADIKHLPVPNPTPCGDNCGISINWHLYTDYSRGWSARVTVFNWDETAFVDWFAAVQLDKAGPGFEKAYSFNASHLEINGVNNTVFMQGKGLNYLVAETDGANPKKDPVPGKQQSVLSFTKKITPGINLVGGDGFPTKVYFNGEECALPTAYPSSGYRKRTSLIFSVLLRDEAMLLVATADVYILSVVLIDACSTGRFCEIPTTRRRACHCRYLKEVFRRILVVAGILLLFICKYREDRHTAK

>MdCOBL9

MAKLTSFTILLVFLLSCFTFTSTEAYDALDPNGNITIKWDVISWTPDGYVAVVTMFNFQQYRHIQAPGWTLGWTWAKKEVIWSMVGAQATEQGDCSKYKGNVPHCCKKDPTVVDLLPGTPYNQQIANCCKGGVMQSWIQDPDNAISSFQISVGAAGTTNKTVRLPKNFTLKAPGPGYSCGIAKIVKPTRFLTSDKRRWTQAMMTWNVTCTYSQFLAQKTPTCCVSLSSFYNQTIVSCPTCACGCQNNATDPGSCVEPNNPYLASAVSGSGKSTNTPLVQCTSHMCPIRVHWHVKLNYKEYWRVKVTITNFNYRMNYTLWNLVVQHPNFDNLTKIFSFNYKSLTPYVGLNDTAMLWGVKFYNDFLSQAGPLGNVQSELLFRKDTSTFTFEKGWAFPRRIYFNGDNCVMPPPDSYPWLPNASPKRIISILSPVMTAFASLVFLLAYA

>MdCOBL10

MSLIHSCLWPSALVSMLLIAMCSLSGMNTSHYIDHKSMCCFLIPFLDCYDPLDPNGNFTVTFDTYDWADDGYLLGWVWQQNEVIWSITGAFANDQGNCSAYQTDIPHSCKKNPEIVDLTPDALLDNKSEDCCRGGVLDALAINPSKSLSSFGIKVGNLGGYPFNGSPPLNLTLKAPGLGYTCGPLLNVDPTVSTDFGGRRQRPVFRTWKATCTYSSFLASDSPVCCVSLSSFYNSEITSCPKCSCGCRPVTDKKMDTCIRCTVNYFYCIITLRDPNFQVNLNSTNPSFLYLFPLYDNRQAYPLGTSVEYDGDGLDTVKCTDHMCPIRVHWHVKTNYLTHWRVKLTITNYNYMKNFSNWNVLVQHPGLSKEARTYSFNSTILPTRFRDAAALYWGIPYVNEELIATTEDGVGSVSTEMLLEKDSNSFTLKNGWGFPRRLYFNGEICATERVGSSASVYAGLMVMSGVGAVPARVVHFVAAVVGRVRVSHFRFRSSYSAPTLYLSRMLQAHESLYIESTRLLPVLDQLDPAKHSLGYLYILEACSFGSISKEQASTLVFPIAKFINSCVEEQIRLQPDKFDLRTPANKAKSWLLALQPSAAVAVHMQLSMLQTFQILFALFGRLRLFDEWFERERMEIESEMKQQSKFRRICVFCESSQGKKSNYQDVAIELGKELMLNLISNRQTASKVSQTPTPASNAATSPLDMGKTGVPPAMPIGPTVSTAPASSTSSACHTLETWRTKLKMYNSVKPLKGFGYRAIGEEGFGWNIGLMGSMVERGQTVLEEVASQLPSETPIDEVFPFEDAGFQIMTDTLDQTLDCKHGNVHRGLGKARIRTRLPYPSSREHKRSNR

>MdCOBL11

MNNFQMYRQIPSPGWTLGWSWAKKEVIWTMVGAQTTEQGDCSKFKATIPHCCKKTPTVVDLLPGVPYNQQFSNCCKGGVLASWGQDPSAAVSGFQVSVGQAGTSNKTVKLPKNFTLLGPGPGYTCGPAKVVPSTVFLTADRRRKTQALMTWNVTCTYSQFLASKNPSCCVSFSSFYNETIVPCPSCACGCHNKKNCVMSDSKLAHKAGINTPKKDNSPLLQCTHHMCPIRVHWHVKQNYKDYWRVKMAVTNFNYRMNYTEWTLVVQHPNLNNVTQVFSFGYKPLVPYQSGMKFYNDLLMEAGPSGNIQSEVLMQKDQNTFTFKQGWAFPRKVYFNGDECMLPPPDAYPSLPNSGHVRATDVVLCAIIRYRL

>MdCOBL12

MASTLTSSSLTAKLQKSSLLDSSFHGAPMVRLQLAKAAPANNGGLSVSMSANGLTPSYDLSAFKFDPIKESIVSREMTRRYMTDMITYADTDVVVVGAGSAGLSCAYELSKNPDVQVAIIEQSVSPGGGAWLGGQLFSAMVVRKPAHLFLNELGIDYDEQDNYVVIKHAALFTSTIMSKLLARPNVKLFNAVAAEDLIIKGGRVGGVVTNWALVSMNHDTQSCMDPNVMEAKVVVSSCGHDGPMGATGVKRLRSVGMIESVPGMKALDMNAAEDAIVKLTREIVPGMIVTGMEVAEIDGSPRMPLLCLADFIQPLQAIAITFSQNGDPVVAQREAVVRAPGIRRRPASGASTSAAAAPGEESVEGSDEDVEGEYDAKASKKREKKRQEREAQRQAEQAARESRVTKQDRYAEMRRRKDEEREAQERQLEEEAKAQKAREEEAAALEFEKWKGEFSIDAEGTENEVQDGQDLLSDFVEYIKKHKCIPLEDLAAESKLRTQECIRITNLESMGRLSGVMDDRGKYIYISQEEMQAVADYIKRQGRAMLVVFLVFSCFRVEICYGQDEDAVVAAPPPEQEDCDGIFLSYTFTSREKELPHTKNSAQAWAFKSEATILNAGSTELKAWKMYIGFQHREILVAAEGAQMVDGGDLPVDVGTKGAYFAGYPMTDLKTMIDTAGDYTQIQAKIAFKGTQFGMGEKSTPMPKTISIVNDGFKCPAAKFKGKTAMSVCCKKDPKFKAKKVEKTKFMPRQNGDLSITYDVMQTYTMNYLAQVTIDNVHPLGRLDHWNLTWEWMRGEFINTMRGAYTHKKDTSACLYGQAGKFYKDLDFSQVMNCEKKPVITDLPAERKDDPKVGKLPSCCRNGTILPSLMDAQSQSIFQLQVFKIPPDDNRTAITPPQQWKISGVLNPTYRCGPPIRVDPTQFPDPSGLQATSDAIASWQVVCNITKTPVPRCCVSFSAYYSTSVIPCSTCACGCKTSETNKCNPKASAMLLPAEALLVPFANRTEKAKAWAKIKHYDVPKKLPCPDNCGVSLNWHIDSDYSNGWTARLTLFNWGTDQFQDWYTAVKMNKAYQDYENVYSFNGTRMEKEVNNTILFTGLKGLNYLVAIKNGTDPKKNPMIPGKQQSVISFKKKHYHNIDIKAGQGFPTRVLFNGEECALPKVFPKNNAGHLKSNALLVLCAIMSFLFMTNRFH

>MdCOBL13

MEFENQANPTHKICLTCSWSWMKFTFLAVLYCLILSPAVTMNNFQMYRQIPSPGWTLGWSWAKKEVIWTMVGAQTTEQGDCSKFKATIPHCCKKTPTVVDLLPGVPYNQQFSNCCKGGVLASWGQDPSAAVSGFQVSVGQAGTSNKTVKLPKNFTLLGPGPGYTCGPAKVVPSTVFLTADRRRKTQALMTWNVTCTYSQFLASKNPSCCVSFSSFYNETIVPCPSCACGCHNKKNCVMSDSKLAHKAGINTPKKDNSPLLQCTHHMCPIRVHWHVKQNYKDYWRVKMAVTNFNYRMNYTEWTLVVQHPNLNNVTQVFSFGYKPLVPYQSVNDTGMFYGMKFYNDLLMEAGPSGNIQSEVLMQKDQNTFTFKQGWAFPRKVYFNGDECMLPPPDAYPSLPNSGHVRATDVVLCAIIRYRL

>MdCOBL14

MFVVAYDPFDPKGSINIRWDVVSWTSDGYVAAVTISNNQMYRPVTSPGWTLGWTWAKKEVIWSMAGAQATDQGYCSKFKGNIPHSCSKIPSVVDLPPTAPYNQRFSDCCKGGTSNTRPPKNFFLLGPGPGYTCSAATIVPPSVSYSPDGLRKTRAMSKFYRSNKMCPKCHIPSFDISSHHVVYKLLRQQNKKRDNDPSSVVSLKTVVQNASILCTDHMCPIRVHWHVKANYREYWRVRIAVTNFKYRMNYTQWTLVAQHPNLNKLANVSSFLYKPLIHYDTNDTGMFYGIKNLNNLLMEAGTDGHVESELILQKNTKEFTLKQGWAFPLMIYFNGDECKMPLPDIYPLPNTNSNPIPSSTLATLLLSALLLVFFSSRH

>MdCOBL15

MRAKAVAFRTWKAKQKSREIRETERIGHVVPSMRQPLAAYDPLDPNGNITVKWDIMSWTPDGYVAVVTMNNFQTYRHIMSPGWTLGWMWARKEVIWAAVGAEATKQGDCSWFKGNIPHCCKKTPTVVDLLPGAPYNQQFTNCCKGGVVSAWGQDPSTAVSSFQLSVGSAGTTKRTVRPPKNFTLLGPGPGYTCGRARVVPSTIFLSPDRRRRTQALMTWNVTCTYSQLLAKKYPSCCVSLSSFYNSTVVPCPSCTCGCHDKSNCVQSGSKIVSTVEDDAKSNVQPLPQCTRHMCPVRVHWHVMLNYDYWVKVSITNFNYRMNYTLWTLVIQHPNFINVTQVFSFDYKPLVAYDSINDTGMFYGLKFYNDHLMEAGKFGHVQEMLLKKDKNTFTLREGWAFPRKVYFNGDEQLPPPDVYPFLPNSAHETLLSFSPLISSFTFLFIVIW

>MdCOBL16

MDSSSRNWPPILALNLLLIFMILPSLAQSTAQAPPPASDSCNGVFLSYAYTTGAQLPPEVKDPSQQPYRFESVLTVLNNGLDDLKSWRVFVGFTNNEYLVSASNAVLADGSSIPGSVGNGTVFAGYPMTDLKTAVKTAGDLTQMQVQVKLIGTQFGVAPPKVPMPSNLTLANDGFVCPAAAMQELVEFDERMLVIWIVLSGTHEMQVCCTVDTKFKTNITVDEEFLPRQKGDLTIMYDVISTQESKYTAQVTIANHNSLGRLDNWKLSWDWMEDEFIFSMKGAYPSVVDSSDCIFDFSTVLNCERRPTIIDLPPTKANDTLLGLVPYCCRNGTILPPTMDASKSVSSFQIQVFKMPPNLNRSQLSPPQNWGINGTLNPDYKCGPPVQVSPSQFPDRSGLPVNSTAVASWQVVCNITQPKGASPRCCVSFSAFYNESVIPCNTCACGCPSNIARTCSTTAPAMLLPPESLLVPFDNRTVKAKAWAELKHLPDPNPTPCGDNCGVSINWHLYTDYSRGWSARVTVFNWDETAFVDWFAAVQLDKAGPGFEKAYSFNASHLELNGVNNTVFMQGLEGLNYLVAETDGANPQKDPRVPGKQQSVISFTKKTTPGINLVGGDGFPTKVYFNGEECSLPTIYPSSAYRKSTSIIFSLLVMVVAFVGGFVSQLPEDVHAIADAEGRIVDTLQVTANYVKQIWTTSDLIRPLSGCSCIIPICQLLSLQPHPYLALVP

>MdCOBL17

MGFLFLPMIRSVPKFVSFATLLVFVLSCTSTFIATEAYDPLDPNGNITIKWDILSWTPDGYVASVTLFNFQKYRHIQAPGWLLGWTWAKKEIIWNMVGGETTDQGDCSKFKTTIPHCCDKTPTIVDLLPGTPYNQQYANCCKGGVLSSWCIQ

>MdCOBL18

MEIRKCKSTLFASVVCFTIFSYAVAYDPFDPKGSINIRWDVVSWTSDGYVAAVTISNNQMYRPVTSPGWTLGWTWAKKEVIWSMAGAQATDQGYCSKFKGNIPHSCSKIPSVVDLPPTAPYNQRFSDCCKGGVLESLGQDPSAAVSAFQLSVGHSGTSNTRPPKNFFLLGPGPGYTCSAATIVPPSVSYSDGLRKTRAMMTWSLTCSYSQVLASQNPTCCVSLSSFYNPMITPCPCACGCQNKNNCDNDPSSVVSLKTVVQNASILCTDHMCPIRVHWHVKANYRYWRVRIAVANFKYRMNYTQWTLVAQHPNLNKLANVSSFFYKPLIHYNTNDTGMFYGIKNLNNLLMEAGTDGHVESELILQKNTKEFTLKQGWAFPLMIYFNGDECKMPLPDIYPLPNSPVTKDVTKNPNDGEDAPSSSIQLEGLDKKLGVCELCHYMPVEL

>MdCOBL19

MRAKAVAFRTWKAKQKSREIRETERIGHVVPSMRQPLAAYDPLDPNGNITVKWDIMSWTPDGYVAVVTMNNFQTYRHIMSPGWTLGWMWARKEVIWAAVGAEATKQGDCSWFKGNIPHCCKKTPTVVDLLPGAPYNQQFTNCCKGGVVSAWGQDPSTAVSSFQLSVGSAGTTKRTVRPPKNFTLLGPGPGYTCGRARVVPSTIFLSPDRRRRTQALMTWNVTCTYSQLLAKKYPSCCVSLSSFYNSTVVPCPSCTCGCHDKSNCVQSGSKIVSTVEDDAKSNVQPLPQCTRHMCPVRVHWHVMLNYDYWVKVSITNFNYRMNYTLWTLVIQHPNFINVTQVFSFDYKPLVAYDSINDTGMFYGLKFYNDHLMEAGKFGHVQEMLLKKDKNTFTLREGWAFPRKVYFNGDEQLPPPDVYPFLPNSAHETLLSFSPLISSFTFLFIVIW

>MdCOBL20

MGFLFLPMIRSVPKFVSFATLLVFVLSCTSTFIATAYDPLDPNGNITIKWDILSWTPDGYVASVTLFNFQKYRHIQAPGWLLGWTWAKKEIIWNMVGGETTDQGDCSKFKTTIPHCCDKTPTIVDLLPGTPYNQQYANCCKGVLSSWVQDPVNAAASFQISVGQAGTTNKTVRLPKNFTLNTPGPGRSYPLFVTVTWNVTCTYSQFLAQATPTCCVSLSSFYNDTVVPCQTCSCGCQSNATHPGSCVESNSPYLASVVSASNKHSYTPLVCTNHMCPIRVHWHVKENYQQYWRVKITVTNFNYRMNYSDWNLVVQHPNFDNLTQIFSYNYKSITPYGRINDTAMLWGVKFYNDVLMQSGPLGNVQSELLFQKGEAFTFDKGWAFPRRVYFNGDNCVMPPPDAYPRLPNSGFRQHVSLLTLIMTFLSTVAVMYAYV

>MdCOBL21

MGFLFLPMRSVPQFISFATLVFVLSCTTSFIATEAYDPLDPNGNITIKWDILSWTDGYVAAVTLFNFQKYRHIQAPGWSLGWTWAKKEVIWNMVGGQATEQGDCSRFKTTLPHCCKKTPTVVDLLPGTPYNQQYTNCCKGGVLSSWVQDPEKAVGSFQISVGQAGTTNKTVRLPKNFTLNTPGPGYTCARAKVVKPTKFISPDKRRVTQAMMTWNVTCTYSQILAQKTPTCCVSLSSFYNDTIVPCPNCSCGCQRNATHPGSCVEPDSPYLASVVSASNKNSYTPLVRCTAHMCPIRVHWHVKVNYKQYWRVKVTVTNFNYRMNYSDWNMVVQHPNFDNLTQIFSYNYKSITPYATINDTGMLWGIKFYNDILMQAGPLGNVQSELLFQKDEATFTFDKGWAFPRRVYFNGDNCVMPPPDAYPWLPNSGFRQYVSLLTLIMTCLSTIAVMAYA

>MdCOBL22

MEPCFSAVGSIAKLTSFTILLAFLLSCFTFTSTEAYDALDPTGNITIKWDVISWTPDGYVAVVTMFNFQQYRHIQAPGWTLGWTWAKKEVIWSMVGAQTTEQGDCSKYKGNVPHCCKKDPTVVDLLPGTPYNQQIANCCKGGVMQSWIQDPPNAISSFQVSVGAAGTTNKTVRLPKNFTLRAPGPGYSCGIAKIVKPTRFLTSDKRRWTQAMMTWNVTCTYSQFLAQKTPTCCVSLSSFYNETIVSCPTCACGCQNNATDPGSCVEPKNPYLASAVAGSGKSANTPLVQCTSHMCPIRVHWHVKLNYKDYWRVKVTITNFNYRMNYTLWNLVVQHPNFDNLTKIFSFNYKSLTPYVGLNDTAMLWGVKFYNDFLSEAGPLGNVQSELLFRKDTSTFTFEKGWAFPRRIYFNGDNCVMPPPDAYPWLPNASPKRIISILGPVMTAFASLVILLAY

>RoCOBL1

MASMTRPLIFFLNLLIILAALPISLSQTTEAPAPAADLCNGVFLSYSYTTGKKITPTDPARQPYRFESVLTVLNNGDEDLKSWKVFVGFKHGEYLVSASNAVLADGSSFPGSVENGTVFAGFPQTDLKTAIKTAGDLTQMQVQVNLIGTQFGVAPPGVPMPANITLANDGYSCPKASMQGKNEMQVCCTVDVTKKTNITVDEEFLPRQNGDLTIMYDVTRTYGSSYFAQVTISNHNPLGRLDSWKLSWDWMAQEFINTMRGAYPSVVDSSECIFGEQGTYYAELDFSNVLSCEKRPTIIDLPLTKANDTKFGLIPYCCRNGTILPTTMDPSKSKSSFQMEVYKMPPNINRSVILPPQNWAINGTLNPDYKCGPPVRVSPSHFPDASGLPVNSSAVASWQVVCNITQAKGASPRCCVSFSAYYNESVIPCKTCACGCPSNTAQTCSTTAPAMLLPPESLLVPFENRTVKAKAWAELKHLADPNPTPCGDNCGVSINWHLYTDYTKGWTARVTLFNWDETSFADWFAAVQLDEATPGFEKMYSFNATTVEMDGVNNTVFMQGNEGLNYLVGEVDGKNPQKDPRVPGKQQSVISFTKKLTPDINVAGRDGFPTKVFFNGEECSLPDIYPSSAYRKSSAMMMFPVLLMVVAFMFLQQ

>RoCOBL2

MGIIIIILSLIVLSSISLSYGYDPLDPYGNVTITWDFLDQSNPDTYDIRVSIFNMQQYRHVEVPGWKLEWTWNKSEVIWDMRGAEAVDQGDCSWFKGSGGGQLPHCCMKRPVIIDLLPGTPYNMQYSNCCKGGVLSSMNQDPSKYLSAFRMSVGNFHVNRQGSNDAFDKLNLTMPSHFNLGILGYTCGEPFLVPPTRFPADNRRRWTQALETWNITCTYSQSQASRAPKCCVSLSAFYNTTIVNCPRCSCGCQGLGGAKCVKSGDMPPPTVLQLPTSPPLVTCSQHMCPIRVHWHVKQSYKEYWRVKITITNLNFHKNYSDWNLVVQHPNLKSVTQLFSFNYHPLTTYGNINDTGMFWGIKFYNDMILTSGEQGNAQTEMLLHKDEGIFTFREGWTFPRRISFNGDECVMPPPDDYPTLPNGANTITAATLNLSLLFFSFLFLALIAPPLF

>RoCOBL3

METSCSMKIPWNLFFLLFLAVFATRVEVSYGQAADDDGGDDKPAMPEQEECDGIFLTYRFISREKELPHRKNVSAQAWAFKSEASVLNAGATTLKAWKMFIGFQHREILVAADGAVVVDGGEMPAAVGKNGSYFAGNPQTDLKNSIDTAGDYTQIQAKIAFKGTMFGLPAKATPMPKTIRIVNDGYKCPAAHIRGNTNMYVCCKPDPKFKVKKEDKDTKFMPKQNGDLSITYDVLQSFANNYLAQVTIDNVHPLGRLDHWNLTWEWQKNEFIQTMRGAYTHKKDPSECLYGPAGKIYKDFDFSKVMNCEKKPTITDLPPTMKDDDKVGKLPMCCRNGTILPTVMDKSKSQAIFQLQVFKLPPDDNRTALTPPQQWKIYGPVNPNYVCGPPLRIDPSRFPDPSGLQATTAAVASWQVVCNITKPKVPRCCVSFSAYFSESVVPCNTCACGCAEIQSSHCDRRAKAMLLPAEALLVPFVNRSEKAKAWAKIKHYDIPKKLPCGDNCGVSLNWHVDSDYSSGWTARLTLFNWGKEPFQDWYTAVQMNKAYEDYENVYSFNGTKMEKVKNTIMFQGLKGLNYLIEMRNATKEKDPRVPGKQQSVISFKKKHYHDIDIKAGEGFPSKVLFNGEECSLPKQFPKNGAEHQPRSNLLLVLFVAIASFVFMTDRFH

>RoCOBL4

MGFLFLPMINISSSTSLTILLLLFGLSCTSFTSTEAYDALDPTGNITIKWDVMTWTPDGYVAVVTIFNFQNYRHIQAPGWSLTWTWAKKEFIWSMVGAKATEKGDCSRFKGSIPHSCTKIPTIVDLLPSTPYNQQIANCCKGGVLGSVVQDPANSVAAFQITVGEAGTTNRTVMLPKNFTLSAPGYTCGPAKIVKPTRYITADGRRVTQAMMTWNVTCTYSQFLAQKIPTCCVSLSSFYNNTSVPCNKCSCGCQSNASKCIEPISSSVVSNSDKKSYPPLVQCTSHMCPIQISWQVEENHKQYWLVKLKITNFNYRMNYSDWNLVIQHPNFDKLTQIFRFNYKSLTPYAGINDTAMLWGIKFYNNILMQAGPLGSVESEFLFQKDGTNSTSSKGWPFPQRVYFNGDNCDMPPPDAYPLLPNT

>RoCOBL5

MSQIKVSIFLTFYALSNTFHLFIEVRPSLFLFVSEAYDILDPNGHIIISWDILYFTKDGYEAEVKIRNQQLFRNFEHPGWRLSWAWEGREIIMQLEGATISQMGYCAPHITPQPVSCMKTPNTGGQGKEERPVVQYSDEFFNHGSRITIRMRFTNPQQGSFASYERRAATVGEWHPEDHRSRKNKVEAIMSHRPEHMPRGVAWIGDACSIPSTDWIENLPDSTPELSLRKGIAYLFIADSSTFAFTCVIKCICTPNLKLFSLKIGCRAQLLRESDTELSSSSFTELPE

>RoCOBL6

MIKRAWSVLIPMFILLAFEFSNFTIAQDYGDDDQQPAAPPPAQDNCDGIFLAYYFISRHKIYPLLKNAEKQAWAFNSTATIINTGSYELKAWKMYIGFQHKEILVAANGAVLLNGDDFPAQVGNNGTYFSGSQQTDLKTSISTGGDYNQIQAQIQISGTQFGVKPSVTPMPRTIRLVNDGYKCPSPTKRKTAMSVCCVRNPKSKVSSTVTTKFLPRQKGDLSISYDITQAYGNNYQAQVTMENTSPLGRLDHWNISWEWMRGEFIYNMKGAYTHKIDYLPCIYGAPGQYYQQMDFSKVMNCEKKPVITDLPRERANDTEVGKIPYCCRNGSLLPPIMDATKSKSVFQLQVFKLPPDLDRTTLYPPEQWKVSGVINPDYKCGQPIRVDPSQFPDPSGLQASSVAIASWQVVCNMTRPQNKKNKCCVSFSAYYNESVIPCNTCACGCTNTKKCNPSARAMFLPPEALLVPFENRSAMAVAWAKIKHFHVPKPLPCGDNCGISINWHVLSDFKDGWTARVTLFNWMPMNFEDWFTAVEMKKGGGRGYENAYSFNGTRLSNLNNILFFQGLKGLNFLVMETNGTKPNGPKVPGKQQSVISFKKARTPGIEVAKGDGFPAKVFFNGEECALPTQIPAYGNRNHVNLIVVIFLSILTFVM

>RoCOBL7

MSPFFTIFLGILITITLRAEAFDALDLDGHITIRWDIMHFTTYGYKAKVKISNHQLYCNFEFLGWRLSWAWEGNKIVRQMEGARINTGCGGLLQGRPANLHLPRLKQDCDILQNLGRQCYEIERGDSSPSRRTIPCIMAPEMFYKCGSPTVNKRRFHLSRSSWIISCTYTPYAIPEHCAVARDLNRCSV

>RoCOBL8

MNGAFATKQGNCSSFKSDIPHSCVKDPEILDLMPDSSPENKSVDCCRGGLLDAWAINPSKSFSSFQIKVGNLQQNSFGHAPLYLTLMAPGLGYTCGPLLDVDPTVSLDIGGRRQVQVFRTWKTTCTYSSFIAAKTPVCCVSLSTFYNPTITSCPLCSCGCRLADKTTETCIRSSFPSITNDPQSDPDVVKCTDHMCPIRVHWHIMSNYVDYWRVKLTITNYNYHKNFSDWNVLVQHPGFSQKTTIYSFNSTMLPAGFRDEVALFWGIPYVNQELISTEKDQVGSVSTEMLLKKDMDSFTFSNGWAFPRRLYFNGETCQMPLPDTFPMLPNGAVSNFAKPSQCLFLLLTFLTTQALLGPWL

>RoCOBL9

MGFLFLPMNNISSSTSITMLLLVLFGLSCTSFTSTEAYDALDPTGNITIKWDVISWTPDGYVAVVNIYNHQKYRHIQAPGWSLRWTWAKKEFIWNMFGAQATEQGDCSRFIGYVPHSCTKIPTIVDLLPSAPYNQQVANCCKGAVLAAFQITVGEAGNTNRTVKLPKNFTLSAQANGYTCSHAKIVNSTRYITADGRRVTQAMMTWNVTCTYSQFLAQKIPTCCVSLSSFYNNTSALQQMPHSSSVVSNSGKKSYTPLVQCTSHMCPIQINWQVEENHKQFWLVQLKITNFNYRMNYTDWNLVIQPPNFDNLAQIFRFNYKSLTPYAGILAYFGYKEGQQILQTVENQIEIIHEEARKSRMVEERKAEILLKWLILCQHSSKHNEVSQTFSSSYI

>RoCOBL10

MGFLLSKVTSFATVLLFGLCCFSFTSTVNWVLEGVVVTFDAYDPLDPSGNITIKWDIKSWTADGYVAAVTIYNYQQYRHIQAPGWSLGWTWTKKEVIWDMVGGQATEQGDCSRFKTTPPHCCKRKPTIIDLLPGTPYNQQYSNCCKGGVLSSWIQDPANAAASFQLSVGQAGTSNRTVKHPQNFTLMGPGPGYTCGAAKVVKPTKYFTADKRRVSQAMLTWKLTCTYSQFLAQNTPACCVSLSSFYNDTVVPCPTCSCGCQNNITHPGSCVEPESPYLASVVSAADKNSPLVRCTRHMCPIRVHWHVKLNYREYWRVKVTVTNFDYRRNYSDWNMVIQHPNFDNLTQVFSFNSKSLTPYGTINDTAMLWGIKFYNDMLVHSGPLGNVQSELLFRKNPATFTFEKGWAFPRRISFNGDACVMPPPDAFPYLPNAGFGPYISLVTLIMISASTFAFVYACA

>RoCOBL11

MGLLLYKLTRFATVVLFGLFCCSFTSTGAYDPLDPNGNITIKWDILSWTPDGYVAVVTLFNFQQYRHIQAPGWSLGWTWAKKEVIWSMVGGQATEQGDCSRYKPTPPHCCKRTPTIVDLLPGTPYNQQYSNCCKGGVLSSWMQDPANAAASFQLSVGSAGTTNKTVKLPKNYTLMGPGPGYTCGAARVVKPTKFYTADKRRVSQAMMTWNVTCTYSQFLAQSAPSCCVSLSSFYNDTVVPCPTCSCACQNNITHPGSCVEPESPYLASVVSAADKNSPLVRCTSHMCPIRVHWHVKLNYNEYWRVKVTVTNFDYRRNYSDWNLVIQHPNFDNLTQVFSFNSESLTPYGTINDTAMLWGIKFYNDMLAQSGPLGNVQSELLFRKNPATFTFDKGWAFPRRIYFNGDTCVMPLPDAFPHLPNAGFRQYVSLLTVIMISASTFAFTYACA

>RoCOBL12

MEITKCKRSTLFASVMALAIFSSAVAYDPFDPYGSINIKWDVLSWTPDGYVATVSIRNNQMYRHFMSPGWSLGWTWAKKEVIWSMVGAKVTDQGDCSNFKGNTPHCCEKTPTVIDLLPGVPYNQQFTNCCKGGVLTSLGQDPSAAASAFQISVGLAGTSNKTVKPPKKFYLLGPGPGYTCSAATIVPSSVSFSPDGRSKTRAMMTWSLTCTYSQFLASKNPTCCVSLSSFYNPIITPCPSCACGCKDANNCMNDHMDSPLLQSKTSAKENAPQMLQCTEHKCPIRVHWHVKANYREYWRVKITITNFNYLMNYTQWTLAAQHPNLNKLANISSFLYKPLIQYNSINDTGMFYGIKDVNDLLREAGPKGYIYTELILKKDKKTFTLDQGWAFPLRVYFNGDECMMPLPDIYPSLPNSAFYSSYPISSSSSSTLAPFFIMANPAHKIHLVVTCSPWLKFTFLAMLFCVLVSPAAAYDPLDPTGNITIKWDVMSWTADGYVAAVIMNNFQMYRQIANPGWTLGWTWAKKEVIWTMVGAQTTEQGDCSKFKGNTPHCCKKTPTVVDLLPGVPYNQQFSNCCKGGVMASWGQDPSASVSAFQVSVGQGGTSNKTVKLPKNFTLLGPGPGYTCGPAKIVPSTVFLTPDHRRKTQALMTWNVTCTYSQFLASKNPSCCVSFSSFYNETVVPCPSCACGCRNKKDCIKGDSKLLKKAGINTPRKDNAPLLQCTHHMCPIRVHWHVKQNYKDYWRVKMAITNFNYRLNYTEWTLVAQHPNLNNITQVFSFDYKPLVAYQSINDTGMFYGMKFYNDLLMEAGPLGNVQSEVLMQKDKNTFTFKQGWAFPRKVYFNGDECMLPPPDAYPSLPNSGHVNSVSVLKMAASVLLIVFSIF

>RoCOBL13

MESQCLSATGSIAKLSSFTILLVFLVSCLTFTSTEAYDALDPTGNITIKWDVISWTPDGYVAVVTMFNFQQYRHIQAPGWTLGWTWAKKEVIWSMVGAQTTEQGDCSRYKGNVPHCCKKDPTVVDLLPGTPYNQQIANCCKGGVMQSWIQDPPNAVSSFQVSVGAAGTTNKTVRLPKNFTLKAPGPGYSCGQAKIVKPTRYLTADKRRITQAMMTWNVTCTYSQFLAQKTPTCCVSLSSFYNDTIVNCPTCACGCENNSTDPGSCVEPDSPYLASAVAGSGKSTNSPLVQCTSHMCPVRVHWHVKLNYKEYWRVKVTVTNFNYRMNYTLWNLVVQHPNFDNLTKIFSFNYKSLTPYAGLNDTAMLWGVKFYNDLLMQAGPLGNAQSELLFRKDASTFTFEKGWAFPRRIYFNGDNCVMPPPDAYPWLPNASPRQTISLLSLVITILASLVFLLAYA

>PaCOBL1

MSPRFLFGAQRSNAVLFIIFISSLVTSFSLSYGYDSLDPYANITITWDFHLQTGSTYDITVSIYNFQQFRHLDRPGWKLSWIWKDDDQVIWDMRGAEAMEQGNCSKFRSSPQLPHCCKKQPVIIDFLPGAPYNKQFSNCCKGGELSSMIQDSSKFLSAFQMNVGFGKTNNITEFMPTNFTLGIPGYTCGDAFLVSPTRSSPDGRRWVQTLETWNVTCMYSQFRASPSPKCCVSLSAFYNSTIVPCPKCSCGCQGLPGAKCLKSGEKSPHLLELPRVRENEEVPPLVTCSQHMCPIRVHWHVKQSYKEYWRVKITITNHNFVKNYSSWSLVVQHPNLRSVTQLFSFNYHPLNAYGNINDTGMFWGIKSYNDMLLASGQSGNAQSEMLLHKDPGIFTFREGWTFPRRISFNGDECVMPPPDEYPTLPNSATTSRPSLVFFSFLILVFVF

>PaCOBL2

MKRAWWVVITLMFALFGFAFSLTRAQDNGDGGDQQPAAPTPAQQDCDGIYMSYDFESRRKIYPFLKNAEKQAWAFKSTAHIVNTGTYELKAWKIYIGFQHKEILVGATGAVLMNGDDFPADVGNGTYLSGSGQTDLKTSISTAGDYTQIQAQIQFSGTMFGVKPSGVPMPKTIRLVNDGYKCPSPTNRKGSMYVCCVRNPKFKANVTKTKFLPRQKGDLTIAYDVIQAYENNYLAQVTMENCSPLGRLDHWNLTWEWMRGEFIYNMKGAYPHTIDYLNCIYGDAGKYYQQMDFSKVLNCEKKPVIGDLPREKANDTQVGKIPNCCRNGSILPPIMDQSKTKSAFQMQVFKLPPDLNRTALYPPERFKVDGVLNPEYKCGQPIRVDPAQFPDPSGLQATSLAIASWQIVCNITRAKTRKPKCCVSFSAYYNESVIPCNTCACGCSDTKKCNPKAHALLLPPETLLVPFENRTKKAIAWASIKHHHVPKPLPCGDHCSVSVNWHLLSDYKDGWTARITLFNWDKANFEDWFTAVQLKKATAGYEKAYSFNGTKIPKLDNIIFLQGLKGLNFLVAQRNGTKPEKDPKVPGKQQSVISFKKKHTPDIEVAKGDGFPTRVFFNGEECSLPTQLPLSYGNCHHVNFVVAICLSLLSSRKGLKICAAVTGILLIVILVVLVVLFVTILKPKEPSIFTKPVTLESFELVVIPVVRLNVSIGILITVKNPNYGGFKYENSTAHISYRGNVVAEAPIEKDTIPARATHNITTSVSILADKLVTDTHFVGDLIIAGVLNFTSETTLHGKVSLLKVFKMKATSYSECNISITIKTQSADSVCKSRVELK

>PaCOBL3

MAFQQVALLLLILATTSLFDAEGYDSLDPDANITIKWDIMAWTADGYVAYVALYNYQRYRHIRAPGWSIGWTWPKKEVIWSMLGAEATERGDCSSFKDAIPHNCEQTPTLVDLMPGAPYNQQVRSCCKGGVLRSWVQNGAPDVGFFTMTVGQSETTDTDVRLPTNFTLKAPGLGYTCGSASIVEPTKFPTADKRRVTQAFIIQHPNFDDMARSFSFNYKSLSLYGTINDTVVLWGINFFNDILMQSGPDGYIQTELLFYKDRAPFTLEDGWAFPRAVLFDGDYCQMPPPDIYPSLPKIESKLYYTSISVIVAAIVISVVIIFVTVYVHKLTVK

>PaCOBL4

MLGAEATKRGNCSSLKDAIPHNCEQSPTIVEQMPGASYNQQNRAPDVGFFTMIVGQSKTTNRDVRVPKNFTLKAPGPGYTCGSTSIVEPTKFPTVDKRRVTQALSKSMFIQPLYL

>PaCOBL5

MGPLFLPMIRSVSKLISFTILLLFGLACTSFTATAHHNVHCFAYFLPEAYDPLDPNGNITIKWDIISWTPDGYVAVVTIYNFQKYRHIQAPGWSLGWTWAKQEVIWNMVGGQATEQGDCSKFKTTIPHCCKKNPTVVDLLPGTPYNQQITNCCKGGVLTSWVQDPANAVGSFQLSVGHAGTTNKTVRVPKNFTLNAPGPGYTCGRANVVKPTKFVTPDKRRVTQAMMTWNVTCTYSQFLAQRTPTCCVSLSSFYNDTVVPCPACSCGCQSNATHPGSCVEPDSPYLASVVSASSKNSYMPLVRCTNHMCPIRVHWHVKLNYKKYWRVKVTVTNFNYRMNYSDWNLVVQHPNFDNLTQIFSFNYKSITPYATINDTAMLWGLKFYNDFLMQAGPLGNVQSELLFRKDQATFTFDKGWAFPRRIYFNGDNCVMPPPDAYPWLPNSGFRQYTSLLTLIMTSLSTAALMYVHA

>PaCOBL6

MRFFVSALFFLVIFSYAATFDPLDPNGNITIKWDIMSWTPDDYVAVVTLNNFQMYRHIMSPGWTLGWTWARREVIWSAVGAQATEQGDCSRFKGNIPHCCKTNPTFVDLLPGVPYNQQFTNCCKGGVLGAWGQDPSAAVSAFQLSVGSAGTSNRTVRLPRNFTLLGPGPGYTCGRARVVPPTIFLTPDRRRKTQALMTWNVTCTYSQILARKYPSCCVSLSSFYNDTVVPCPPCTCGCQTKDNCVKSDSKILSLVGVNTPKKDNKPLLQCTRHMCPVRVHWHVQRNYKDYWRVKVSITNFNYRMNYTLWTLVVQHPNLNNVTQVFSFDYKPLVPYESINDTGMFNGLKFFNDQLMEAGPFGNVQSEMLLRKDKNTFTLKEGWAFPRKVYFNGDECQLPPPDAYPFLPNSAYQNLLSFSTFISLFIFFLIAIW

>PaCOBL7

MNGAFATEQGNCSNYKTDIPHSCKNDPEILDLMPDASSDNKSEDCCRGGVLDALAINPSKSFSSFGIKVGNLGGAPFSGDPPLNLTLKAPGPGYTCGPLKSVDPTVSLDFGGRRQRQVFRTWKAICTYSSFLASETPVCCVSLSSFYNPEITSCPKCSCGCREVTDKNFGTCLRPAYPFTAMSSTGDNSGANFMGAQDRVKCTDHMCPIRVHWHVKTSYVDHWRIKLTITNYNYQRNFSNWNVLVQHPGLSMNPITYSFNSTLLQPGFRDAVALFWGIAFVNEELIATDEDGVGSVSTEILLEKDSESFTFKNGWAFPRRVYFNGENSDPLFLSLSLPLCDNRPAYPFTAMSSTGDNSGANFMGAQDRVKCTDHMCPIRVHWHVKTSYVDHWRIKLTITNYNYQRNFSNWNVLVQHPGLSMNPITYSFNSTLLQPGFRDAVALFWGIAFVNEELIATDEDGVGSVSTEILLEKDSESFTFKNGWAFPRRVYFNGENCEMPLPDTFPMLPNGTSKTQPPTHWLFLLLIFFISQTLLLHAPLNLKSDQLTPQACFECFPHNF

>PaCOBL8

MEIISKCIRSAFLVSVMFLTIVSHAVAYDSFDPNGRINIKWDVLSWTPDGYVAAVTIINYQMYRHITSPGWTLGWTWAKKEVIWSMVGAQATAQGDCSKFKANIPHSCKKTPSVVDLLPGVPVNQRFSDCCKSGVMASWGQDPSAAVSAFQLSVGHSGTSNKTVTPPKNFYLLGPGPGYTCSAATIVPPSVSFSPDGRRTTQAMMTWTLDCTYSQLLVSENPTCCVSLSSFYNPMITPCPSCACGCKDVNNNCINDSKDSRMLNQKALAKEDASMLQCTNHRCPIRVHWHVKANYRAYWRVKITITNFNYLMNYTQWTLVAQHPNFNKLANVSSFVYKPLIHYGSINDTGMFYGIKNHSDLLMEAGPEGSVQTELLLGKDMKAFTLEQGWAFPFKLYFNGDECKMPLPDIYPTLPNSAYANPISSSTLATTLLLSALLVFLCSCH

>PaCOBL9

MIFDKYTCLVLLAVSFILTIPHAAAYDPLDPNGNITLKWDVISWTSDGYVAVVTMNNFQMYRHIMSPGWTLGWVWAKKEVIWSMVGAQAVDQGDCTKFKGNIPHCCKKNPTVVDLLPGVPYNQQIANCCKAGAVASWGQDPSAAVSSFQLSVGRSGTSNKTVRLPKNFTLLGPGPGYTCSQAKIVRSTVFNSPDGRRKTQALMTWNVICTYSQLLASRYPTCCVSMSSFYNSTITPCPTCACGCRDKNKCIVSDSKLSSVVGIHTPTKDNTPLLQCTQHMCPIRVHWHVKTNYKQYWRVKITITNFNYRLNYTQWTLVVEHPNLNHITEVFSFVYKPLTPYQSKNDTGLFYGTKFYNDLLKEAGPEGNVQSELILEKNANTFTFKEGWGFPRKVYFNGDECMMPQPDEFPETETESSRFAGGEFMTIEREEEEEERRALPTQLTKSTLRWKHVALGLGSSSHSWLCYVAWYYLRQAAVTMNNFQMYRQIISPGWTLGWSWQKKEVIWTMVGAQTTEQGDCSKFKAAIPHCCKKTPTVVDLLPGVPYNQQFTNCCKGGVLASWGQDPTASVSAFQVSVGQAGTSNKTVKLPKNFTLLGPGPGYTCGPAKVVPSTVFLTTDRRRKTQALMTWNVTCTYSQFLASKNPSCCVSFSSFYNETIVPCPSCACGCHNKKNCIKSDSKLAHKTGINTPKKDNTPLLQCTHHMCPIRVHWHVKLNYKAYWRVKISITNFNYRMNYTEWTLVAQHPNLNNVTQVFSFDYKPLLPYESINDTGMFYGMKFYNDLLMEAGPSGNVQSEVLLQKDQNTFTFKQGWAFPRRVYFNGDECMLPPPDAYPALPNSAHVNSTSIFKMAASLLLILFSICFSLS

>PaCOBL10

MEPRCLSATGSIVKLSSFAILLFFLLSCFSFTSTEAYDALDPTGNITIKWDVISWTPDGYVAVVTMFNFQQYRHIQTPGWTLGWTWAKKEVIWSMVGAQTTEQGDCSRYKGTVPHCCKKDPTVVDLLPGTPYNQQIANCCKGGVMNSWIQDPANAISSFQVSVGAAGTTNKTVRLPKNFTLKAPGPGYSCGIAKIVKPTRFLTADKRRWTQAMSKYTKLPSPPVTWNVTCTYSQFLAQKTPTCCVSLSSFYNETIVSCPTCACGCQNNATDPGSCVEPNNPYLASAVSGPGKSTNAPLVQCTSHMCPVRVHWHVKLNYKEYWRVKVTITNFNYRMNYTLWNLVVQHPNFDNLTKIFSFNYKSLTPYAGLNDTAMLWGVKFYNDLLTQAGPLGNVQSELLFRKDTSTFTFEKGWAFPRRIYFNGDNCVMPPPDAYPWLPNSSPKQVISILHPAITIFVSLLFLLAYA

>PaCOBL11

MDKSQSQSIFQLQVFKLPPDDNRTALTPPQKWKINGALNPSYKCGPPIRIDPTQFPDPSG

LQATSASVASWQVVCNITKPAVPRCCVSFSAYYSDSVVPCSTCACGCKITETGKCSPREP

AMLLPAEALLVPFANRTEKAKAWAKIKHYDVPKKLPCPDNCGVSLNWHIDSDYSNGWTAR

LTLFNWGNDPFQDWYTAVKMNKAYEDYENVYSFNGTRMEKEVKSTIMFTGLKGLNYLIGI

KNGTDPEKNPMVPGKQQSMISFKKKHFHNIDIKGGEGFPTRVLFNGEECAIPKRFPKNNA

QHLNSNALMVLCIAILTFLFMTDRFH

>PaCOBL12

MDFNSMSCPLIFTLSFLLIFTALPSSFSQPTADAPAPASDSCNGVFLSYAYTTGAKLPPELKSNPKRQPYRFESVLTVLNNGLEDLKSWRVFVGFKNDEYLVSASNAVLADGTSLPGSVGNGTVFAGFPMTDLKTAIKTAGDLTQMEVQVKLLGTQFGVAPPKVPLPSNISLANDGFVCLNAVQGTNEMHVCCTVDTKFKTNITVDEKFLPRQNGDLTIMYDVTNTQDSNYGAQVTIANHNPLGRLDNWKLSWDWMADEFIFAMKGAYPSIVDSSDCIFGRQVSYYQALDFSTVLNCERRPTIIDLPPTRANDTVLGMVPNCCRNGTILPRSMDPSKSMSSFQIQVFKMPPDLNRSQFTPPQNWAINGTLNPDYKCGPPVRVSPSQFPDRSGLPVNSSAVASWQVVCNITQLKGASPRCCVSFSAFYNDSVIPCNTCACGCPSNTARTCSTTAPAMLLPPETLLVPFENRTVKAKAWAELKHLPVPNPTPCSDNCGVSINWHLYTDYSRGWSARVTLFNWDETSFVDWFAAVQMDKAGHGFEKMYSFNGSSLELNGVNNTVFMQGLEGLNYLVAETDAANPQKDPRVPGKQQSVISFTKKKTPGINVIGGDGFPTKVYFNGEECSLPSIYPSSGNRKSTPIMFSVLMVVAFMVM

>GhCOBL9A

MRFVISALFLFVVAAAYDPLDPNGNLTIKWDIVSWTPDGYVAVVTMNNFQMYRHIMSPGWTLGWTWAKKEVIWSMNGAQTTEQGDCSQFKGNVPHCCKKIPTVVDLLPGVPYNQQYSNCCKGGVLPAWGIDPQSAVSAFQISVGMAGTSNKTVKLPKNFTLLGPGPGYTCGPAKVVPSTTFLTQDRRRKTQALMTWNVTCTYSQFLARKNPSCCVSFSSFYNETITPCPTCACGCQNKNSCVKSGSKVLKRAGVNTPRKDNTPLLQCTHHMCPVRVHWHVKVNYKEYWRVKVSIINFNYRMNYTLWSLAVQHPNLNDVTQVFSFDYKPLVPYESINDTGMFYGMKFYNDLLMEAGPFGNVQSEVLLRKDKDTFTLKQGWAFPRKVYFNGDECMLPPPDTYPFLPNSAPHQQLFSGLAAIAAMVFLLVTVW

>OsBC1L4

MAVGGAGSSRSVAPCCCCAVLLAAALLFSAPATTEAYDALDPNGNITIKWDVMSWTPDGYVAVVTMFNYQQFRHIQAPGWQLGWTWAKKEVIWSMVGAQTTEQGDCSKFKGGTPHCCKKDPTVVDLLPGTPYNMQIANCCKAGVINTFNQDPSNAASSFQISVGLAGTTNKTVKLPKNFTLKAPGPGYTCGRAMIVRPTKFFTGDGRRATQALMTWNVTCTYSQFLAQKTPSCCVSLSSFYNDTIVNCPTCSCGCQNNGTSPGSCVNENSPYLQSAIDGPGKWTGQPLVQCTSHMCPIRIHWHVKLNYKEYWRVKITITNFNYRMNYTQWNLVAQHPNFNNITQLFSFNYKPLTPYGSKINDTAMFWGVKFYNDLLMQAGPLGNAQSELLLRKDSKDFTFDKGWAFPHRVYFNGDNCVMPPPDAYPWLPNASPLTKQPLTLSVLVFSIVLATLLAYA

>OsBC1L5

MPLRRRWAALLLGVAVVLAVAAAGARAQDYNNGGGGDGEDEEEEEKPSFKAQEACNGAFLTYTFTEREKEYPRTKNATAQAFAFKATATVLNTMTEDLKAWQMFVGFQHKEILVTVGGAVLLDGTDLPANVSGGVTFAGYPMANLLNSIETAGDLTQIQAQIDITGTQFGVKPPTAPMPRTIKLSNPGFRCPKPTHKQSVMYVCCVKDPKFKAKKVNTTTRYLPRQKADLTIAYDVLQAFGNNYMVQVTIDNWSPIGRLDNWNLTWEWKRGEFIYKMRGAYTLNKEGPACVYSPAAGYYKDFDFTPAYSCEKRPIVVDLPPEREKDKDVGNIPFCCKNGTLLPPTMDESKSRAVFQMQVFKLPPDLNRTALYPPQNWKIIGKLNPQYACRQPVRVSPVVFPDQTGLMSSTPAVASWQVACNITRPKRRAAKCCVSFSAYYDDSVVPCNTCACGCGGGGGGGNDTATCDADARATPLPPEALLIPFDNRTAKGRAWAKIKHRRVPNPMPCGDNCGLSVNWHIMNNYKSGWAARITIFNWQDYTFKDWFAAVTMRDHYSGYENVYSFNGTKMGAPFNNSIFMQGLPGLTYLEPITDGRTPEDPRVPGKQQSVISFSRKDAPNVNIAKGEGFPKRLYFDGEECALPDTIPKPSSAHRRAAAAASLGQIVMAVVLVMVVAVVDSLCL

>ATCOBL2

MNILFSRFSFLLLFLCSWTSFTFTTTEAYDALDPYGNITIKWDIMSWTGDGYVAVVTIFNFQQYRHIEAPGWQLGWSWMKKEVIWSMVGGQATEQGDCSKFKGNIPHCCKKTPAIVDLLPGTPYNQQISNCCRGGVISAWAQDPATAISSFQISVGQSGTTNTTVRAPRNITLKAPGPGYTCGPAKLVKPSRFISADKRRKTQSLLTWNITCTYSQFLARKTPTCCVSLSAFYNETIVPCPTCSCGCQNSSQAGTCVDPKIASVVPALGKNNLEPLLQCTQHMCPIRVHWHVKTSYKEYWRVKVAITNFNYNMNYSQWNLVVQHPNFDNLTKLFSFNYKPLNPYLNINDTAMLWGIKFYNDFLSQAGPVGNVQSELLFQKNPLEFTFEKGWAFPRRIYFNGDNCVMPPPDSYPWLPNASPNIATSPFVILLITFLSVLILM

>ATCOBL4

MRLLFSFCFFFFMIIFTATAYDPLDPSGNITIKWDIMSWTADGYVATVTMNNFQIYRHIQNPGWTLGWTWAKKEVIWSMVGAQTTEQGDCSKFKGNVPHCCKKTPTVVDLLPGVPYNQQFSNCCKGGVIGAWGQDPSAAVSQFQVSAGLAGTTNKTVKLPKNFTLLGPGPGYTCGPAKIVPSTVFLTTDKRRKTQALMTWNVTCTYSQFLARKHPSCCVSFSSFYNDTITPCPSCACGCENKKSCVKADSKILTKKGLNTPKKDNTPLLQCTHHMCPVRVHWHVKTNYKDYWRVKIAITNFNYRMNHTLWTLAIQHPNLNNVTQVFSFDYKPVSPYGSINDTGMFYGTKFYNDLLMEAGPSGNVQSEVLLQKDQKTFTFKQGWAFPRKVYFNGDECMLPPPDSYPFLPNSAQGNFASFSLTILLLLFISIW

>OsBC1

MELHRCSLLALLLAVTCSVAVAYDPLDPKGNITIKWDVISWTPDGYVAMVTMSNYQMYRQILAPGWTVGWSWAKKEVIWSIVGAQATEQGDCSKFKGGIPHSCKRTPAIVDLLPGVPYNQQIANCCKAGVVSAYGQDPAGSVSAFQVSVGLAGTTNKTVKLPTNFTLAGPGPGYTCGPATIVPSTVYLTPDRRRRTQALMTWTVTCTYSQQLASRYPTCCVSFSSFYNSTIVPCARCACGCGHDGYRGNGGGGKNARAGDGRSRRNSGGGGGHSGGTECIMGDSKRALSAGVNTPRKDGAPLLQCTSHMCPIRVHWHVKLNYKDYWRAKIAITNFNYRMNYTQWTLVAQHPNLNNVTEVFSFQYKPLLPYGNINDTGMFYGLKFYNDLLMEAGPFGNVQSEVLMRKDYNTFTFSQGWAFPRKIYFNGDECKMPPPDSYPYLPNSAPIGPPRSVAAAASAILVVLLLVA

>OsBC1L6

MALLLLRMGVSVALLVAFFSSLIPSSEAYDPLDPNGNITIKWDVLQWTPDGYVAVVSLYNYQQYRHIQSPGWKLGWVWAKKEIIWAMNGGQATEQGDCSKFKSNIPHCCKKDPEIVDLLPGTPYNMQIANCCKGGVLNSWAQDPANAIASFQVSVGQAGTTNKTVRVPRNFTLKSPGPGYTCGSAKVVRPTKFFSQDGRRTTQAHMTWNVTCTYSQIVAQRSPTCCVSLSSFYNDTIVNCPTCSCGCQNNKPGSCVEGNSPYLASVVNTHNKDSLTPLVQCTSHMCPIRVHWHVKVNYKEYWRVKITVTNFNYRMNYSQWNLVTQHPSFDNLTTIFSFNYKSLNPYGVINDTAMLWGIKYYNDLLMTAGPDGNVQSELLFKKDPKSFTFEKGWAFPRRVYFNGDNCVMPPPDAYPWLPNASTRVMSSILLPFITIWTALTFLMVYA

>PtrCOBL4

MDFDRFSSPFFSAVCFILVFSRAEAYDYLDPTGNITLKWDVISWTPDGYVAVVTMTNFQMYRHIMSPGWTVGWTWAKKEVIWSMVGAQATEQGDCSKFRMNIPHCCMKNPTVVDMLPGVPYNQQIANCCKGGVVSSWGQDPSAAVSSFQLSVGRSGTTNKTVRLPKNFTLLGPGLGYSCSQAKIVPSTVFLTSDGRRKTQAMMTWNVTCTYSQMLASKNPTCCVSMSSFYNSTITPCRTCACGCQNNSTCVPSDSMIQSVVGLNTPTKDNTPLVQCTKHNCPIRVHWHVKLNYKEYWRVKISITNFNYRLNYTQWTLVAQHPNLNNITQVFSFAYKPLLPYKSANDSGMFYGVKFFNDILMEAGPDGNIQTELILQKDKNTFTLNKGWAFPRKVYFNGDECMMPLPDDYPYLPNSAPKNVIGLSFVISAFFLLELIAFW

>ZmBK2

MGLRVRDSSALLALAVALACCSVAGSVTIFHSSENVNSVDRSMGDAHILSSPVAMAGGCCTHTGTCAVVAYDPLDPNGNITIKWDVISWTPDGYVAMVTMSNYQMYRHIMAPGWTLGWSWAKKEVIWSIVGAQATEQGDCSKFKGGIPHCCKRTPAVVDLLPGVPYNQQIANCCKAGVVSAYGQDPAGSVSALQVSVGLAGTTNKTVKLPRNFTLMGPGLGYTCGPAAVVPSTVYWTPDHRRRTQALMTWTVTCTYSQQLASRYPSCCVSFSSFYNSTIVPCARCACGCGGHGGHAGPGGCIEGDSKRALSAGVNTPRKDGQALLQCTPHMCPIRVHWHVKLNYKDYWRAKIAITNYNYRMNYTQWTLVAQHPNLDNVTEVFSFQYKPLQPYGSISEYNHRHLMT
